# Bidentate Chiral Bis(imidazolium)‐Based Halogen‐Bond Donors: Synthesis and Applications in Enantioselective Recognition and Catalysis†

**DOI:** 10.1002/anie.201915931

**Published:** 2020-03-18

**Authors:** Revannath L. Sutar, Elric Engelage, Raphael Stoll, Stefan M. Huber

**Affiliations:** ^1^ Fakultät für Chemie und Biochemie Ruhr-Universität Bochum Universitätsstrasse 150 44801 Bochum Germany

**Keywords:** chiral recognition, enantioselectivity, halogen bonding, haloimidazolium salts, organocatalysis

## Abstract

Even though halogen bonding—the noncovalent interaction between electrophilic halogen substituents and Lewis bases—has now been established in molecular recognition and catalysis, its use in enantioselective processes is still very rarely explored. Herein, we present the synthesis of chiral bidentate halogen‐bond donors based on two iodoimidazolium units with rigidly attached chiral sidearms. With these Lewis acids, chiral recognition of a racemic diamine is achieved in NMR studies. DFT calculations support a 1:1 interaction of the halogen‐bond donor with both enantiomers and indicate that the chiral recognition is based on a different spatial orientation of the Lewis bases in the halogen‐bonded complexes. In addition, moderate enantioselectivity is achieved in a Mukaiyama aldol reaction with a preorganized variant of the chiral halogen‐bond donor. This represents the first case in which asymmetric induction was realized with a pure halogen‐bond donor lacking any additional active functional groups.

## Graphical abstract

**Enantioselective process**: A dicationic bidentate halogen‐bond donor based on iodoimidazolium groups with rigidly attached chiral moieties allowed the NMR‐based enantiodiscrimination of a racemic diamine. It also enabled the first enantioselective reaction catalyzed by a “pure” halogen‐based Lewis acid.

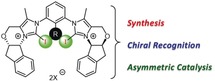

The last two decades have seen a steadily increasing interest in the use of noncovalent interactions in catalysis.^1^ One of these is halogen bonding (XB),^2^ which is similar to hydrogen bonding (HB) and has been applied in organocatalysis in a growing number of studies.^3^ Strong multidentate XB donors (halogen‐based Lewis acids) are typically based on two iodo(benz)imidazolium or iodotriazolium motifs^4^ and, particularly for more challenging substrates such as neutral carbonyl derivatives, a high degree of preorganization of the catalyst is crucial for good performance.4b, 4h

Almost all the XB donors used so far were achiral, and enantioselective versions of such catalytic processes are still in their infancy. Promising first cases were reported by the groups of Tan5a and Arai,5b, 5c but in these examples either XB is only one of several noncovalent interactions at work5a or further Lewis/Brønsted‐basic substituents on the XB donor make it difficult to assess the relative relevance of XB.5b, 5c Thus, overall, clear examples of purely XB‐based asymmetric induction are still lacking.^6^

Several reasons render the latter more challenging than HB‐based enantioselective processes: XB features a high directionality (with R‐X⋅⋅⋅LB angles of ca. 180°; LB=Lewis base), and this combined with the long R‐I and I⋅⋅⋅LB distances places a chiral backbone R quite far away from the substrate LB. In addition, and in contrast to HB, chiral bidentate XB motifs are not readily available but need to be designed from scratch, which leads to challenging synthetic routes. One strategy to provide a chiral environment close to the substrate is the incorporation of XB donor motifs in complex interlocked systems, which has been realized by Beer and co‐workers for [2]rotaxanes7a and [3]rotaxanes.7b An alternative approach is to equip XB donors with large chiral sidearms. After our group had reported that 1,2,3‐triazolium‐based bidentate XB donors can be easily modified with chiral substituents,8a Kanger and co‐workers presented more sophisticated versions of this motif, which, however, did not generate a noticeable enantiomeric excess in test reactions.8b–8d

An arguably more direct way to assess the performance of any new chiral XB donor for chirality transfer is the investigation of its binding behavior with a racemic mixture of XB acceptors.^7^, 8b, 9 Several cases have been reported by the Beer group, whereby chiral XB donors have been used for the enantioselective recognition of anions, typically carboxylates.^7^, ^9^ Neutral substrates are far less Lewis basic than anions and are thus more difficult to bind and differentiate. To the best of our knowledge, there is only one report on the ^1^H NMR enantiodiscrimination of a neutral compound by a chiral XB donor:8b Kanger and co‐workers used the above‐mentioned 1,2,3‐triazolium‐based XB donors to resolve the enantiomers of chiral thioureas. The difference in NMR shifts was very small, however, likely because the chiral sidearms can freely rotate around the C−N bonds which connect them to the XB donors.

In continuation of our work on XB‐based organocatalysts,4a‐4c, 4e, 4h, 8a, 10 we had also become interested in the synthesis of chiral derivatives, reasoning that the chiral motif should be connected to the XB donor as rigidly as possible to generate the best‐possible chirality transfer. Considering that bidentate imidazolium‐ and triazolium‐based XB donors had proven to be potent catalysts,^4^ we anticipated that literature‐known chiral N‐heterocyclic carbene precursor salts could serve as building blocks. Thus, 1,2,4‐triazolium or imidazolium motifs in which chiral sidearms are rigidly annelated to the heterocycles were selected to act as substituents and to generate a defined asymmetric environment. A 1,3‐modified benzene unit was employed as the core motif, so that bite angles similar to those of already established achiral bidentate catalysts could be obtained (Figure 1). In parallel to our work, Scheidt and co‐workers published a study on monodentate chiral analogues of the 1,2,4‐triazolium compounds,^11^ in which Brønsted acids were invoked as the actual catalytically active species.

**Figure 1:**
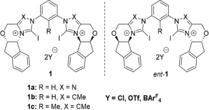
Chiral bidentate XB donors based on 1,2,4‐triazolium or imidazolium moieties.

Owing to the ease of synthesis of 1,2,4‐triazole‐containing chiral NHC precursors from aromatic amines and chiral amino alcohols (such as **2**, Scheme 1),^12^ we first focused on target structure **1 a** and synthesized its non‐halogenated chloride salt **4 a⋅Cl** (via an amidate intermediate **3**, see the Supporting Information). However, several attempts to obtain the corresponding XB donors with brominated or iodinated triazolium units were unsuccessful, likely because the products were rather unstable and moisture‐sensitive (yielding urea **5**, see the Supporting Information).

**Scheme 1:**
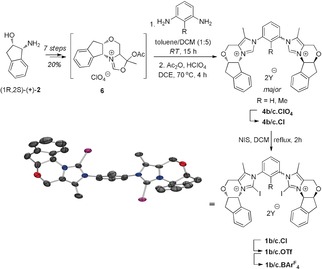
Synthesis of chiral bis(imidazolium)‐based XB donors and X‐ray structural analysis of *trans*‐**1 c⋅OTf** (anions and hydrogen atoms omitted).^14^ BAr^F^_4_=tetrakis[3,5‐bis(trifluoromethyl)phenyl]borate anion, DCM=CH_2_Cl_2_, NIS=*N*‐iodosuccinimide, Tf=trifluoromethanesulfonyl.

Consequently, the structurally closely related bisimidazolium derivate **1 b** was targeted (which features methyl groups in the backbone to allow the preparation of atropisomers in derivative **1 c**, see below). The synthesis is shown in Scheme 1. Acetal **6**,^13^ which can be obtained from commercially available (1*R*,2*S*)‐(+)‐*cis*‐1‐amino‐2‐indanol (**2**), was treated with *m*‐phenylenediamine, and the crude bisaminal thus obtained was subsequently dehydrated under acidic conditions to give the perchlorate salt **4 b⋅ClO_4_**. As a consequence of its low solubility in most solvents except acetone, it was transformed into the chloride salt through ion‐exchange chromatography for its purification by chromatography on silica gel. The aspired bisiodinated product **1 b** was obtained from this salt (**4 b⋅Cl**) by iodination with NIS and the anions were easily exchanged with triflate and BAr^F^_4_.^15^ A single set of signals in the ^1^H NMR spectra indicates that the XB‐donating moieties in these salts may still freely rotate.

Thus, we also prepared derivative **1 c**, in which a methyl group on the central benzene ring prevents this rotation of the iodoimidazolium groups, thereby allowing the isolation of *syn*‐*/anti*‐isomers. When higher substituted benzene precursors (with multiple methyl groups or a trifluoromethyl group) were used, only one imidazolium moiety could be formed. Separation of the *syn*‐*/anti*‐isomers of **1 c⋅OTf** was possible by preferential precipitation of the *anti*‐isomer from THF (see the Supporting Information for its X‐ray structure).^16^

With chiral bidentate XB donors in hand, we next screened their ability to differentiate the enantiomers of simple chiral bidentate Lewis bases **7**–**18** as benchmark compounds (Figure 2).

**Figure 2:**
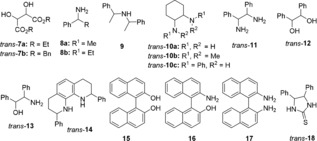
Chiral substrates tested as XB acceptors.

^1^H NMR analyses of the mixture of **1 b⋅BAr^F^**_**4**_ with tartrate esters **7** did not indicate any binding. Interestingly, chiral benzylamine derivatives **8** and **9** showed significant shifts of the ^1^H and ^13^C NMR signals upon complex formation, even though enantiodiscrimination was not observed. Although decomposition of XB donor **1 b⋅BAr^F^**_**4**_ was observed with (±)‐*trans*‐**10 a**, the analogous diamine *trans*‐**11** showed distinct separation of the NMR signals of the XB adducts of its enantiomers (see Figure 3 and below). Importantly, the corresponding hydrogen analogue (**4 b⋅BAr^F^**_**4**_) did not induce any differentiation of the ^1^H NMR signals of the two enantiomers (see the Supporting Information), thus indicating that the iodine substituents (and thus XB) are crucial for this recognition.

**Figure 3:**
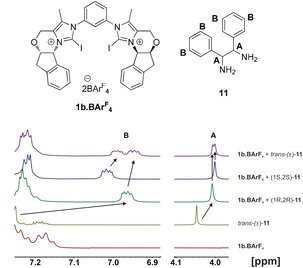
Enantiodiscrimination of *trans*‐**11** with **1 b⋅BAr^F^**_**4**_.

Identical experiments with structurally related diol **12** and amino alcohol **13** showed complex formation only for the latter case, but without enantiodiscrimination. In a similar fashion, (±)‐*trans*‐**10 b,** (±)‐*trans*‐**10 c,** and (±)‐*trans*‐**14** also did not show any differentiation under identical conditions. Inspired by the series *trans*‐**11** to *trans*‐**13**, a related set of binaphthyl derivatives were screened (**15**, **16**, and **17**). Consistent with our earlier observations, shifts in the ^1^H NMR signals of the amine‐containing compounds (**16** and **17**) were observed upon mixing, but no enantiodiscrimination was evident.

To elucidate the origin of the enantiodifferentiation in the complex of **1 b⋅BAr^F^**_**4**_ with the enantiomers of *trans*‐**11**, the binding constants of the diastereomeric complexes were determined by NMR titrations. The values obtained (211±9 m ^−1^ for (1*S*,2*S*)‐**11** and 349±15 m ^−1^ for (1*R*,2*R*)‐**11**) are similar enough to rule out a differentiation based on preferential binding. Instead, a different interaction of the XB donor backbone with the substrates must be responsible for the enantiodifferentiation. Indeed, the geometries of the two complexes, as obtained by DFT calculations (M06‐2X‐D318a, 18b maug‐def2‐TZVP,18c see Figure 4 and the Supporting Information), indicate that the enantiodifferentiation of *trans*‐**11** is the result of different spatial orientations of these substrates towards the XB donor: the phenyl ring in (1*S*,2*S*)‐**11** is closer to one terminal phenyl substituent of the XB donor compared to (1*R*,2*R*)‐**11**, and their orientation towards each other is markedly more parallel (Figure 4). This should lead to different anisotropic shifts of the *meta*‐protons (protons B, Figure 3) of the substrates induced by the aromatic ring current of the phenyl rings of **1 b⋅BAr^F^**_**4**_. NCI plots confirm the stronger interaction of the phenyl rings for (1*S*,2*S*)‐**11** (see the Supporting Information).

**Figure 4:**
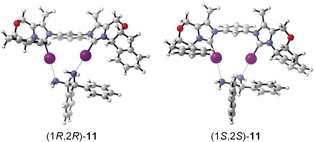
Structures of the adducts of **1 b⋅BAr^F^**_**4**_ with enantiomers of *trans*‐**11**, as obtained by DFT calculations (prepared with CYLview;^17^ for further details see the Supporting Information).

Since the NMR signals of the two enantiomers are relatively cleanly separated, we checked whether this system could be used to determine the enantiomeric excess of *trans*‐**11** (Figure 5). Preformed 4:1 and 1:4 mixtures of (1*S*,2*S*)‐**11** and (1*R*,2*R*)‐**11** were investigated by ^1^H NMR spectroscopy, and integration of the respective signals indeed yielded these ratios.

**Figure 5:**
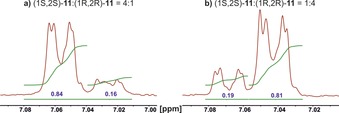
Determination of *er* of preformed mixtures of *trans*‐**11** with **1 b⋅BAr^F^**_**4**_.

As expected, the respective shifts of the two enantiomers show opposite results when enantiomeric XB‐donor *ent*‐**1 b⋅BAr^F^**_**4**_ is used. Similar enantiodiscrimination of *trans*‐**11** was achieved with more preorganized variant *syn*‐**1 c⋅BAr^F^**_**4**._ Notably, **1 b⋅BAr^F^**_**4**_ also shows enantiodiscrimination of thiourea *trans*‐(±)‐**18**, which is again markedly more pronounced than for H analogue **4 b⋅BAr^F^**_**4**_ (see the Supporting Information).

Finally, our aim was to establish a proof‐of‐principle case for enantioselective organocatalysis with XB donors **1 b/c⋅BAr^F^**_**4**_. The Mukaiyama aldol reaction of bidentate substrate ethyl glyoxylate (**19 a**) with TMS‐enolate **20** was chosen as a benchmark reaction,^19^ which does not proceed without catalyst (Figure 6). Just 5 mol % of XB donor **1 b⋅BAr^F^**_**4**_ induced a 91 % yield of highly functionalized product **21 a**, while the corresponding non‐halogenated compound **4 b⋅BAr^F^**_**4**_ was inactive, which clearly demonstrates the crucial role of halogen bonding. In line with our previous results,4b, 4h the preorganized variant of this XB donor (*syn*‐**1 c⋅BAr^F^**_4_) was found to be much more active. Molecular iodine,19d a potential decomposition product,4b led to less than 30 % product formation even when the same amount (5 mol %) was used. Other potential decomposition products are the corresponding bis‐(N‐heterocyclic carbenes), which may also be catalytically active.19e However, when such a dicarbene was generated in the presence of the starting materials by in situ deprotonation of **4 b⋅BAr^F^**_**4**_, no formation of the aldol adduct was observed. The absence of NHC catalysis was further corroborated by the fact that identical conversion was obtained when sulfur was added as a carbene trap to the reaction catalyzed by *syn*‐**1 c⋅BAr^F^**_4_.

**Figure 6:**
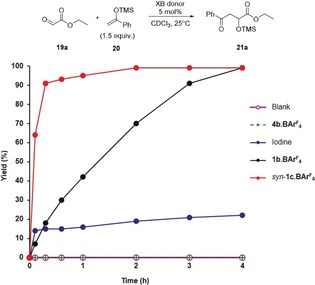
Kinetics of the Mukaiyama aldol reaction in the presence of various catalysts at 25 °C (based on ^1^H NMR yields). TMS=trimethylsilyl.

Since negligible enantioselectivity (2 % *ee*) was achieved at 4 °C, the temperature was lowered to −40 °C, which yielded no improvement (Table 1, entries 1 and 2). Two more bulky aldehydes and one ketone were then tested (entries 3–5) as electrophiles. Whereas <10 % conversion was observed with the ketone, both aldehydes produced good yields and a noticeable (identical) improvement in the enantiomeric excess (14 % *ee*). As the reaction times reached several days at −20 °C, we switched to preorganized XB donor *syn*‐**1 c⋅BAr^F^**_**4**_, which showed a similar enantioselectivity for the 2‐naphthyl‐substituted substrate but at a much faster rate (entry 6). This allowed the temperature to be lowered to −60 °C, which resulted in *ee* values of 30 % (entry 7). A further reduction to −70 °C led to much longer reaction times and a slight improvement in the enantioselectivity (33 %, entry 8).^20^ Importantly, monocarbonyl electrophiles such as benzaldehyde and *o*‐anisaldehyde resulted in either much lower enantioselectivities (up to 5 % *ee* at −20 °C for benzaldehyde) or low yields (<20 % at r.t. after 24 h for *o*‐anisaldehyde) in the presence of **1 b⋅BAr^F^**_**4**_, thus indicating that bidentate substrates may be necessary for XB coordination and asymmetric induction with these XB donors.

**Table 1:** Enantioselective Mukaiyama aldol reaction 
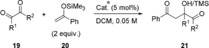

No	Catalyst*	R^1^	R^2^	*T* [°C]	*t* [h]	Yield^[a]^ [%]	*ee* ^[b]^ [%]
1	**1 b⋅BAr^F^**_**4**_	H	OEt	4	20	70	2
2	**1 b⋅BAr^F^**_**4**_	H	OEt	−40	48	60	2
3	**1 b⋅BAr^F^**_**4**_	Ph	OEt	25	60	<10	ND
4	**1 b⋅BAr^F^**_**4**_	H	Ph	−20	72	54	14
5	**1 b⋅BAr^F^**_**4**_	H	2‐Naph	−20	72	60	14
6	*syn‐* **1 c⋅BAr^F^**_**4**_	H	2‐Naph	−40	10	70	16
7	*syn‐* **1 c⋅BAr^F^**_**4**_	H	2‐Naph	−60	24	55	30
8	*syn‐* **1 c⋅BAr^F^**_**4**_	H	2‐Naph	−70	70	67	33

[a] Yields of isolated products after column chromatography (R=TMS for **1 b⋅BAr^F^**_**4**_, otherwise R=H). [b] Determined by chiral HPLC. * denotes the catalyst is chiral.

In conclusion, the synthesis of a dicationic bidentate halogen‐bond donor based on iodoimidazolium groups with rigidly attached chiral moieties was described. Two applications demonstrated its potential for chirality transfer and asymmetric induction: firstly, the first clear enantiodiscrimination of a neutral substrate by XB donors was described. The enantiomers of a suitably selected 1,2‐diamine showed different ^1^H NMR shifts upon complex formation, which was sufficient to allow the detection of enantiomeric excess. Determination of the binding constants and accompanying DFT calculations indicated that the discrimination originates from a different spatial orientation of the phenyl rings on the substrates towards those of the XB donor. Secondly, the first enantioselective reaction based on “pure” XB donors (devoid of further NCI‐forming or Lewis/Brønsted basic functional groups) was realized. Even though the enantioselectivity is still moderate, this constitutes an important step towards the further development of sophisticated XB‐based catalysis, and further studies in this regard are currently underway.

## Conflict of interest

The authors declare no conflict of interest.

## Supporting information

As a service to our authors and readers, this journal provides supporting information supplied by the authors. Such materials are peer reviewed and may be re‐organized for online delivery, but are not copy‐edited or typeset. Technical support issues arising from supporting information (other than missing files) should be addressed to the authors.

Supplementary
