# Supplementary material for: Bidentate Chiral Bis(imidazolium)‐Based Halogen‐Bond Donors: Synthesis and Applications in Enantioselective Recognition and Catalysis
Source: Angew Chem Int Ed Engl. 2020 Mar 18;59(17):6806–10. doi: 10.1002/anie.201915931 (PMC7187470; doi:10.1002/anie.201915931)
Supplement: Supplementary file 1 — Supplementary [file ANIE-59-6806-s001.pdf]

Angewandte Chemie
Eine Zeitschrift der Gesellschaft Deutscher Chemiker

Supporting Information

Bidentate Chiral Bis(imidazolium)-Based Halogen-Bond Donors: Synthesis and Applications in Enantioselective Recognition and Catalysis**

*Revannath L. Sutar, Elric Engelage, Raphael Stoll, and Stefan M. Huber**

anie_201915931_sm_miscellaneous_information.pdf

Supplementary Information

Contents

**1.**	**General remarks**	**S2**
**2.**	**Synthesis of XB-donors**	**S3**
**3.**	**Recognition and titration experiments**	**S12**
**4.**	**Catalysis of Mukaiyama aldol reaction**	**S23**
**5.**	**NMR spectra of XB Synthesis**	**S30**
**6.**	**DFT calculations**	**S49**
**7.**	**NCI plots**	**S57**
**8.**	**Single crystal X-ray data**	**S58**
**9.**	**References**	**S71**

1. General Remarks

All reagents and chemicals were obtained from *ABCR*, *Alfa Aesar*, *Carbolution*, *Merck*, *ChemPur*, *TCI and Sigma-Aldrich* and were used without further purification. Unless otherwise stated, all solvents used were of technical grade and were purified by distillation prior to use. Anhydrous solvents (DCM, ether and THF) were taken from *MBRAUN* (type: *MB SPS-800*) solvent drying system which were pre-dried by passing through an ALOX column followed by storing over 4 Å molecular sieve, and distillation. ITC measurements were performed on a VP-ITC from GE Healthcare. Thin layer chromatography was performed on *Merck TLC aluminum sheets* (silical gel 60, F254) and compounds were detected by fluorescence visualization under UV lamp (λ = 254 nm), iodine stain or using charring agents. Column chromatography was performed on silica gel (grain size 0.04-0.063 cm, *Macherey-Nagel Si60*). IR spectra were recorded on a *Shimadzu IR Affinity – 1S* spectrometer with a *Specac-Quest* ATR. The values were reported in cm^{-1} and are indicated as w (weak), m (medium), s (strong) or vs (very strong). NMR spectra were recorded on *AV-250, AV-300, AV-400* and *AV-600* instruments from *Bruker*. Chemical shifts (δ) are given in parts per million (ppm) with reference to the residual solvent signals (^{1}H and ^{13}C) or the internal standard (hexafluorobenzene, δ = -161.99 ppm) for ^{19}F and were analyzed with *MestReNova 9.0*. Multiplicities are given as s (singlet), brs (broad singlet), d (doublet), t (triplet), q (quartet), p (pentet), dd (doublet of doublet), m (multiplet) etc., and the coupling constants (*J*) are given in *Hz*. Optical rotations were measured on the *Antan Paar* automatic process polarimeter. Mass spectra were recorded on either a *Bruker Daltonics Esquire 6000* instrument (ESI) or a *VG Instruments Autospec / EBEE-Geometry* (EI). Elemental analysis was performed on a *vario Micro cube* from *Elementar Analysentechnik*. Single crystals were analysed on a *Rigaku* XtaLAB mini (Mo K)a *Rigaku* Synergy (Cu K_{α}). Data was recorded and reduced using the CrysalisPro[1] Software. Structures were solved using WinGX[2] in combination with ShelXT[3] and refined with shelXle[4] and ShelXL. Tables for the publication were generated using a modified version of Ciftab. Pictures of the structures were generated with Diamond 4.[5] HPLC analysis were performed with KANUR make AZURA instrument using chiral columns and the data was analyzed with the Clarity ChromPrep Software. The XB acceptors *trans*-**10b**,[6a] *trans*-**10c**,[6b] *trans*-**14**[6c] and *trans*-**18**[6d] were synthesized using the reported procedures. Hydrated aldehydes were dehydrated by heating with hot air gun under vacuum prior to the reaction. The anion exchange resigns Amberspec® 900 OTf and BAr^{F}_{4} ($B[3,5-(CF_3)_2Ph]_4$) were prepared from Amberspec® 900 OH and Cl form respectively.

2. Synthesis of XB-donor

2.1. Attempted synthesis of chiral bis(1,2,4-triazolium) XB (1a):

2.1.1. (-)-(5a*S*,5a'*S*,10b*R*,10b'*R*)-2,2'-(1,3-phenylene)bis(2,5a,6,10b-tetrahydro-4H-indeno [2,1-b][1,2,4]triazolo[4,3-d][1,4]oxazin-11-ium) chloride ((-)-4a.Cl):

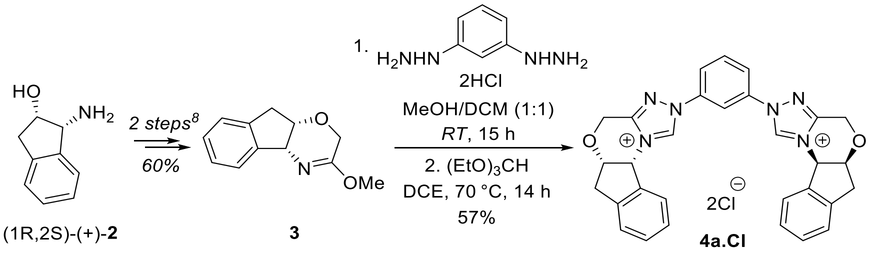

1,3-dihydrazinylbenzene dihydrochloride[7] (422 mg, 2 mmol) was added to the solution of (4a*R*,9a*S*)-3-methoxy-2,4a,9,9a-tetrahydroindeno[2,1-b][1,4]oxazine (**3**)[8] (894 mg, 4.4 mmol) in Methanol/DCM (1:1) (10 mL). This brown colored solution was stirred at room temperature for overnight. Solvent was evaporated and the residue was triturated with diethyl ether (2 × 10 mL) to get orange solid. It was dried under vacuum and was suspended in dichloroethane (10 mL). Triethyl orthoformate (1 mL) was added to it and the suspension was heated at 70 °C for 14 h. Solvent was evaporated and the residue was purified by column chromatography using methanol/DCM (1:4) as the solvent to give **4a.Cl** as a white solid (655 mg, 57%). R*f* = 0.18 (MeOH/DCM 1:4); ATR-IR (cm^{-1}): 3362.16 (m), 2910.89 (w), 1610.36 (m), 1584.33 (s), 1528.33 (s), 1099.05 (s), 1082.31 (s); ^{1}H NMR (300 MHz, $CDCl_3$): δ 13.82 (s, 2H), 9.30 (t, *J* = 2.1 Hz, 1H), 8.42 (d, *J* = 8.3 Hz, 2H), 7.99 (t, 6.3 Hz, 2H), 7.52 (t, *J* = 8.3 Hz, 1H), 7.33 – 7.27 (m, 6H), 6.41 (d, *J* = 4.2 Hz, 2H), 5.43 – 5.32 (m, 4H), 5.16 (d, *J* = 15.7 Hz, 2H), 3.34 (dd, *J* = 17.1, 4.9 Hz, 2H), 3.19 (d, *J* = 17.2 Hz, 2H); ^{13}C NMR (75 MHz, $CDCl_3$): δ 150.7, 142.7, 140.1, 136.5, 135.1, 132.1, 129.7, 128.2, 125.5, 125.2, 120.9, 111.0, 77.8, 63.0, 60.8, 37.7; EIMS for $C_{30}H_{26}Cl_2N_6O_2$, Calculated: $[M/Z+K]^+$ 290.2; Observed: 290.1; Elemental analysis, Calculated: C, 62.83; H, 4.57; N, 14.53; Observed: C, 62.80; H, 4.52; N, 14.56; $[\alpha]^{25}_{D}$ = -9.2 (*c* 1.0, $CHCl_3$).

2.1.2. (5a*S*,5a'*S*,10b*R*,10b'*R*)-2,2'-(1,3-phenylene)bis(2,4,5a,10b-tetrahydro-1H,6H-indeno[2,1b][1,2,4]triazolo[4,3-d][1,4]oxazin-1-one) (5):

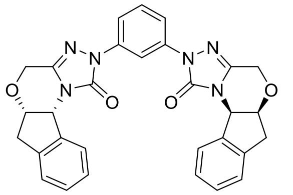

5

White solid. ATR-IR (cm^{-1}): 2915.60 (w), 1699.47 (vs), 1605.41 (s), 1588.48 (s), 1455.86 (s), 1441.75 (s), 1355.22 (s), 1339.23 (s), 1100.32 (s), 736.94 (s), 723.56 (s); ^{1}H NMR (300 MHz, $CDCl_3$): δ 8.65 (t, *J* = 2.1 Hz, 1H), 7.93 (dd, *J* = 8.2, 2.1 Hz, 2H), 7.88 (d, *J* = 7.0 Hz, 2H), 7.50 (t, *J* = 8.2 Hz, 1H), 7.33 – 7.20 (m, 6H), 5.35 (d, *J* = 4.0 Hz, 2H), 4.78 (d, *J* = 15.7 Hz, 2H), 4.63 – 4.52 (m, 4H), 3.30 (dd, *J* = 16.9, 4.6 Hz, 2H), 3.17 (d, *J* = 16.9 Hz, 2H); ^{13}C NMR (75 MHz, $CDCl_3$): δ 152.0, 141.0, 139.6, 138.9, 138.6, 129.8, 128.9, 127.7, 126.5, 124.9, 115.2, 108.7, 77.9, 61.1, 57.7, 37.7; EIMS for $C_{30}H_{24}N_6O_4$ Calculated: $[M]^+$ 532.2; Observed: 532.3. Elemental analysis, Calculated: C, 67.66; H, 4.54; N, 15.78; Observed: C, 67.62; H, 4.50; N, 15.71.

2.2. Synthesis of bis(imidazolium) XB:

2.2.1. (-)-(5a*S*,5a'*S*,10b*R*,10b'*R*)-2,2'-(1,3-phenylene)bis(3-methyl-2,5a,6,10b-tetrahydro-4H-imidazo[1,5-d]indeno[2,1-b][1,4]oxazin-11-ium) chloride (4b.Cl):

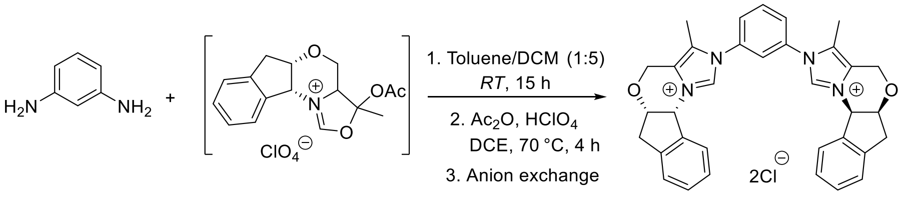

6 **4b.Cl**

In a 25 mL round bottom flask, (5a*S*,10b*R*)-3-acetoxy-3-methyl-3a,4,5a,10b-tetrahydro-3H,6H-indeno[2,1-b]oxazolo[3,4-d][1,4]oxazin-11-ium perchlorate (**6**)[9] (853 mg, 2.2 mmol) and benzene-1,3-diamine (108 mg, 1 mmol) were suspended in a solvent mixture of toluene and DCM (1:5) and it was vigorously stirred at room temperature for 15 h. The solvent was evaporated and pale yellow residue was triturated with diethyl ether (2 × 10 mL). The white solid obtained was dried under vacuum at room temperature. The crude bisaminal thus obtained was suspended in dichloroethane (13 mL); acetic anhydride (0.24 mL, 2.5 mmol) followed by perchloric acid (70%, 11 µl, 0.20 mmol) was added to it and the solution was heated to 70 °C for 2 h. Perchloric acid (70%, 11 µl, 0.20 mmol) was again added and heating was continued further for 2 h. Reaction

mass was cooled to room temperature and the solvent was evaporated to get dark red residue. It was triturated with diethyl ether (3 × 30 mL), dried and suspended in methanol. Amberlite IRA958 (chloride form) (3 g) was added to it and the suspension was gently stirred for 20 min. The resin was filtered off and washed thoroughly with methanol. The filtrate was evaporated on rotavapour to get brown solid which was purified by column chromatography using MeOH/DCM (1:4) as the eluent to obtain (-)-**4b.Cl** (410 mg, 68%) as a white solid. R_f = 0.2 (MeOH/DCM 1:4); ATR-IR (cm^{-1}): 3356.08 (m), 2916.01 (m), 1609.16 (m), 1541.52 (s), 1536.48 (s), 1101.35 (s), 735.88 (s); ^{1}H NMR (300 MHz, $CDCl_3$): δ 11.39 (s, 2H), 8.62 (t, *J* = 2.0 Hz, 1H), 7.96 – 7.84 (m, 3H), 7.83 – 7.77 (m, 2H), 7.36 – 7.28 (m, 6H), 6.10 (d, *J* = 4.5 Hz, 2H), 4.95 – 4.75 (m, 6H), 3.34 (dd, *J* = 17.0, 4.9 Hz, 2H), 3.24 (d, *J* = 15.4 Hz, 2H), 2.41 (s, 6H); ^{13}C NMR (63 MHz, $CDCl_3$): δ 139.9, 137.3, 135.9, 134.5, 132.0, 129.7, 128.0, 125.4, 125.2, 125.0, 124.4, 123.9, 77.4, 60.8, 59.3, 37.4, 9.6; EIMS for $C_{34}H_{32}Cl_2N_4O_2$, Calculated: $[M/Z+K]^+$ 303.2, Observed: 303.4; Elemental analysis, Calculated: C, 68.11; H, 5.38; N, 9.34; Observed: C, 68.09; H, 5.34; N, 9.30; $[\alpha]_D^{25}$ = -32 (*c* 1.1, $CHCl_3$); $[\alpha]_D^{25}$ of *ent*-**4b.Cl** = +34 (*c* 1.0, $CHCl_3$).

2.2.2. (5a*S*,5a'*S*,10b*R*,10b'*R*)-2,2'-(2-methyl-1,3-phenylene)bis(3-methyl-2,5a,6,10b-tetrahydro-4H-imidazo[1,5-d]indeno[2,1-b][1,4]oxazin-11-ium) chloride (4c.Cl):

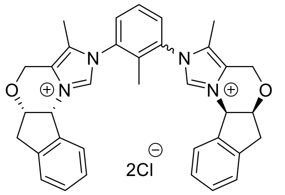

4c.Cl

Prepared from **6**[9] (853 mg, 2.2 mmol) and 2-methylbenzene-1,3-diamine (122 mg, 1 mmol) by using the same procedure as described above and purified by column chromatography using MeOH/DCM (1:6) as the eluent to obtain **4c.Cl** (330 mg, 54%) as white solid. R_f = 0.22 MeOH/DCM (1:6); ATR-IR (cm^{-1}): 3350.26 (m), 2959.18 (w), 2923.22 (w), 1636.33 (w), 1541.28 (m), 1101.38 (s), 1182.2 (s), 734.03 (vs); ^{1}H NMR (250 MHz, DMSO-d_6): δ 10.96 – 10.00 (m, 2H), 8.40 (s, 1H), 8.00 (t, *J* = 8.3 Hz, 2H), 7.93 – 7.80 (m, 1H), 7.72 – 7.57 (m, 2H), 7.45 – 7.21 (m, 5H), 6.06 (d, *J* = 12.8 Hz, 2H), 5.11 (d, *J* = 15.1 Hz, 2H), 5.00 – 4.75 (m, 4H), 3.44 (d, *J* = 15.8 Hz, 2H), 3.13 (d, *J* = 17.0 Hz, 2H), 2.21 – 1.86 (m, 9H); ^{13}C NMR (63 MHz, DMSO-d_6): δ 140.6, 137.1, 136.9, 133.5, 129.0, 127.1, 127.0, 125.3, 124.6, 123.3, 79.4, 77.0, 60.1, 59.0, 37.2,

12.8, 8.1, 7.9; EIMS for $C_{35}H_{34}Cl_2N_4O_2$, Calculated: $[M/Z+K]^+$ 310.2, Observed: 310.1; Elemental analysis, Calculated: C, 68.51; H, 5.59; N, 9.13; Observed: C, 68.49; H, 5.56; N, 9.10.

2.2.3. (5a*S*,5a'*S*,10b*R*,10b'*R*)-2,2'-(1,3-phenylene)bis(3-methyl-2,5a,6,10b-tetrahydro-4H-imidazo[1,5-d]indeno[2,1-b][1,4]oxazin-11-ium) triflate (4b.OTf):

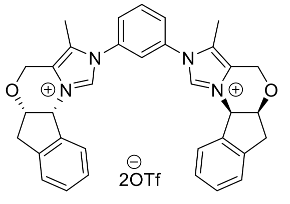

4b.OTf

The solution of **4b.Cl** (100 mg) in methanol (5 mL) was passed through a short (10 cm height) column of Amberspec® 900 (OTf form) resin and the column was eluted with methanol. The solvent was evaporated and the residue obtained was triturated with diethyl ether. It was dried under vacuum to get **4b.OTf** as white solid (125 mg, 91%). R_f = 0.2 (MeOH/DCM 1:7); ATR-IR (cm^{-1}): 2965.43 (w), 2929.79 (w), 1735.42 (m), 1541.32 (m), 1250.85 (m), 1222.34(m), 1026.89 (s), 633.26 (s); ^{1}H NMR (300 MHz, CD_2Cl_2): δ 9.74 (s, 2H), 8.06 (t, *J* = 2.0 Hz, 1H), 7.87 – 7.78 (m, 2H), 7.76 (dd, *J* = 2.8, 2.0 Hz, 1H), 7.59 (dt, *J* = 7.1, 1.2 Hz, 2H), 7.39 – 7.26 (m, 6H), 5.73 (d, *J* = 4.2 Hz, 2H), 4.92 – 4.76 (m, 6H), 3.27 (dd, *J* = 17.1, 4.5 Hz, 2H), 3.18 (d, *J* = 16.7 Hz, 2H), 2.21 (s, 6H); ^{13}C NMR (75 MHz, CD_2Cl_2): δ 140.6, 136.3, 135.8, 134.8, 132.1, 130.0, 128.8, 128.0, 125.9, 125.6, 125.4, 124.5, 121.05 (q, $^1J_{C,F}$ = 321 Hz), 77.7, 61.1, 59.8, 37.8, 8.9; ^{19}F NMR (235 MHz, $CDCl_3$): δ -78.50; Elemental analysis for $C_{36}H_{32}F_6N_4O_8S_2$, Calculated: C, 52.30; H, 3.90; N, 6.78; S, 7.76; Observed: C, 52.05; H, 3.64; N, 6.65; S, 7.69.

2.2.4. (5a*S*,5a'*S*,10b*R*,10b'*R*)-2,2'-(1,3-phenylene)bis(3-methyl-2,5a,6,10b-tetrahydro-4H-imidazo[1,5-d]indeno[2,1-b][1,4]oxazin-11-ium) tetrakis[3,5-bis9trifluoromethyl)phenyl] borate (4b.BAr^F$_4$):

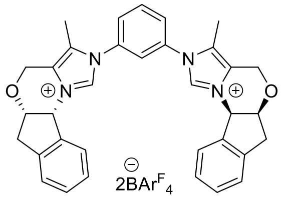

4b.BAr^F$_4$

Obtained by passing the solution of **4b.OTf** (100 mg) in methanol through the column of Amberspec® 900 (BAr^F_4 form) and eluting the column with methanol to get **4b.BAr^F$_4$** (260 mg,

96%). R_f = 0.22 (MeOH/DCM 1:7); ATR-IR (cm^{-1}): 2937.32 (w), 1609.23 (m), 1540.09 (m), 1349.96 (s), 1269.23 (s), 1099.32 (vs), 880.37 (s), 837.16 (s), 707.52 (s), 678.71 (s), 667.19 (s); ^{1}H NMR (300 MHz, CD_2Cl_2): δ 10.43 (s, 2H), 8.02 (t, *J* = 2.1 Hz, 1H), 7.90 (dd, *J* = 8.7, 7.6 Hz, 1H), 7.45 – 7.67 (m, 20H), 7.54 (s, 8H), 7.42 – 7.39(m, 4H), 7.31 (dq, *J* = 8.3, 3.9 Hz, 2H), 5.75 (d, *J* = 4.6 Hz, 2H), 5.00 – 4.86 (m, 4H), 4.77 (dd, *J* = 15.3, 1.3 Hz, 2H), 3.42 (dd, *J* = 17.1, 5.2 Hz, 2H), 3.31 (dd, *J* = 17.2, 2.0 Hz, 2H), 2.23 (s, 6H); ^{13}C NMR (75 MHz, CD_2Cl_2): δ 163.1, 162.5, 161.8, 161.1, 140.8, 135.2, 135.1, 134.4, 133.1, 130.9, 130.4, 129.5 (q, $^1J_{C,F}$ = 3 Hz), 129.05 (q, $^1J_{C,F}$ = 3 Hz), 128.7, 128.2, 126.8, 126.5, 125.7, 125.5, 124.6, 124.5, 123.2, 119.6, 117.9, 77.5, 61.4, 59.2, 37.4, 9.2; ^{19}F NMR (235 MHz, $CDCl_3$): δ -62.38; Elemental analysis for $C_{98}H_{56}B_2F_{48}N_4O_2$, Calculated: C, 52.20; H, 2.50; N, 2.48; Observed: C, 52.16; H, 2.45; N, 2.50; $[\alpha]^{25}_{D}$ = -13.1 (*c* 1.05, $CHCl_3$); $[\alpha]^{25}_{D}$ of *ent*-**4b.BAr^F$_4$** = +12.9 (*c* 1.2, $CHCl_3$).

2.2.5. (5a*S*,5a'*S*,10b*R*,10b'*R*)-2,2'-(1,3-phenylene)bis(1-iodo-3-methyl-2,5a,6,10b-tetrahydro-4H-imidazo[1,5-d]indeno[2,1-b][1,4]oxazin-11-ium) chloride (1b.Cl):

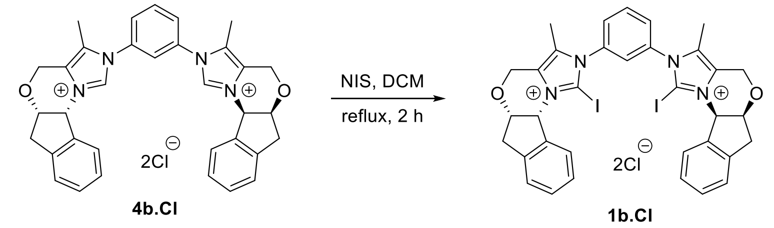

A 50 ml round bottom flask equipped with a reflux condenser was charged with **4b.Cl** (600 mg, 1 mmol), *N*-iodosuccinimide (495 mg, 2.2 mmol) and anhydrous DCM (20 mL). The solution was heated at reflux for 2 h. Solvent was removed on rotatory evaporator and the residue obtained was triturated with diethyl ether (2 × 25 mL) and THF (2 × 25 mL). It was then dissolved in minimal amount of acetonitrile and precipitated with diethyl ether. The procedure was repeated twice and the residue obtained was subjected to co-evaporation of $CHCl_3$, followed by drying under vacuum to get **1b.Cl** (650 mg, 76%) as a yellow solid. ATR-IR (cm^{-1}): 2914.59 (w), 1602.5 (w), 1490.77 (w), 1459.25 (w), 1430.61 (s), 1104.05 (s), 728.72 (s); ^{1}H NMR (300 MHz, CD_2Cl_2): δ 8.44 (s, 1H), 7.99 (t, *J* = 7.8 Hz, 1H), 7.79 (d, *J* = 8.0 Hz, 1H), 7.70 (d, *J* = 7.9 Hz, 1H), 7.59 (d, *J* = 7.3 Hz, 1H), 7.48 (d, *J* = 7.6 Hz, 1H), 7.39 (d, *J* = 7.2 Hz, 2H), 7.30 (d, *J* = 7.4 Hz, 1H), 7.26 – 7.18 (m, 2H), 7.09 (t, *J* = 7.3 Hz, 1H), 5.89 (s, 1H), 5.74 (s, 1H), 4.97 (d, *J* = 16.2 Hz, 2H), 4.87 (t, *J* = 7.5 Hz, 2H), 4.81 (s, 2H), 3.47 (t, *J* = 15.2 Hz, 2H), 3.19 (dd, *J* = 21.8, 16.8 Hz, 2H), 2.25 (s, 3H), 2.21 (s, 3H); ^{13}C NMR (75 MHz, DMSO-d_6): δ 140.9, 137.1, 136.1, 135.92, 135.90, 135.8, 132.1,

132.0, 131.9, 131.0, 130.5, 128.7, 127.0, 126.4, 125.5, 125.2, 125.1, 113.3, 78.1, 61.9, 59.4, 37.1, 9.7, 9.6; Elemental analysis for $C_{34}H_{30}Cl_2I_2N_4O_2$, Calculated: C, 47.97; H, 3.55; N, 6.58; Observed: C, 47.90; H, 3.51; N, 6.52.

2.2.6. (5a*S*,5a'*S*,10b*R*,10b'*R*)-2,2'-(2-methyl-1,3-phenylene)bis(1-iodo-3-methyl-2,5a,6,10b-tetrahydro-4H-11l4-imidazo[1,5-d]indeno[2,1-b][1,4]oxazine) bis(chloride) (1c.Cl):

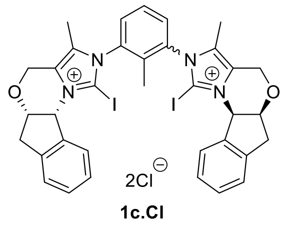

Following the above procedure, **4c.Cl** (612 mg, 1 mmol) was converted to **1c.Cl** (640 mg, 74%). ATR-IR (cm^{-1}): 2920.60 (w), 1601.56 (w), 1493.77 (w), 1459.25 (w), 726.15 (s); ^{1}H NMR (250 MHz, DMSO-d_6): δ 8.18 – 8.06 (m, 1H), 8.04 – 7.90 (m, 2H), 7.55 (d, *J* = 7.2 Hz, 1H), 7.48 – 7.19 (m, 7H), 6.03 – 5.76 (m, 2H), 5.05 (dd, *J* = 15.8, 5.4 Hz, 2H), 4.92 – 4.72 (m, 4H), 3.45 (d, *J* = 14.6 Hz, 2H), 3.08 (d, *J* = 16.6 Hz, 2H), 2.19 – 2.04 (m, 6H), 1.97 – 1.74 (m, 3H); ^{13}C NMR (63 MHz, DMSO-d_6): δ 141.2, 141.05, 140.97, 137.4, 137.2, 137.0, 135.7, 135.5, 135.4, 135.0, 134.0, 132.1, 131.8, 129.8, 128.9, 128.8, 127.7, 127.3, 126.8, 126.5, 126.4, 126.2, 125.7, 124.5, 124.2, 113.9, 78.1, 62.2, 62.0, 61.8, 59.6, 59.4, 37.1, 13.3, 12.6, 9.3, 9.2, 9.1; Elemental analysis for $C_{35}H_{32}Cl_2I_2N_4O_2$, Calculated: C, 48.58; H, 3.73; N, 6.47; Observed: C, 48.56; H, 3.66; N, 6.40.

2.2.7. (5a*S*,5a'*S*,10b*R*,10b'*R*)-2,2'-(1,3-phenylene)bis(1-iodo-3-methyl-2,5a,6,10b-tetrahydro-4H-imidazo[1,5-d]indeno[2,1-b][1,4]oxazin-11-ium) triflate (1b.OTf):

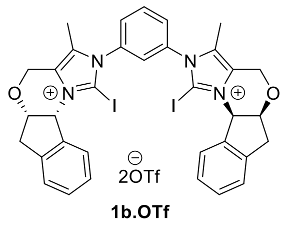

To the suspension of **1b.Cl** (600 mg) in methanol (20 mL), Amberspec® 900 (OTf form) resin (1.5 g) was added and it was stirred slowly for 30 min. The resin was filtered off and washed thoroughly with methanol. Filtrate was concentrated to get yellow solid and the crude product was triturated with pentane. It was dissolved in minimal amount of acetonitrile and was precipitated with diethyl ether. This process of dissolution and precipitation was repeated twice. Following the solvent removal through co-evaporation with $CHCl_3$, residue was dried under vacuum to get

1b.OTf (590 mg, 79%) as a pale yellow solid. ATR-IR (cm^{-1}): 3040.64 (w), 2920.87 (w), 1605.73 (w), 1496.78 (w), 1438.29 (w), 1254.79 (s), 1238.74 (s), 1219.24 (s), 1023.13 (vs), 734.13 (s), 633.20 (vs), 570.16 (s), 515.08 (s); ^{1}H NMR (300 MHz, CD_2Cl_2): δ 8.23 (t, *J* = 2.0 Hz, 1H), 8.07 – 7.89 (m, 2H), 7.72 (dt, *J* = 7.3, 2.0 Hz, 1H), 7.49 (d, *J* = 7.3 Hz, 1H), 7.44 (d, *J* = 7.5 Hz, 1H), 7.37 – 7.22 (m, 6H), 5.64 (d, *J* = 3.4 Hz, 1H), 5.40 (d, *J* = 3.5 Hz, 1H), 4.82 (td, *J* = 9.4, 7.1, 3.0 Hz, 6H), 3.30 – 3.18 (m, 2H), 3.12 (d, *J* = 16.8 Hz, 2H), 2.18 (s, 3H), 2.10 (s, 3H); ^{13}C NMR (63 MHz, CD_3CN): δ 142.2, 137.6, 137.6, 137.3, 137.2, 137.1, 133.8, 132.6, 132.4, 132.3, 130.7, 130.3, 129.6, 128.9, 128.87, 128.8, 128.7, 127.7, 127.0, 126.4, 126.3, 120.9 (q, $^{1}J_{C,F}$ = 320 Hz), 79.4, 64.0, 60.9, 38.3, 10.5, 10.2, 10.1; ^{19}F NMR (235 MHz, $CDCl_3$): δ -76.29; Elemental analysis for $C_{36}H_{30}F_6I_2N_4O_8S_2$, Calculated: C, 40.09; H, 2.80; N, 5.19; S, 5.94; Observed: C, 39.97; H, 2.76; N, 5.01, S, 5.85.

2.2.8. ***syn*-(5a*S*,5a'*S*,10b*R*,10b'*R*)-2,2'-(2-methyl-1,3-phenylene)bis(1-iodo-3-methyl-2,5a,6,10b-tetrahydro-4H-11l4-imidazo[1,5-d]indeno[2,1-b][1,4]oxazine) bis(trifluoromethanesulfonate) (*syn*-1c.OTf)**

The exchange of chloride anions of **1c.Cl** (500 mg) to the triflate was performed following the above procedure. To the product (mixture of *syn*- and *anti*-**1c.OTf**) thus obtained, THF (5 mL) was added. The precipitate of *anti*-**1c.OTf** was filtered off and the filtrate enriched in *syn*-**1c.OTf** was allowed to stand at room temperature for overnight. The solution was slowly decanted to remove the precipitate of *anti*-**1c.OTf** (if any). Evaporation of the solvent from the filtrate yielded *syn*-**1c.OTf** as a faint yellow solid (250 mg, 39%).

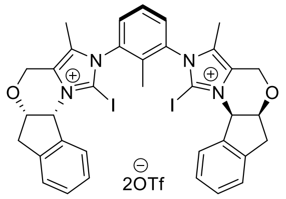

syn-**1c.OTf**

^{1}H NMR (300 MHz, CD_2Cl_2): δ 8.04 – 7.97 (m, 1H), 7.87 (t, *J* = 8.0 Hz, 1H), 7.80 – 7.69 (m, 1H), 7.55 – 7.49 (m, 1H), 7.46 – 7.40 (m, 5H), 7.38 (s, 1H), 7.32 (td, *J* = 4.8, 4.3, 2.7 Hz, 2H), 5.75 (d, *J* = 3.9 Hz, 1H), 5.67 (d, *J* = 3.5 Hz, 1H), 5.07 – 4.83 (m, 6H), 3.42 (dq, *J* = 15.4, 4.3 Hz, 2H), 3.24 (dd, *J* = 16.9, 6.0 Hz, 2H), 2.20 (t, *J* = 4.0 Hz, 6H), 2.03 (s, 3H); ^{13}C NMR (63 MHz, CD_3CN): δ 142.26, 142.22, 137.7, 137.6, 136.8, 136.7, 136.3, 133.4, 133.2, 131.2, 130.3, 130.2, 130.2, 129.3, 129.1, 128.0, 127.7, 127.0, 126.9, 126.5, 125.8, 121.8 (q, $^{1}J_{C,F}$ = 320 Hz), 79.4, 79.3, 64.1,

63.9, 60.9, 38.3, 13.9, 10.21, 10.07, 10.02; ^{19}F NMR (235 MHz, CD_2Cl_2): δ -78.47; Elemental analysis for $C_{37}H_{32}F_6I_2N_4O_8S_2$, Calculated: C, 40.67; H, 2.95; N, 5.13; S, 5.87; Observed: C, 40.58; H, 2.90; N, 5.12; S, 5.85.

2.2.9. ***anti*****-(5a*S*,5a'*S*,10b*R*,10b'*R*)-2,2'-(2-methyl-1,3-phenylene)bis(1-iodo-3-methyl-2,5a,6,10b-tetrahydro-4H-11l4-imidazo[1,5-d]indeno[2,1-b][1,4]oxazine) bis(trifluoromethanesulfonate) (*anti*-1c.OTf):**

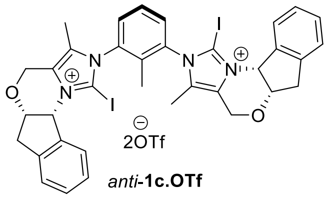

anti-**1c.OTf**

Yield = 220 mg (34%), White solid. ATR-IR (cm^{-1}): 2920.23 (w), 2839. 37, 1482.45 (w), 1459.71 (w), 1248.29 (vs), 1223.28 (s), 1026.63 (vs), 740.18 (s), 633.33 (vs), 515.11 (s); ^{1}H NMR (250 MHz, CD_3CN): δ 7.95 (dd, *J* = 9.1, 6.7 Hz, 1H), 7.88 – 7.80 (m, 2H), 7.48 – 7.34 (m, 6H), 7.26 (dd, *J* = 8.1, 6.2 Hz, 2H), 5.77 (d, *J* = 3.5 Hz, 2H), 5.03 – 4.77 (m, 6H), 3.45 (dd, *J* = 17.0, 4.2 Hz, 2H), 3.18 (d, *J* = 17.0 Hz, 2H), 2.15 (s, 6H), 1.88 (s, 3H); ^{13}C NMR (63 MHz, CD_3CN): δ 142.3, 137.6, 136.5, 135.4, 133.6, 131.4, 130.4, 130.3, 129.7, 127.9, 127.0, 125.8, 120.8 (q, $^{1}J_{C,F}$ = 321 Hz), 98.2, 79.3, 64.0, 61.0, 38.2, 13.6, 10.1; ^{19}F NMR (235 MHz, CD_3CN): δ -79.20; Elemental analysis for $C_{37}H_{32}F_6I_2N_4O_8S_2$, Calculated: C, 40.67; H, 2.95; N, 5.13; S, 5.87; Observed: C, 40.48; H, 2.87; N, 5.10; S, 5.65.

2.2.10. (5a*S*,5a'*S*,10b*R*,10b'*R*)-2,2'-(1,3-phenylene)bis(1-iodo-3-methyl-2,5a,6,10b-tetrahydro-4H-imidazo[1,5-d]indeno[2,1-b][1,4]oxazin-11-ium) tetrakis[3,5-bis(trifluoromethyl) phenyl]borate (1b.BAr^{F}$_4$):

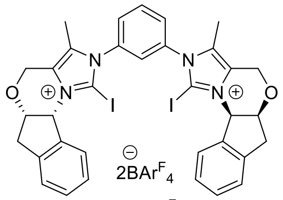

1b.BAr^F$_4$

In an argon atmosphere, the mixture of **1b.OTf** (500 mg, 0.46 mmol) and TMABAr^F$_4$ (4.3 g, 4.6 mmo 10 equiv.) was stirred vigorously in anhydrous THF (25 mL) for 4 days. The solvent was evaporated and the residue was extracted with diethyl ether. Filtrate was concentrated and the residue was extracted with $CHCl_3$. Solvent was evaporated, solid obtained was dissolved in

minimum amount of DCM and the product was precipitated by the addition of pentane. This process is repeated until filtrate becomes colorless. Finally the precipitate was sonicated in the presence of pentane. The solid obtained was dried under vacuum to get **1b.BAr^{F}4** (970 mg, 84%). ATR-IR (cm^{-1}): 2926.10 (w), 1610.46 (w), 1354.35 (s), 1273.98 (vs), 1109.64 (vs), 886.66 (m), 839.61 (m), 712.01 (s), 681.08 (s), 670.12 (s); ^{1}H NMR (200 MHz, CD_3CN): δ 8.09 (t, *J* = 7.6 Hz, 1H), 7.98 (s, 1H), 7.97 – 7.55 (m, 24H), 7.53 (d, *J* = 7.6 Hz, 2H), 7.49 – 7.38 (m, 5H), 7.37 – 7.23 (m, 3H), 5.78 (brs, 2H), 4.95 (d, *J* = 15.5 Hz, 2H), 4.79 (d, *J* = 14.0 Hz, 4H), 3.45 (dd, *J* = 16.8, 3.6 Hz, 2H), 3.18 (d, *J* = 17.0 Hz, 2H), 2.19 (d, *J* = 10.5 Hz, 6H); ^{13}C NMR (75 MHz, CD_2Cl_2): δ 163.2, 162.5, 161.8, 161.2, 141.3, 141.2, 136.2, 135.2, 134.9, 132.5, 132.3, 131.0, 130.9, 130.8, 130.7, 130.4, 129.9 (q, $^{1}J_{C,F}$ = 3 Hz), 129.5 (q, $^{1}J_{C,F}$ = 3 Hz), 129.1 (q, $^{1}J_{C,F}$ = 3 Hz), 128.7 (q, $^{1}J_{C,F}$ = 3 Hz), 127.9, 127.6, 127.0, 126.8, 124.7, 124.5, 123.2, 119.6, 118.04, 117.98, 117.97, 117.92, 78.4, 78.2, 64.2, 59.7, 59.5, 38.0, 10.6, 10.3; ^{19}F NMR (235 MHz, $CDCl_3$) δ -62.75; Elemental analysis for $C_{98}H_{54}B_2F_{48}I_2N_4O_2$, Calculated: C, 46.95; H, 2.17; N, 2.23; Observed: C, 46.85; H, 2.20; N, 2.50; $[\alpha]_D^{25}$ = -10.1 (*c* 1.1, $CHCl_3$); $[\alpha]_D^{25}$ of *ent*-**1b.BAr^{F}4** = +9.8 (*c* 1.0, $CHCl_3$).

2.2.11. ***syn*-(5aS,5a'S,10bR,10b'R)-2,2'-(2-methyl-1,3-phenylene)bis(1-iodo-3-methyl-2,5a,6,10b-tetrahydro-4H-imidazo[1,5-d]indeno[2,1-b][1,4]oxazin-11-ium)tetrakis[3,5-bis(trifluoromethyl) phenyl]borate (*syn*-1c.BAr^{F}4):**

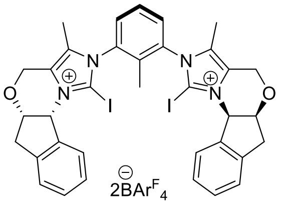

syn-**1c.BAr$^{F}_{4}$**

It was prepared using the same procedure described above from *syn*-**1c.OTf** (300 mg, 0.27 mmol) and TMABAr^{F}4 (2.52 g, 2.7 mmol). Yield = 500 mg (73%), off white solid. ATR-IR (cm^{-1}): 2926.05 (w), 1611.09 (w), 1354.78 (s), 1273.18 (vs), 1109.97 (vs), 886.78 (m), 839.06 (s), 712.16 (s), 681.31 (s), 669.22 (s); ^{1}H NMR (300 MHz, CD_2Cl_2): δ 7.89 (t, *J* = 7.9 Hz, 1H), 7.83 – 7.60 (m, 19H), 7.55 (s, 8H), 7.49 – 7.42 (m, 3H), 7.41 – 7.28 (m, 3H), 7.21 (d, *J* = 4.4 Hz, 1H), 5.70 (dd, *J* = 9.7, 4.2 Hz, 2H), 4.97 – 4.72 (m, 6H), 3.48 (d, *J* = 17.7 Hz, 2H), 3.33 (d, *J* = 17.4 Hz, 2H), 2.18 – 2.02 (m, 6H), 1.89 – 1.79 (m, 3H); ^{13}C NMR (75 MHz, CD_2Cl_2): δ 163.1, 162.5, 161.8, 161.1, 141.2, 141.1, 135.7, 135.2, 134.9, 134.5, 133.0, 132.9, 132.1, 130.9, 130.8, 130.6, 130.6, 130.4, 130.3, 130.1, 129.9 (q, $^{1}J_{C,F}$ = 3 Hz), 129.5 (q, $^{1}J_{C,F}$ = 3 Hz), 129.05 (q, $^{1}J_{C,F}$ = 3 Hz), 128.6

(q, $^{1}J_{C,F}$ = 3 Hz), 127.82, 127.0, 126.9, 126.8, 124.7, 124.2, 123.2, 119.6, 118.0, 117.9, 117.86, 78.3, 78.2, 64.1, 64.0, 59.8, 59.6, 38.0, 27.3, 13.7, 10.2, 10.1; Elemental analysis for $C_{99}H_{56}B_2F_{48}I_2N_4O_2$, Calculated: C, 47.17; H, 2.24; N, 2.22; Observed: C, 46.90; H, 2.34; N, 2.20; $[\alpha]_D^{25}$ = -17.6 (*c* 1.1, $CHCl_3$).

3. Recognition and titration experiments

3.1. NMR studies of chiral recognition

All chiral recognition studies were performed by mixing equivalent amounts of the solutions of donor (10 mM) and acceptor (10 mM) at room temperature (25 °C).

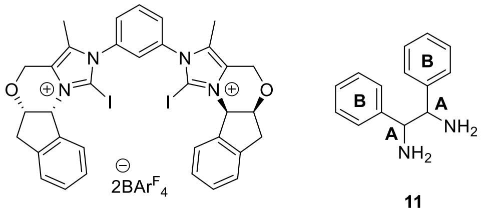

1b.BArF4　**11**

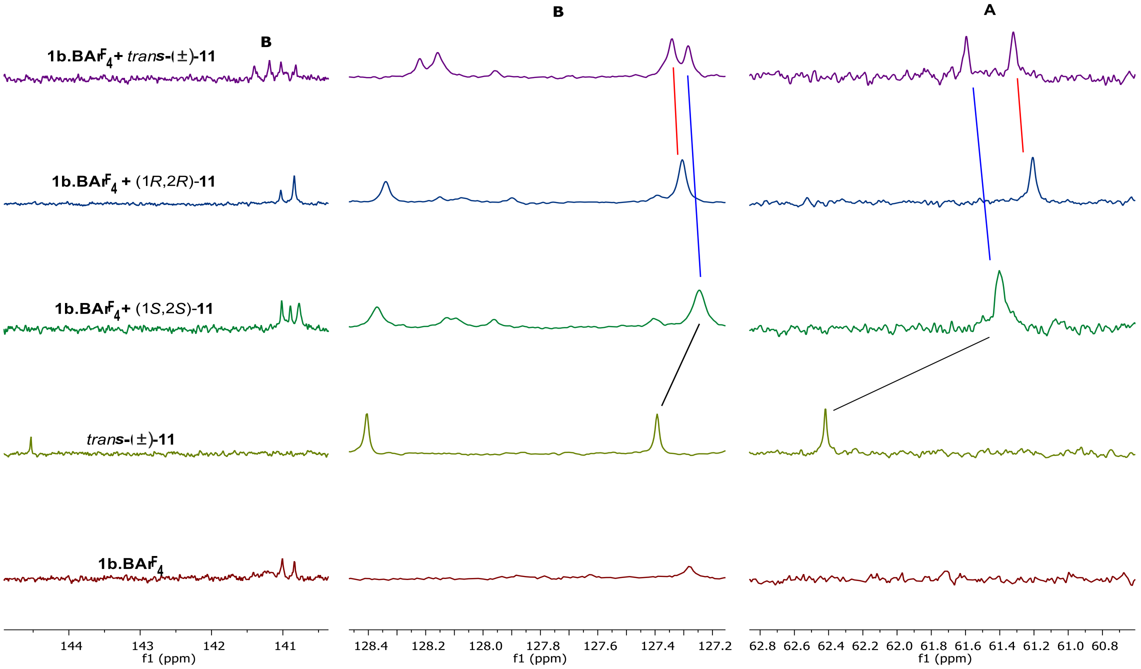

Figure S1. Shift in ^{13}C NMR (CD_2Cl_2, 125 MHz) signals of *trans*-**11** on interaction with **1b.BAr^F$_4$**.

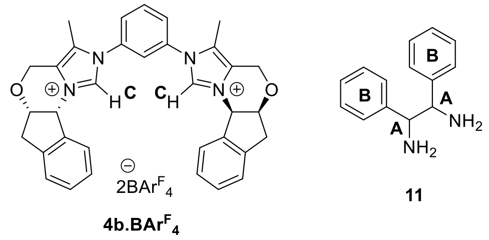

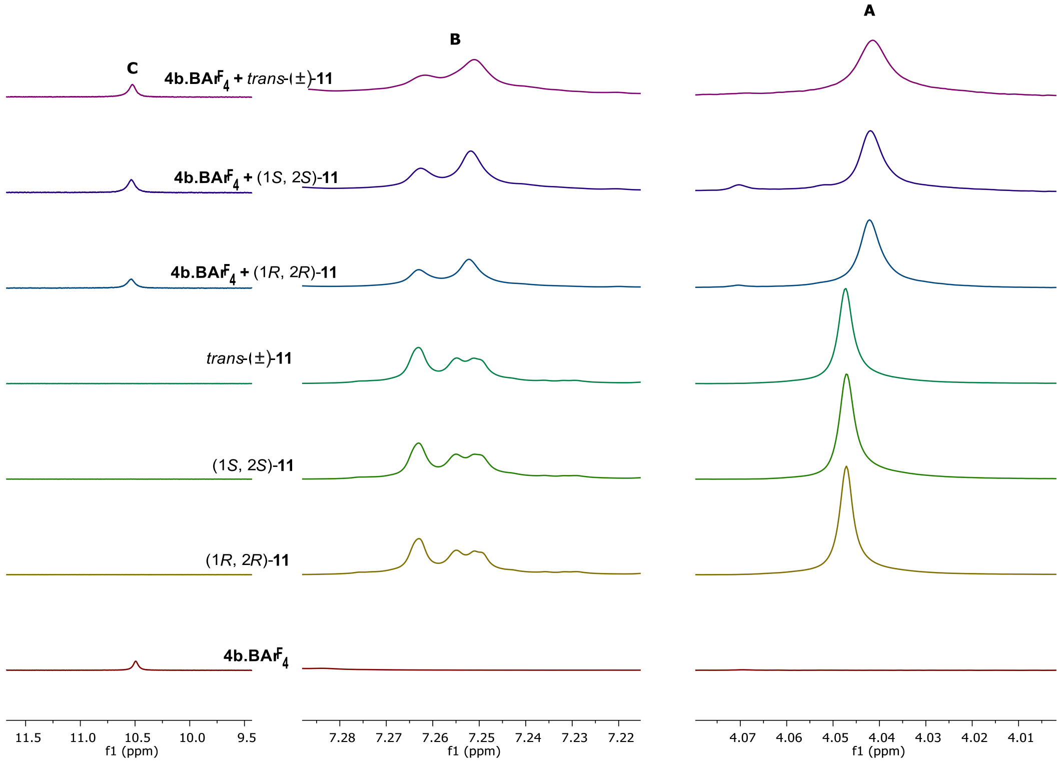

Figure S2. Attempted resolution of *trans*-**11** with **4b. BAr^F$_4$** by ^{1}H NMR (CD_2Cl_2, 400 MHz).

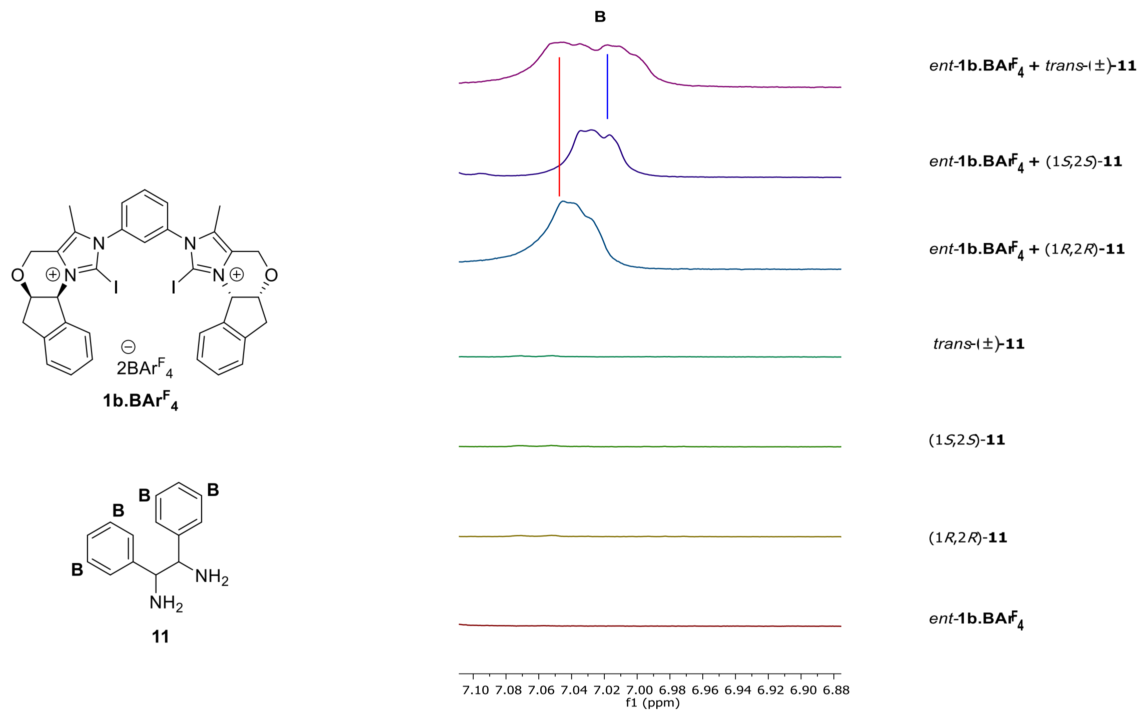

Figure S3. Resolution of *trans*-**11** with *ent*-**1b.BAr^{F}$_4$** using ^{1}H NMR (CD_2Cl_2, 400 MHz).

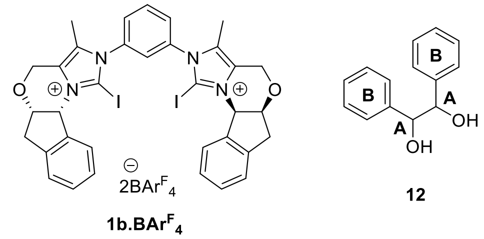

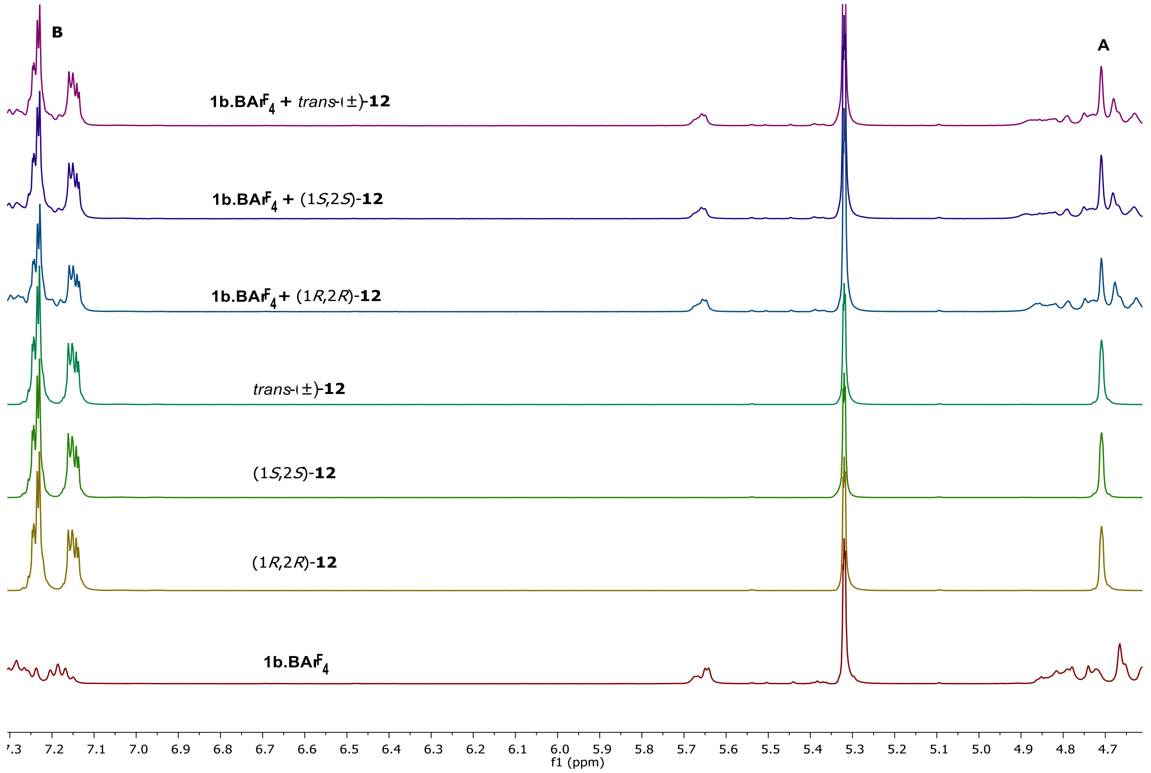

Figure S4. Attempted resolution of *trans*-**12** with **BAr^F$_4$** by ^{1}H NMR (CD_2Cl_2, 400 MHz).

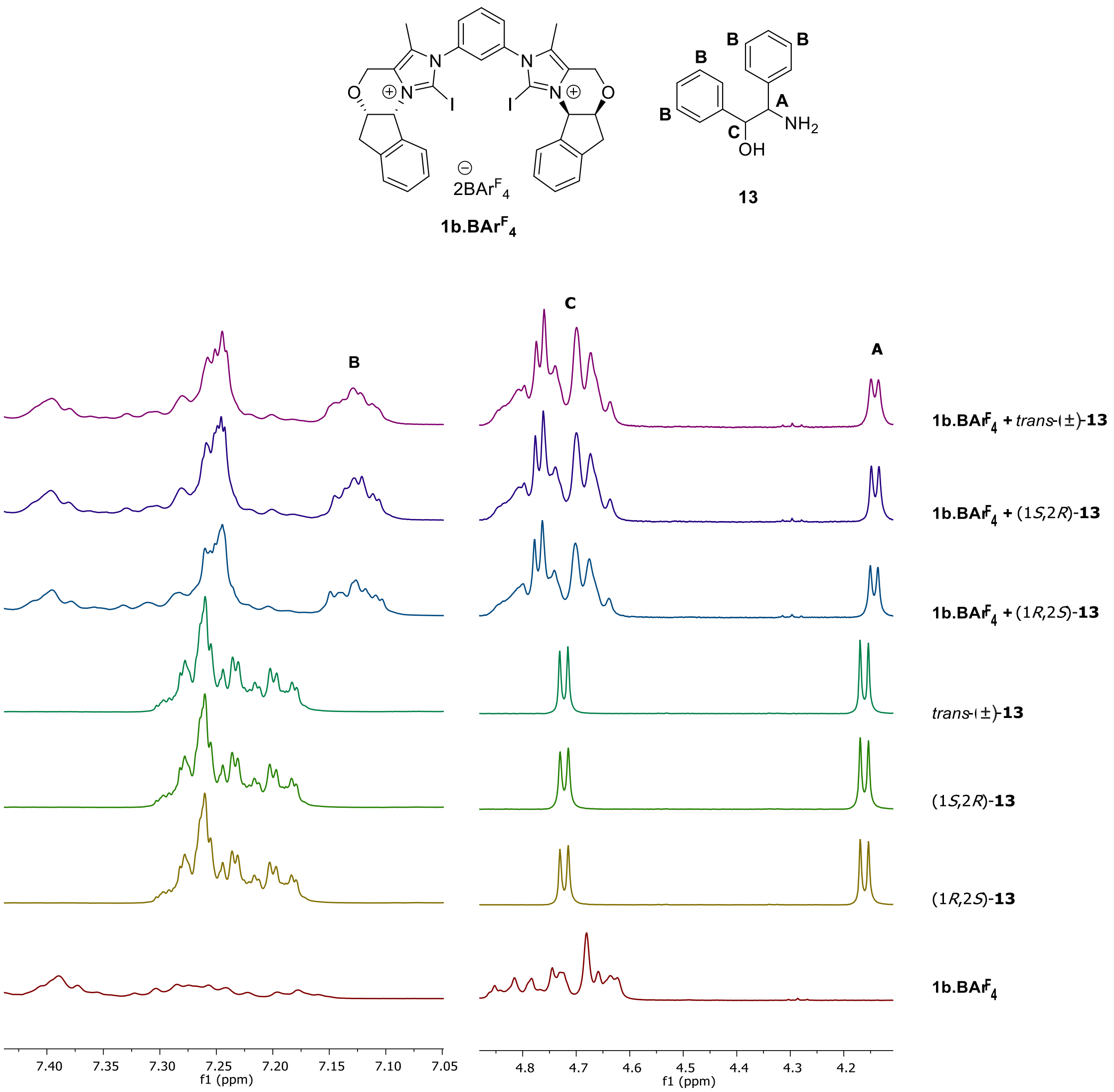

Figure S5. Attempted resolution of *trans*-**13** with **1b.BAr^{F}$_4$** by ^{1}H NMR (CD_2Cl_2, 400 MHz).

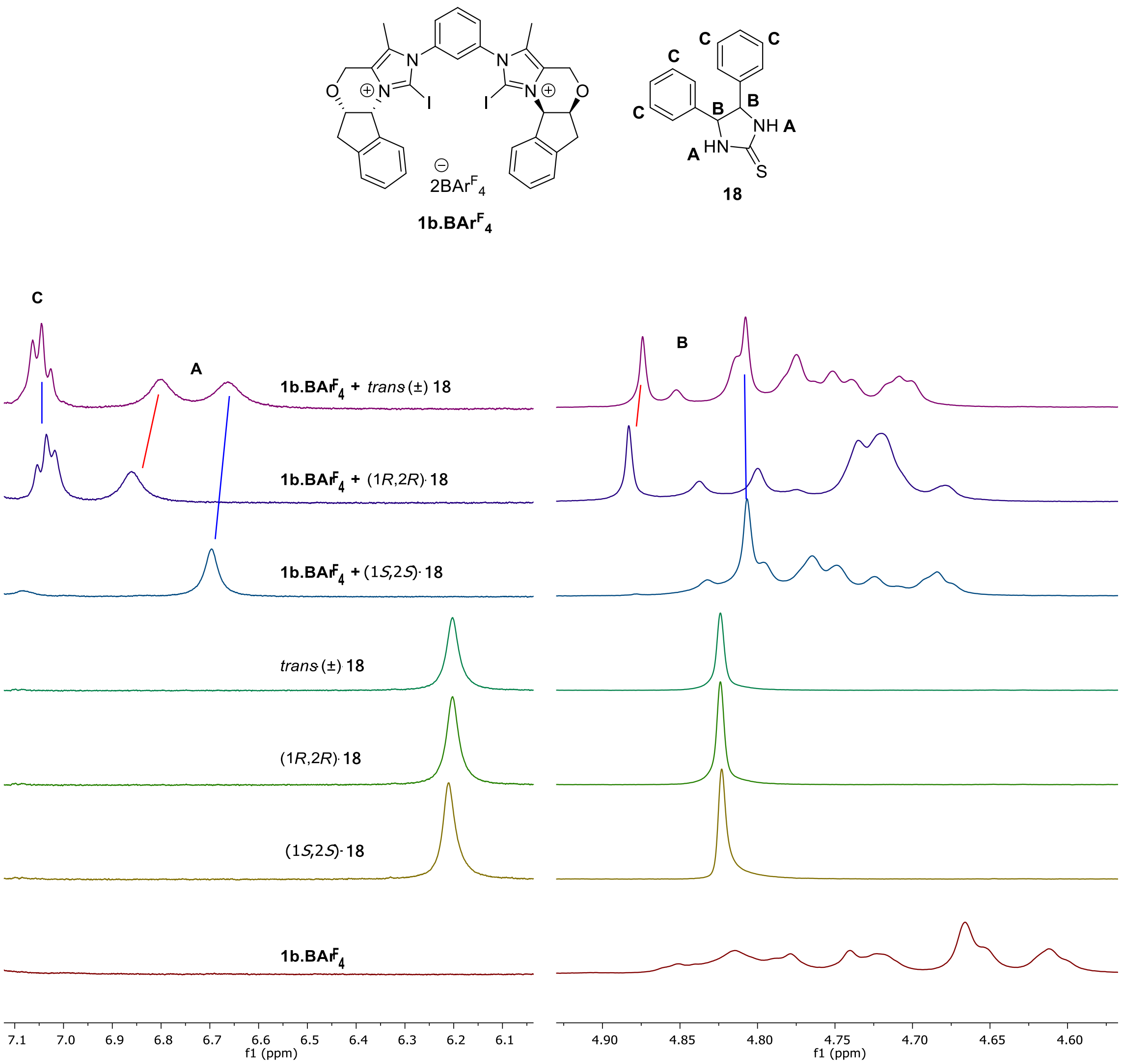

Figure S6. Resolution of *trans*-**18** with **1b.BAr$^{F}_{4}$** by ^{1}H NMR (CD_2Cl_2, 400 MHz).

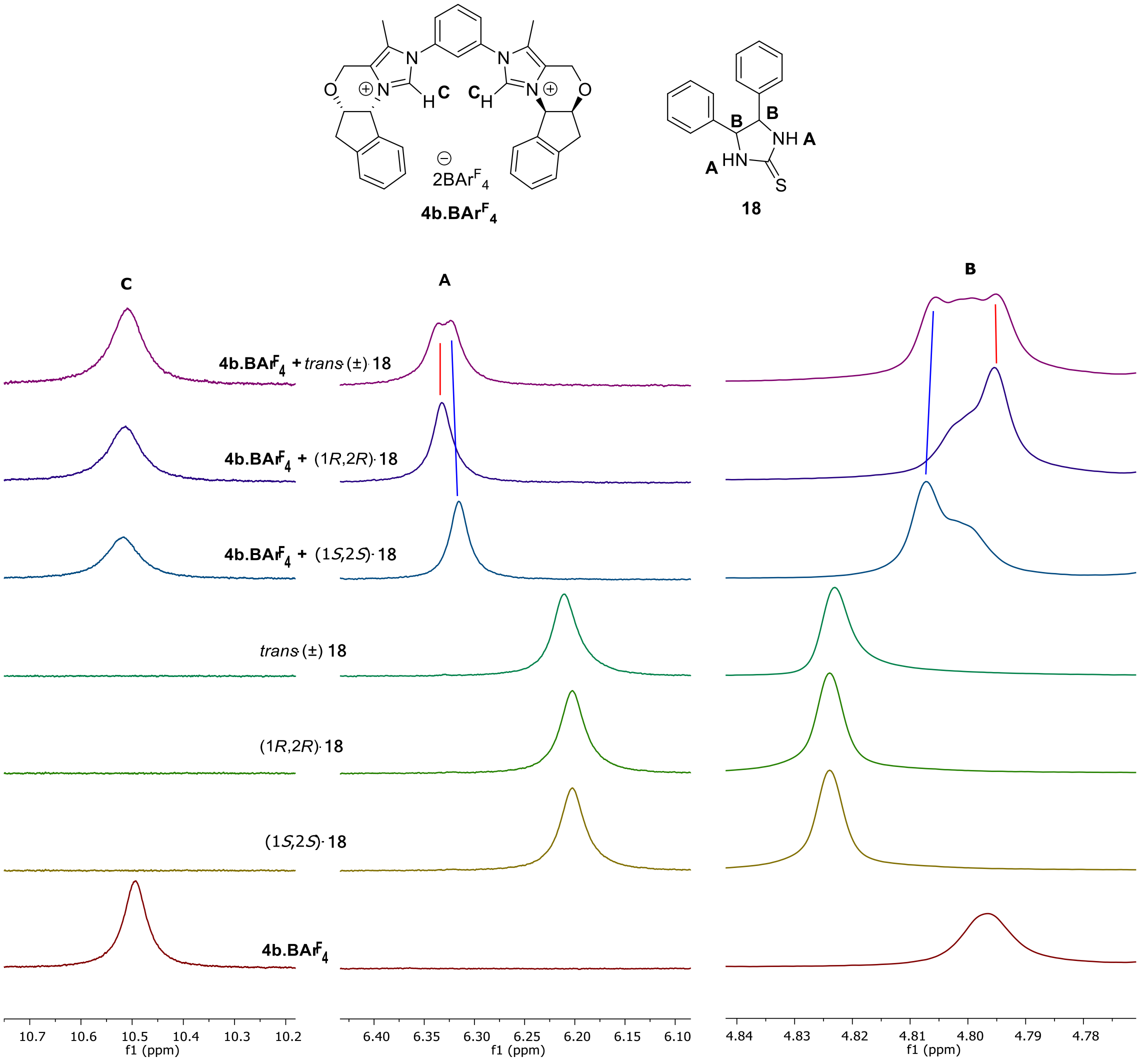

Figure S7. Attempted resolution of *trans*-**18** with **4b.BAr^F$_4$** by ^{1}H NMR (CD_2Cl_2, 400 MHz).

3.2. Titration experiments:

3.2.1. NMR titrations:

General Procedure: For pipetting the samples Hamilton®-syringes were used. All experiments were conducted at ambient temperature (°C) and in Norell® 502 NMR-Tubes. 12.53 mg (5 µmol) of the Host (XB-Donor) were dissolved in 500 µl of CD_2Cl_2. Stock-solutions of the Guest (XB-Acceptor) were prepared in the concentrations of 0.1, 0.2 or 1 M in CD_2Cl_2. For every measured point a single NMR-tube was charged with 500 µl of the guest solution, and the corresponding amount of the host solution. The ^{1}H NMR-spectra were measured with a Bruker DPX-250. In addition to the determination of shits in the NMR signals, the host to guest ratio was checked by integration of the signals and corrected if necessary and the shift of signal of proton (m and n) of

the XB donor was noted. The measured shifts (δ ppm) were plotted against the guest-equivalents and the resulting curve was fitted[10] and the calculations of the binding constants (K) were performed considering the 1:1 stoichiometry.

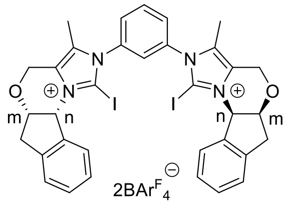

(-)-**1b.BArF4**

Guest	(1*S*,2*S*)-(−)-**11**	(1*R*,2*R*)-(+)-**11**	(1*S*,2*R*)-(+)-**13**	(1*R*,2*S*)-(-)-**13**	(1*S*,2*S*)-(−)-**18**	(1*R*,2*R*)-(−)-**18**
K [M^{-1}]	211±9	349±15	16±4	20±3	2.08×10^{3}±170	2.61×10^{3}±130

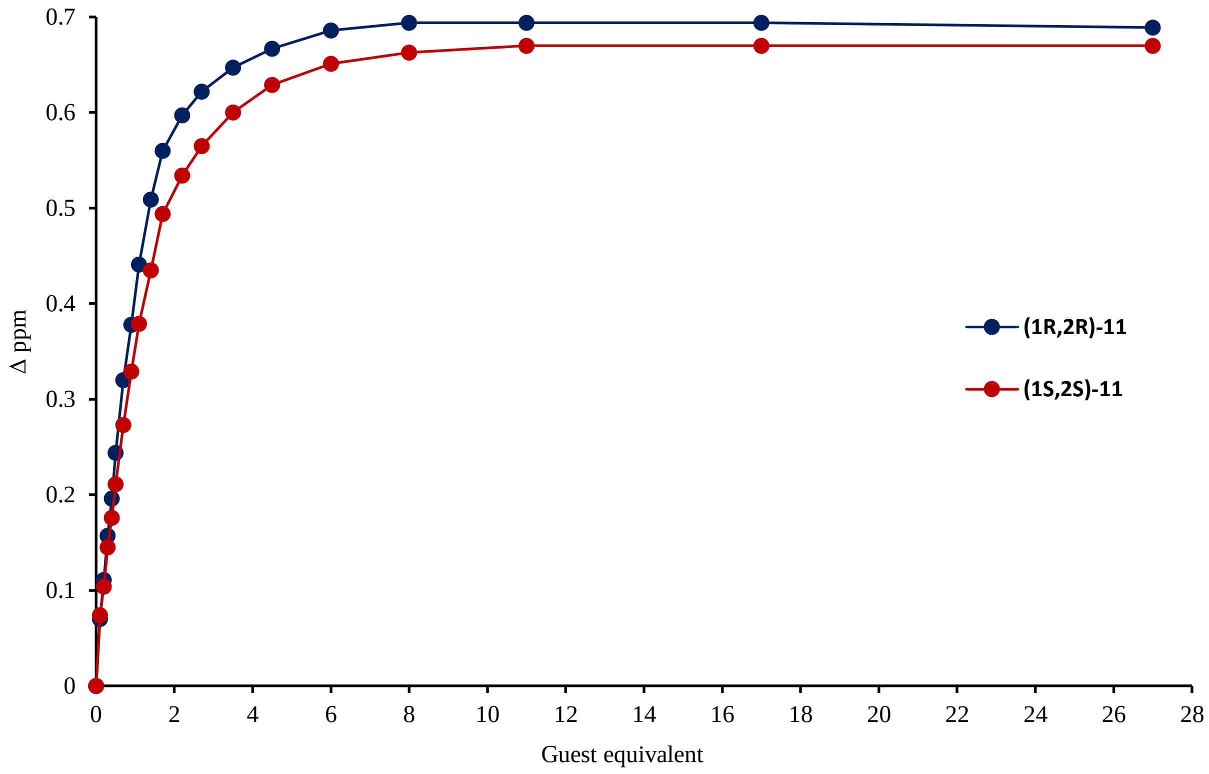

Figure S8. Titration of **1b.BAr$^{F}_{4}$** with **11**

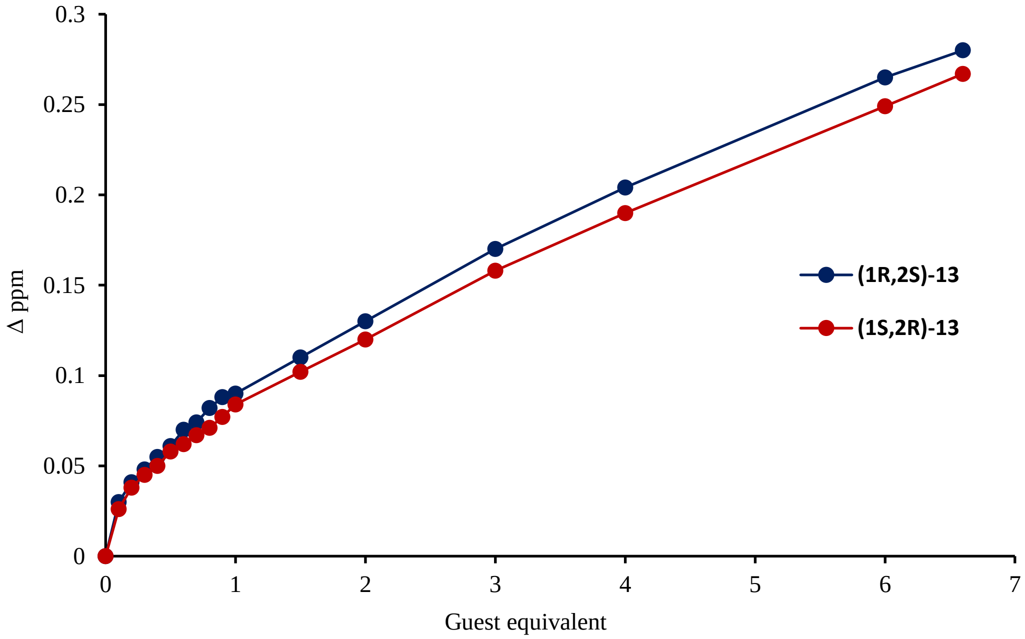

Figure S9. Titration of **1b.BAr^F$_4$** with **13**.

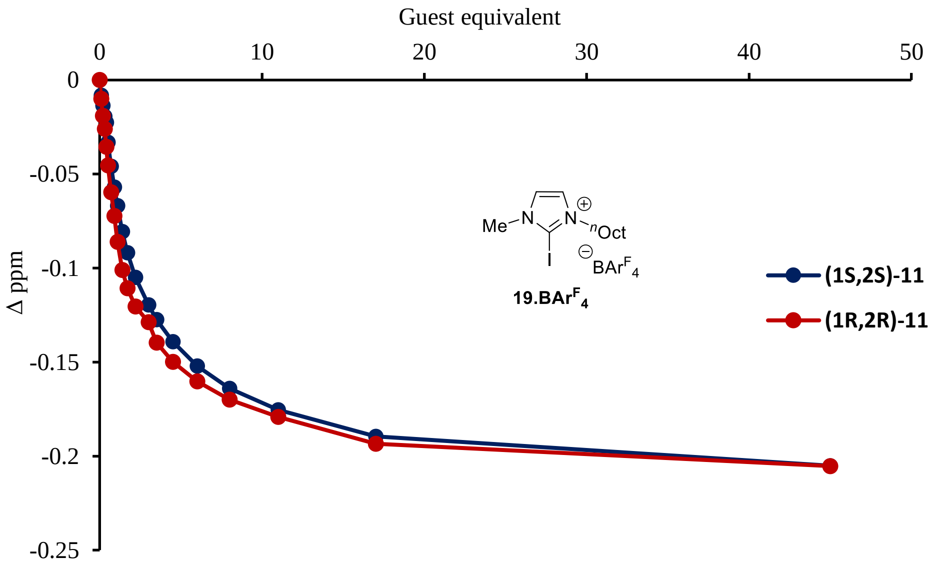

Figure S10. Titration of **19.BAr^F$_4$** with **11** (K = 84±3).

3.2.2. ITC titrations

ITC experiments were carried out at 26 °C in DCM on a VP-ITC (*GE Healthcare*) using a reference power of 20 μcal/s, a filter period of 1 s, a stirrer speed of 329 rpm. A 20 mM solution of XB acceptor in DCM was added to the 1 mM solution of XB donor (taken in cell) using syringe

with the injection volume of 8 μL over 16 s, and a time spacing of 180 s between injections. Evaluation of the obtained data sets was performed with *Origin 7*.

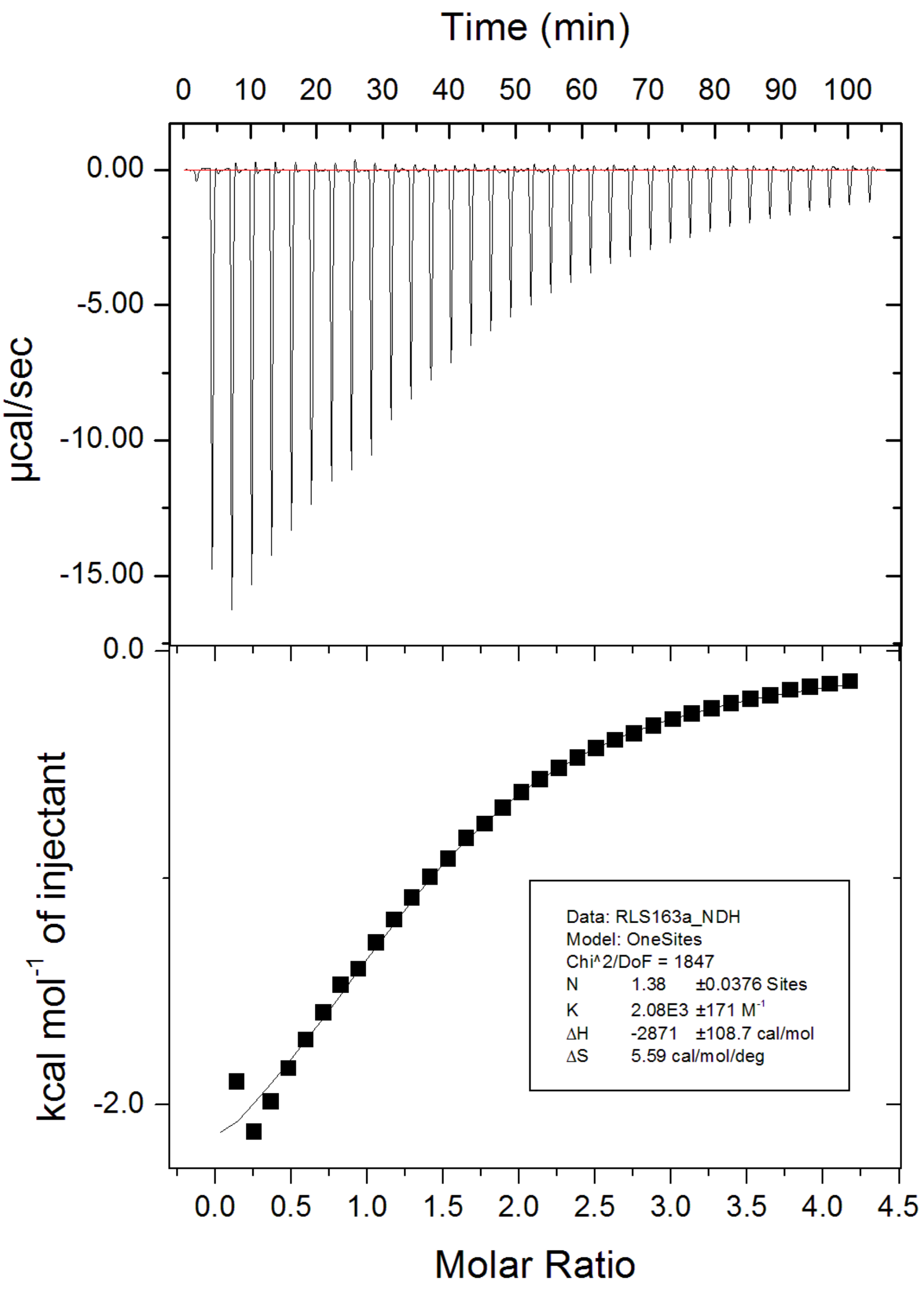

Figure S11. ITC titration of **1b.BAr^{F}** (1 mM) with (1*S*,2*S*)-**18** (20 mM) in DCM.

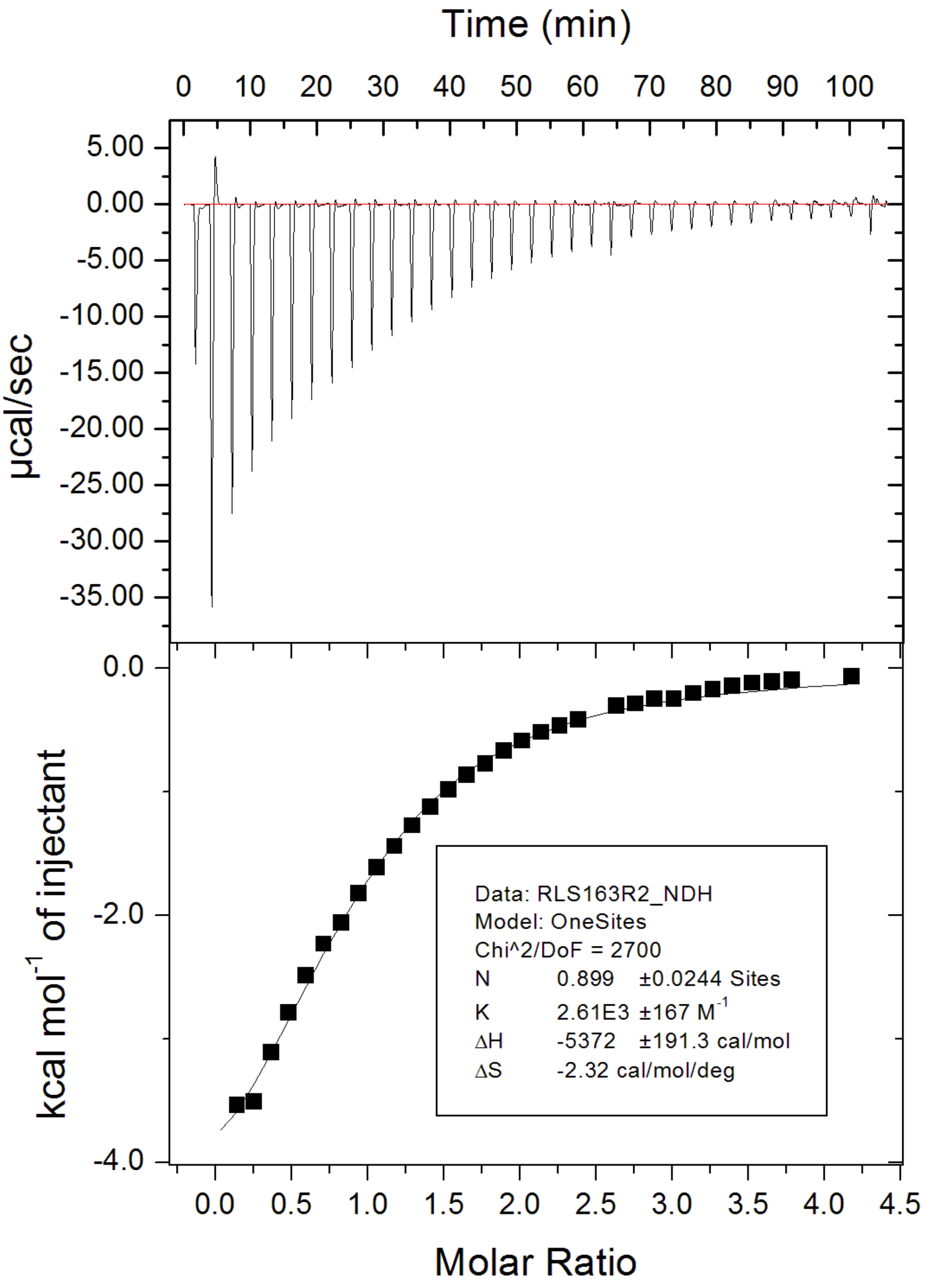

Figure S12. ITC titration of **1b.BAr*F*** (1 mM) with (1*R*,2*R*)-**18** (20 mM) in DCM.

4. Catalysis of Mukaiyama-aldol reaction:

4.1. Kinetics experiments:

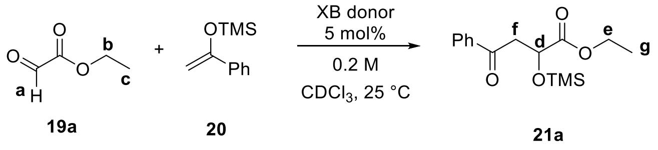

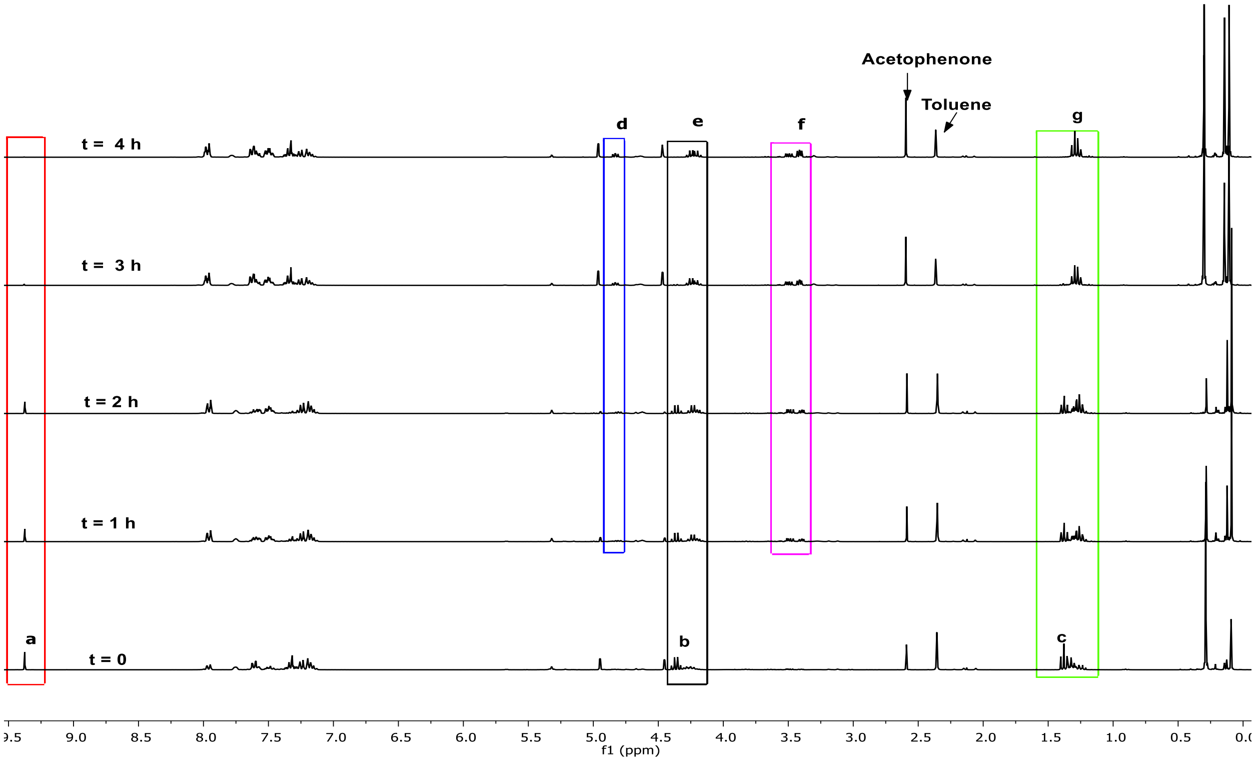

Figure S13. Reaction monitoring for stoichiometry and product peak confirmation.

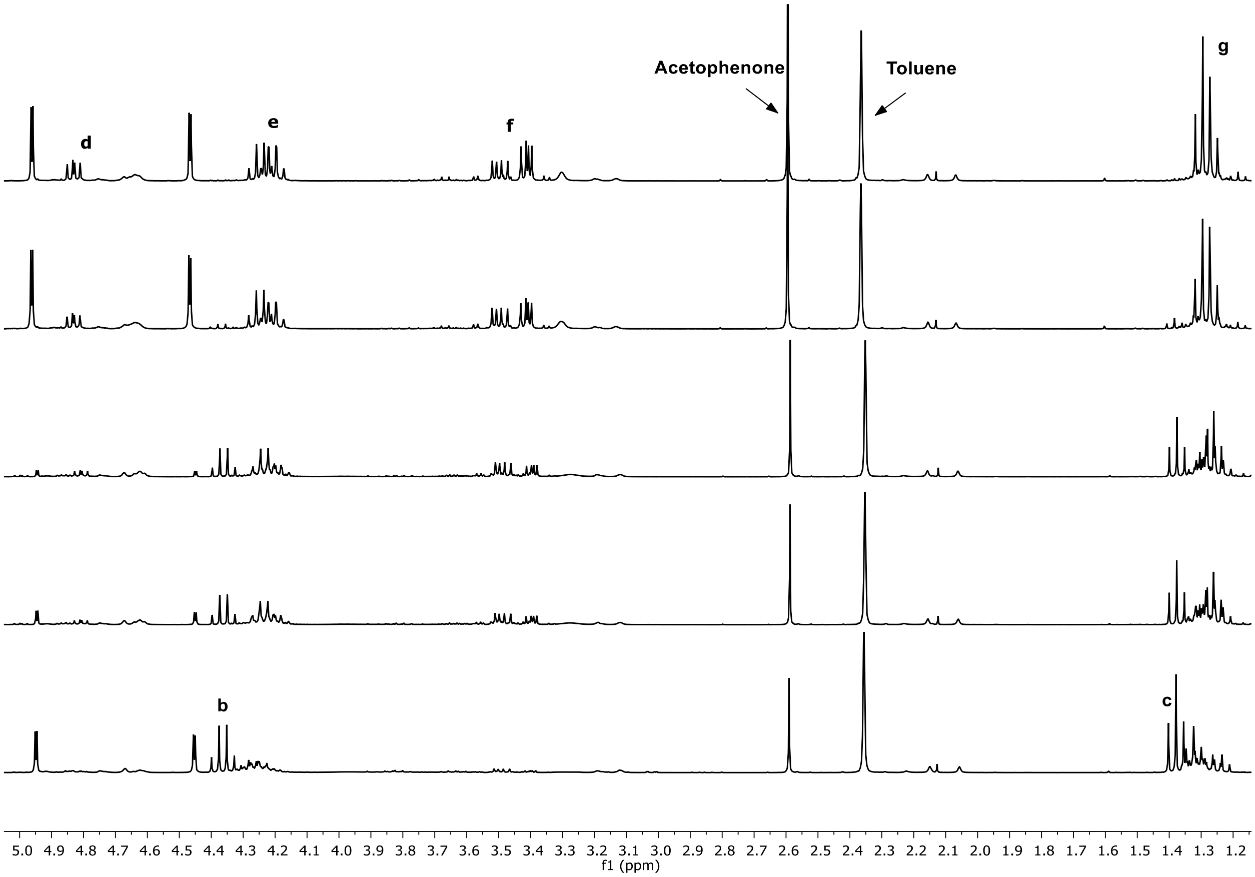

Figure S14. Zoomed spectra of reaction monitoring for stoichiometry and product peak confirmation.

In NMR tube, **19a** (20 µl, 50% solution in toluene, 0.1 mmol, 1 equiv.), **20** (19.2 mg, 0.1 mmol, 1 equiv.) and **1b.BAr^F$_4$ (**12.53 mg, 0.05 mmol, 5 mol%) were dissolved in CD_2Cl_2 (0.5 mL) under argon atmosphere and the progress of reaction was monitored by ^{1}H NMR at 25 °C. As there was little decomposition of TMS-enolate to acetophenone during the reaction, after 2 h additional 1 equiv. of TMS-enolate was added. Presence of unreacted TMS-enolate indicated us that 1.5 equiv. TMS-enolate is sufficient. Also it was observed that the yield are almost identical in CD_2Cl_2 and $CDCl_3$ as the solvent. Therefore for the kinetic experiments, 1.5 equiv. TMS-enolate and $CDCl_3$ as solvent was used.

Control experiments to rule out NHC formation:

Under argon atmosphere, a NMR tube was charged with **4b.BAr^F$_4$** (11.27 mg, 0.005 mmol) and sodium *tert*-butoxide (1.9 mg, 0.02 mmol). Anhydrous CD_2Cl_2 (0.5 mL) was added to it at 0 °C and the progress of reaction was monitored by ^{1}H NMR till the disappearance of imidazolium protons (20 min at room temperature). To this homogenous solution of bis(NHC), **19a** (20 µl, 50% solution in toluene, 0.1 mmol, 1 equiv.) and **20** (19.2 mg, 0.1 mmol, 1 equiv.) was added and the reaction was monitored by ^{1}H NMR. Although aldehyde was converted to some unknown product, the expected aldol product was not formed.

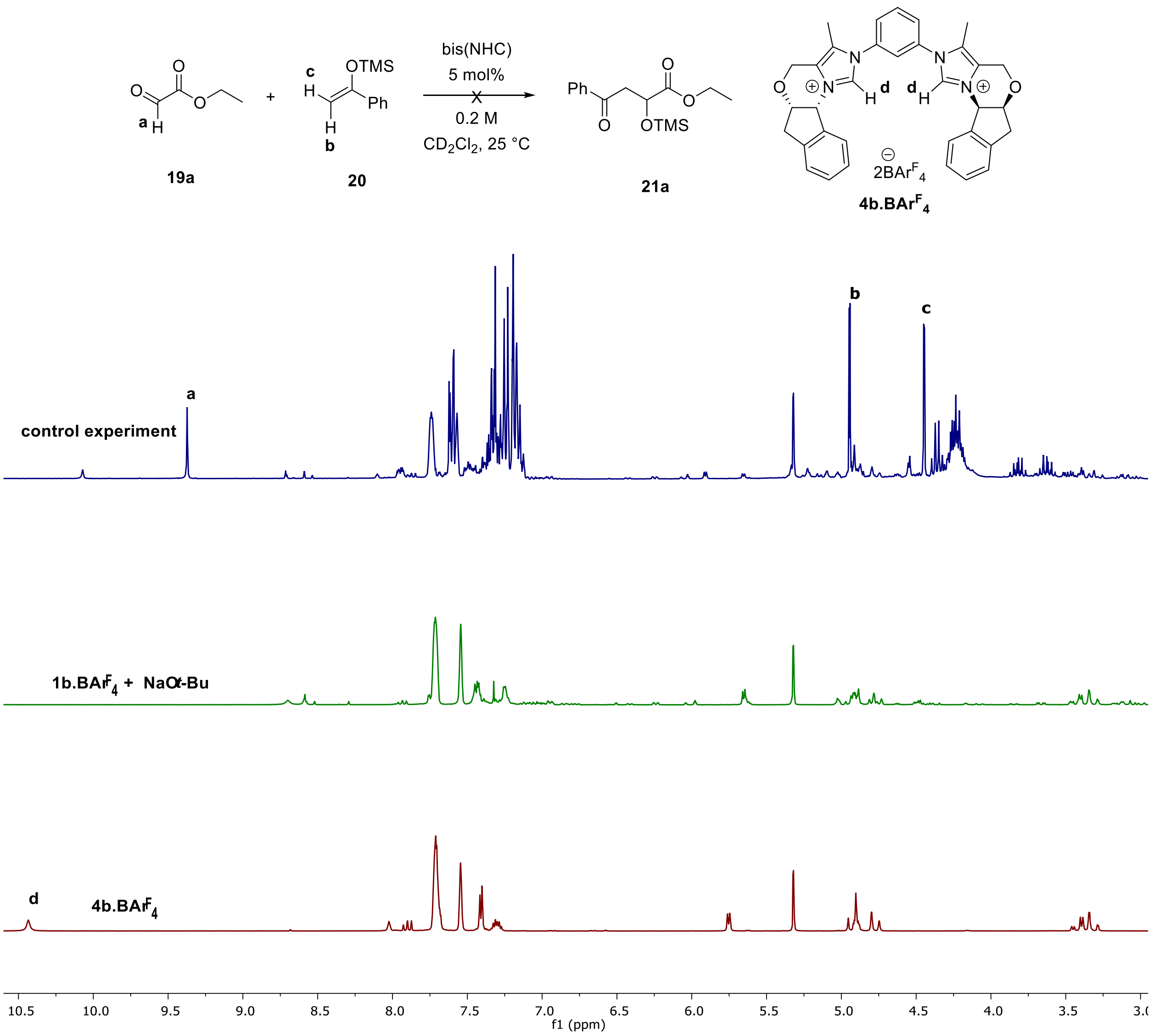

Figure S15. Control experiment to rule out formation of NHC.

4.2. Asymmetric catalysis of Mukaiyama aldol reaction:

A 10 mL Schlenk flask was charged with aldehyde (0.1 mmol), catalyst (0.05 mmol, 5 mol%) and anhydrous DCM (2 mL) under argon atmosphere. The solution was cooled to the respective temperature and the TMS-enolate (0.2 mmol) was added to it with the syringe and the reaction was monitored by TLC. After the maximum conversion of aldehyde to the product, reaction mass was poured in water (5 mL). It was extracted with DCM (2 × 10 mL) and the combined organic extracts were dried over anhydrous $MgSO_4$. Solvent was evaporated and the crude product was purified by column chromatography using ethyl acetate/pentane as the eluent. *ee* of the product OTMS-**21** was analyzed on ReproSil Chiral-AM, 10 µm (250 × 4.6 mm) column while that of OH-**21** was measured

on ReproSil Chiral-JM, 5 μm (250 × 4.6 mm) column using isopropanol/hexane as the solvent at 1 mL/min flow rate.

NMR spectra of isolated Mukaiyama aldol products

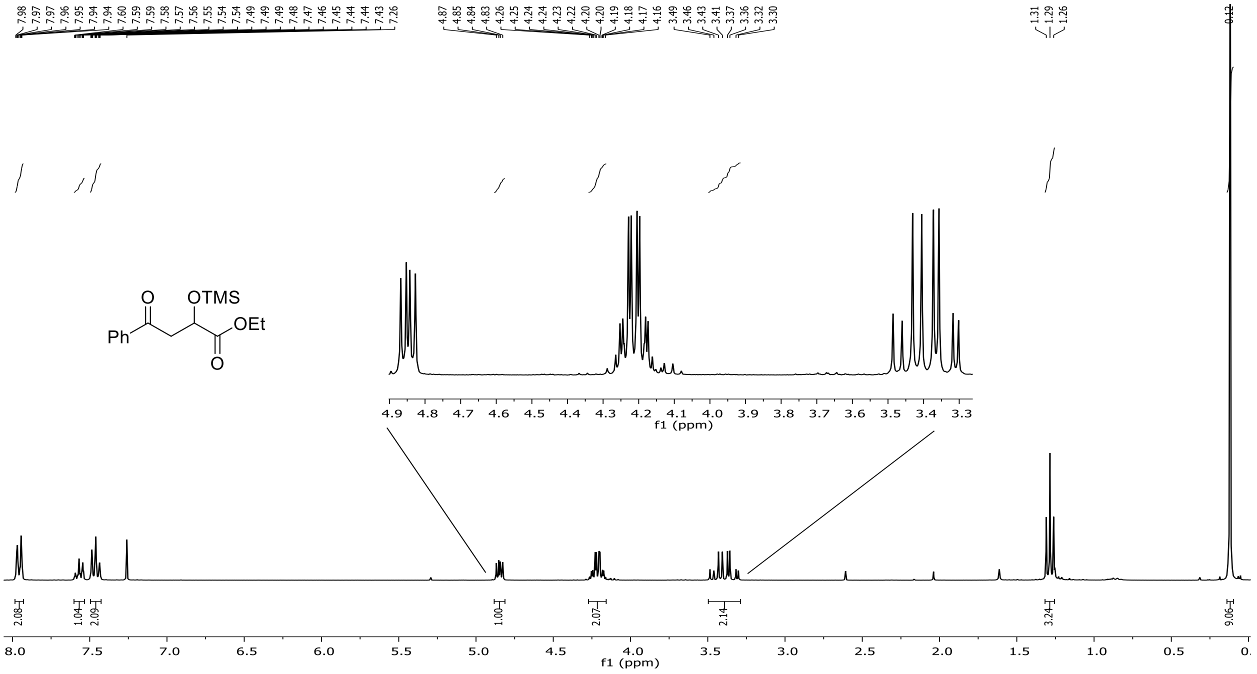

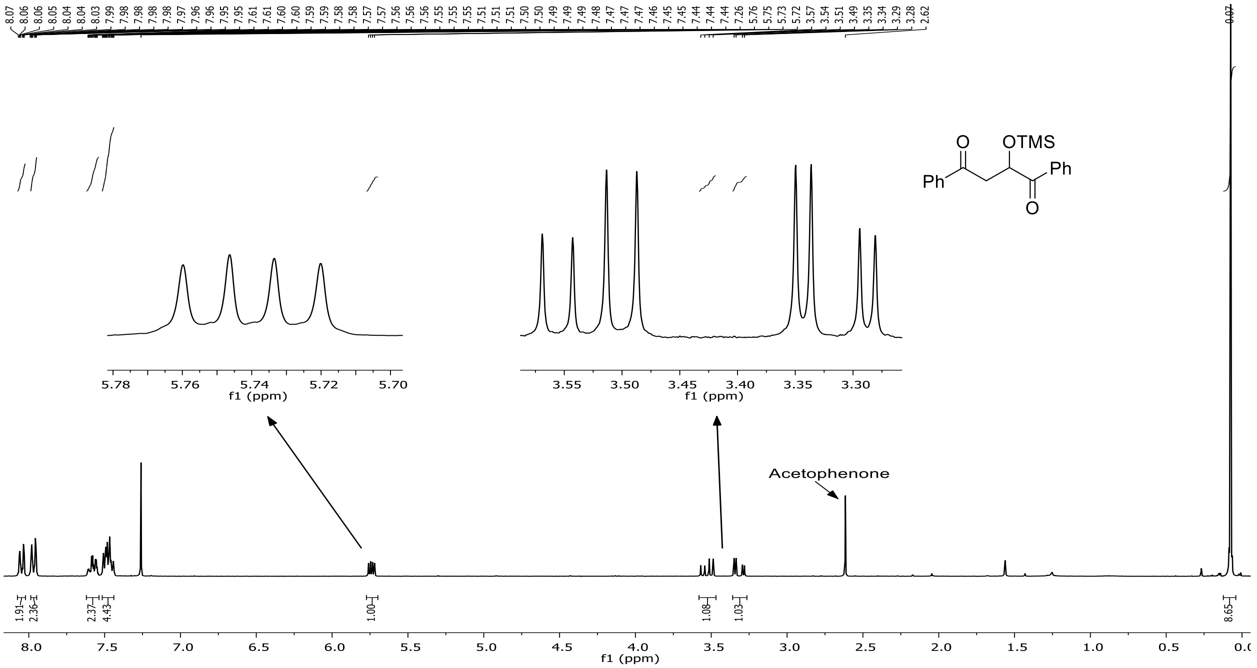

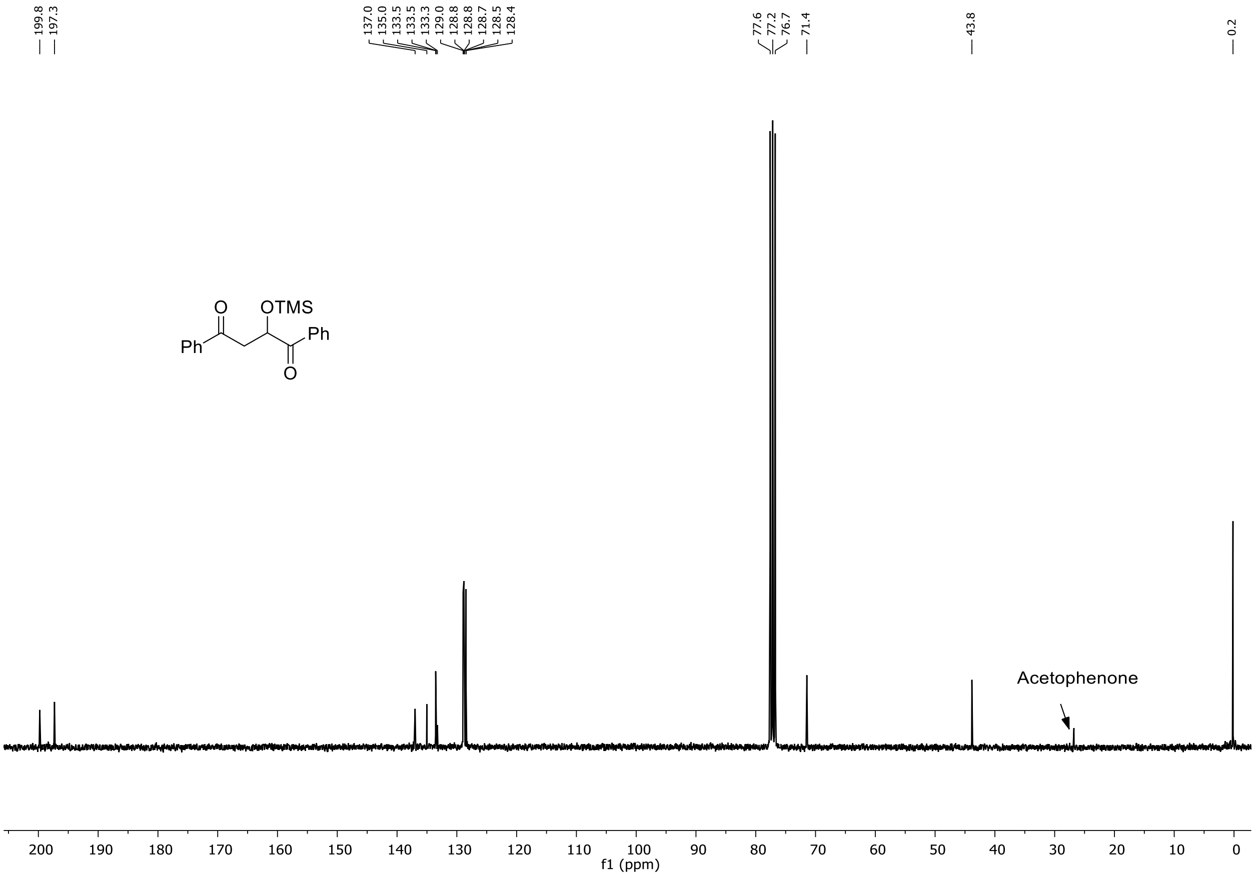
199.8
197.3
137.0
135.0
133.5
133.5
133.3
129.0
128.8
128.8
128.7
128.5
128.4
77.6
77.2
76.7
71.4
43.8
0.2
O
OTMS
Ph
Ph
O
Acetophenone
200 190 180 170 160 150 140 130 120 110 100 90 80 70 60 50 40 30 20 10 0
f1 (ppm)

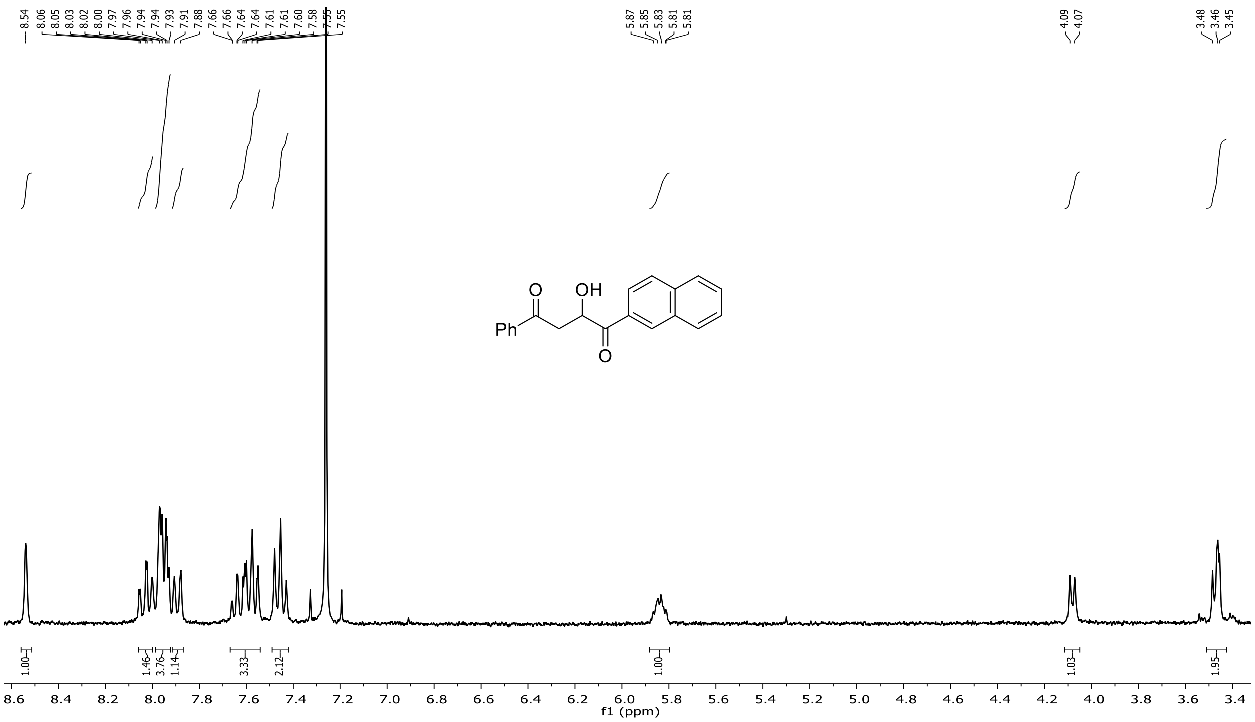
8.54
8.06
8.05
8.03
8.02
8.00
7.97
7.96
7.94
7.94
7.93
7.91
7.88
7.66
7.64
7.64
7.61
7.60
7.58
7.55
7.55
5.87
5.85
5.83
5.81
5.81
4.09
4.07
3.48
3.46
3.45
O
OH
Ph
O
1.00
1.46
3.76
1.14
3.33
2.12
1.00
1.03
1.95
8.6 8.4 8.2 8.0 7.8 7.6 7.4 7.2 7.0 6.8 6.6 6.4 6.2 6.0 5.8 5.6 5.4 5.2 5.0 4.8 4.6 4.4 4.2 4.0 3.8 3.6 3.4
f1 (ppm)

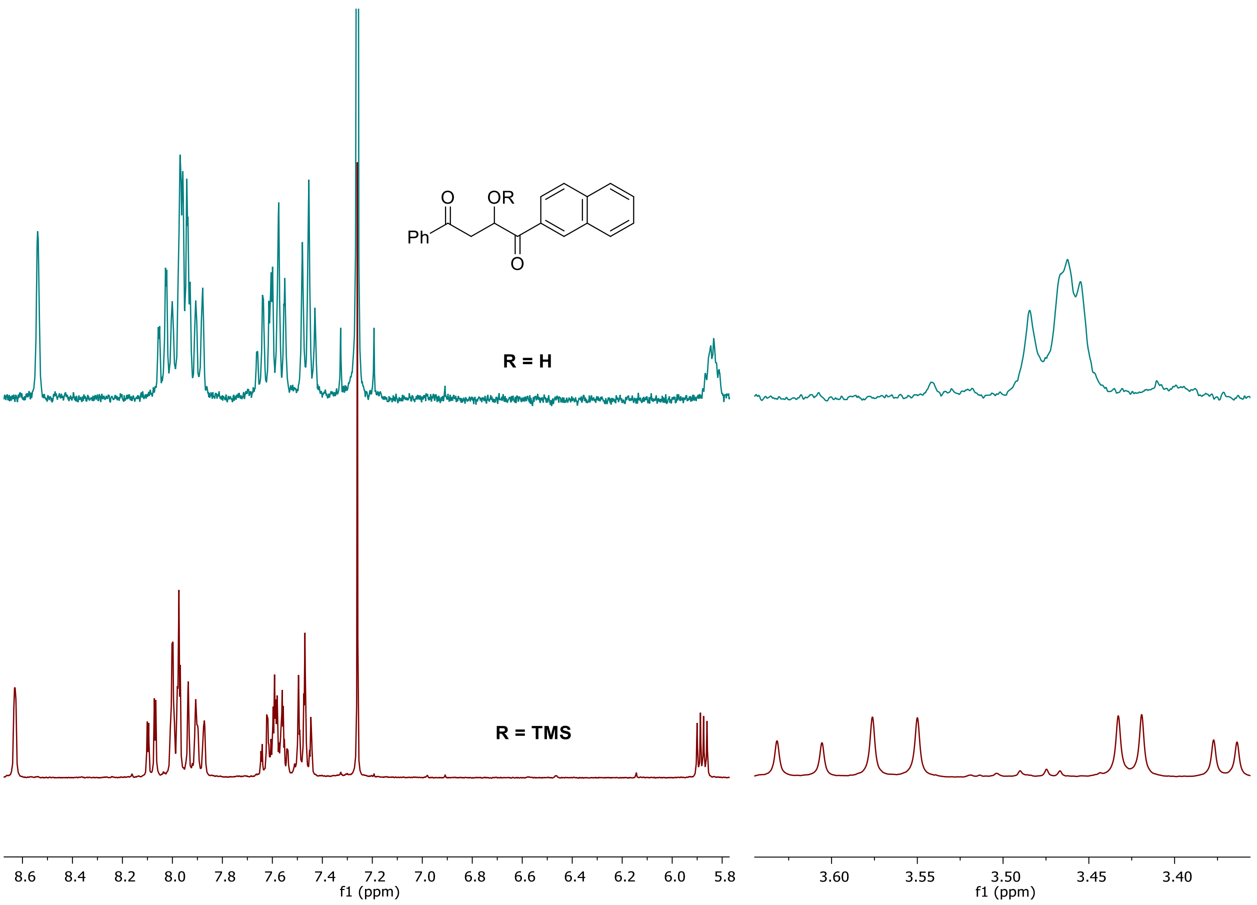
Ph
O
OR
O
R = H
R = TMS
8.6
8.4
8.2
8.0
7.8
7.6
7.4
7.2
7.0
6.8
6.6
6.4
6.2
6.0
5.8
f1 (ppm)
3.60
3.55
3.50
3.45
3.40
f1 (ppm)

Chiral HPLC chromatogram (Table 1, entry 8)

Analyzed on ReproSil Chiral-JM, 5 µm (250 × 4.6 mm) column using isopropanol/hexane (3:97) as the solvent with 1 mL/min flow rate and at 230 nm detector wavelength.

Result Table (Uncal - RLS-169-70-JM_09.12.2019 17_36_33_215000061 - DAD 2.1L: Channel 2)

	Reten. Time [min]	Area [mAU.s]	Height [mAU]	Area [%]
1	51,800	14640,350	185,884	66,3
2	63,867	7444,839	82,186	33,7
	Total	22085,189	268,070	100,0

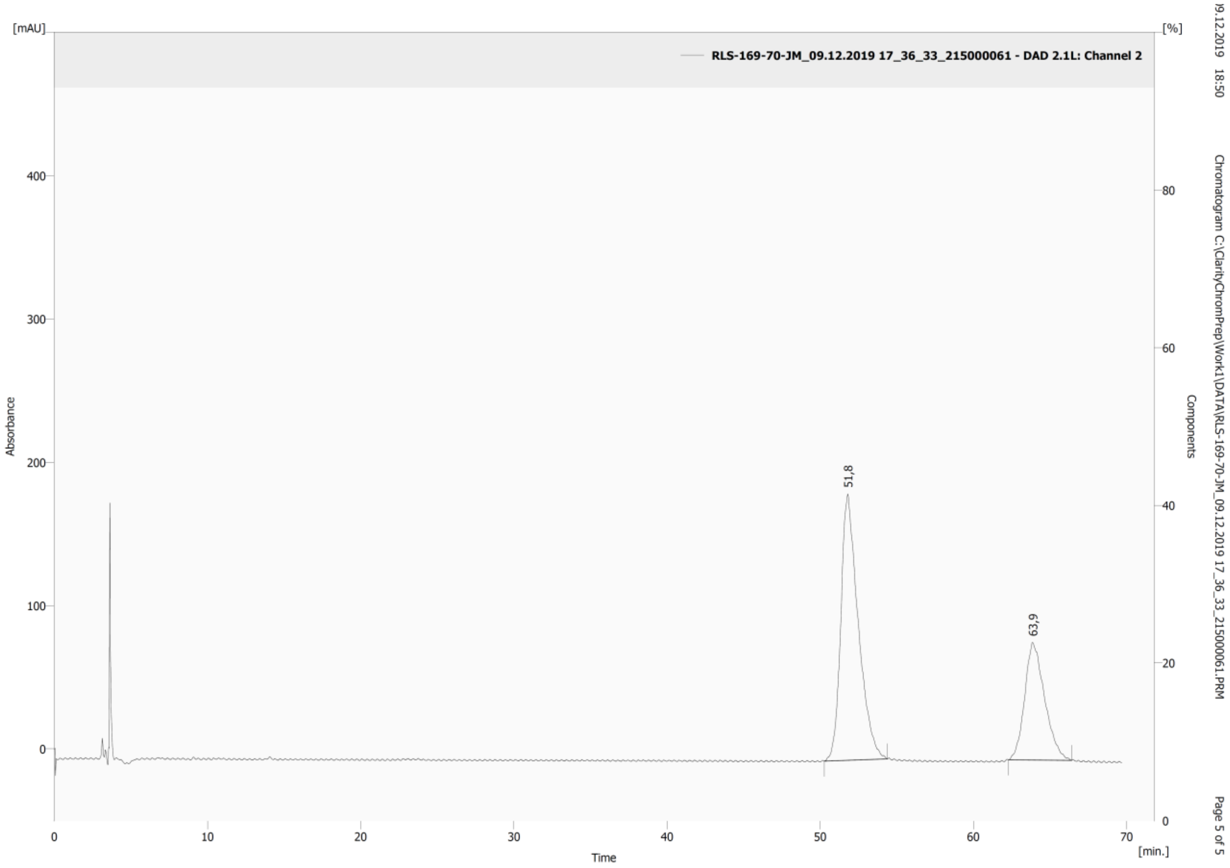

5. NMR spectra of XB synthesis

^{1}H NMR (300 MHz, $CDCl_3$) of **4a.Cl**

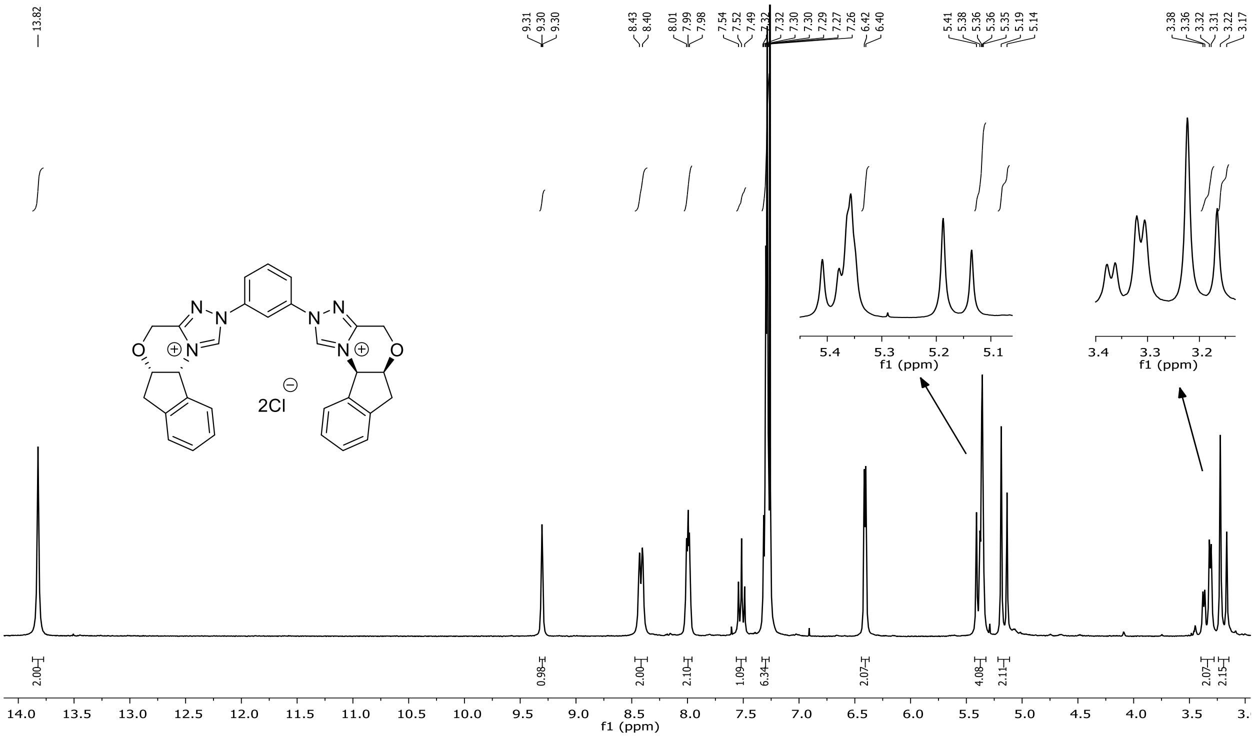

^{13}C NMR (75 MHz, $CDCl_3$) of **4a.Cl**

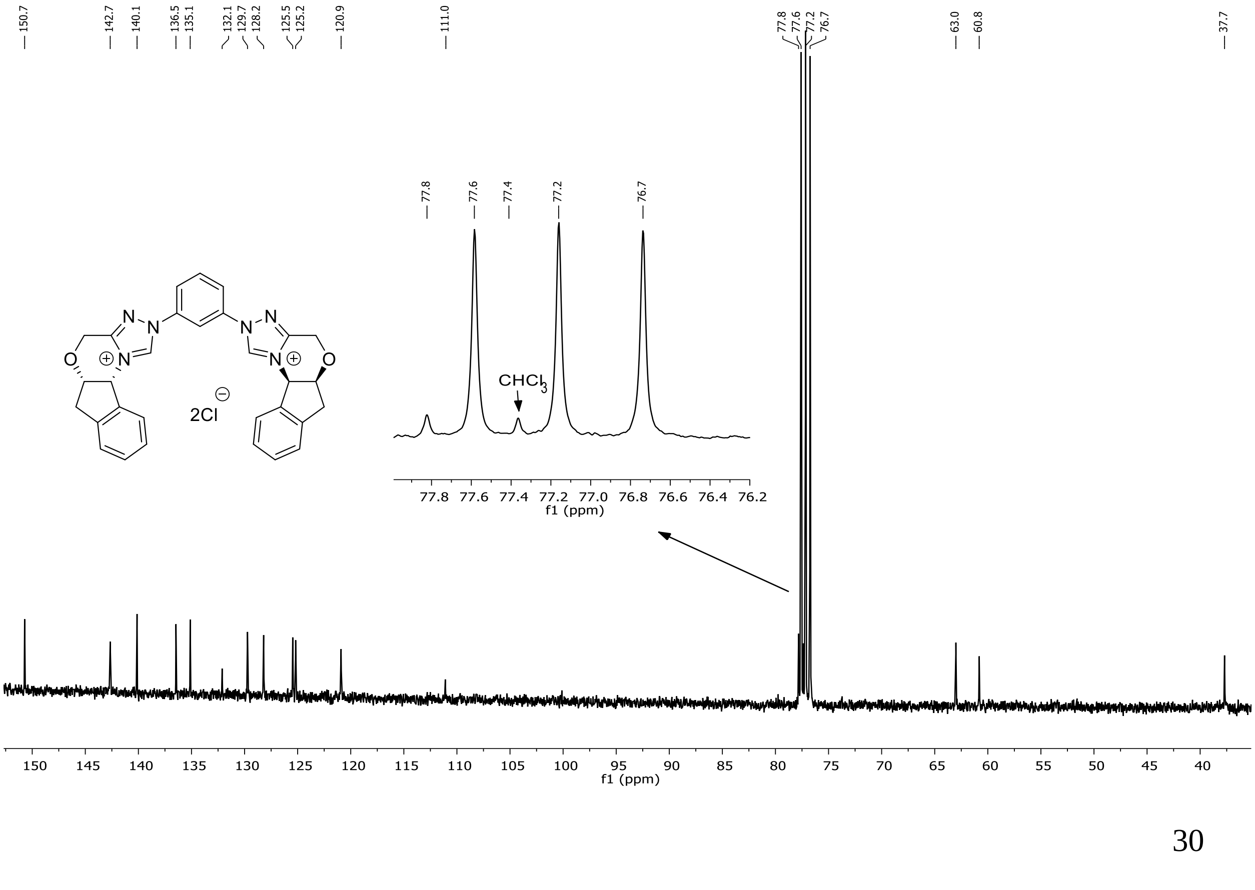

^{1}H NMR (300 MHz, $CDCl_3$) of **5**

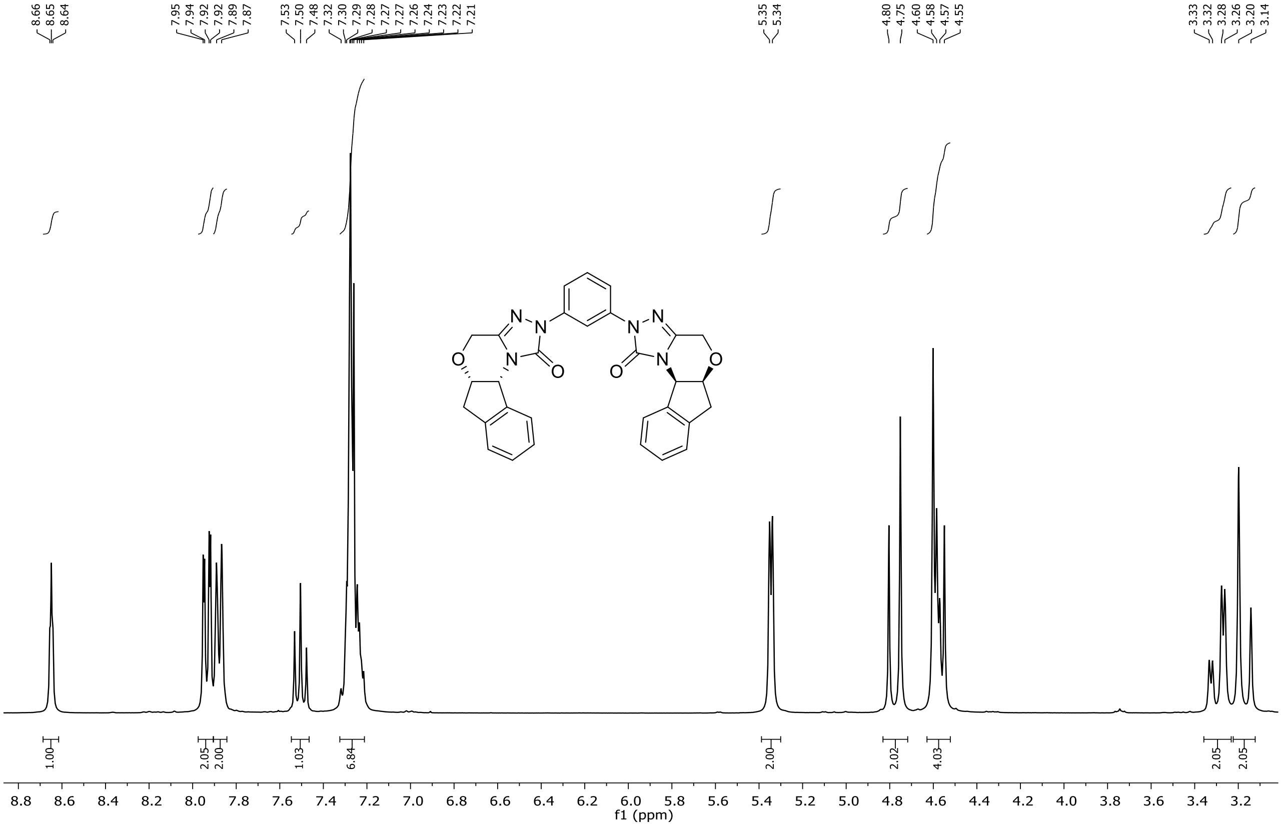

^{13}C NMR (75 MHz, $CDCl_3$) of **5**

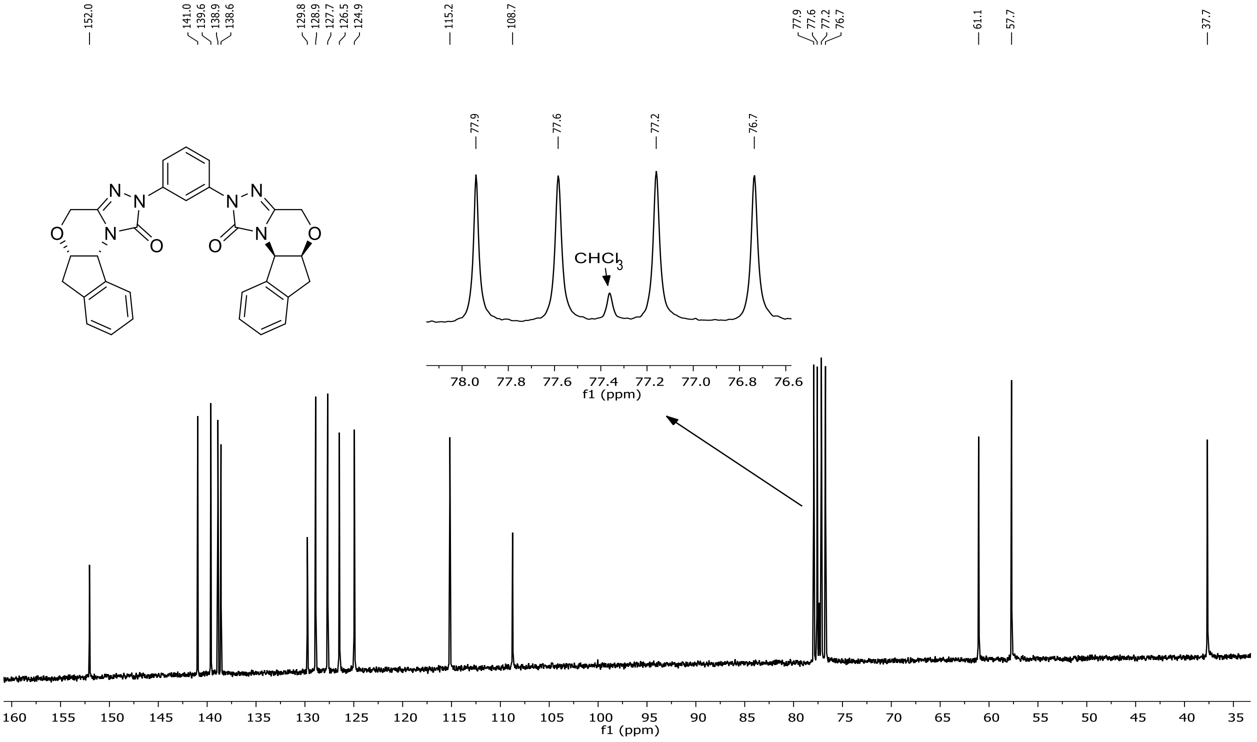

^{1}H NMR (300 MHz, $CDCl_3$) of **4b.Cl**

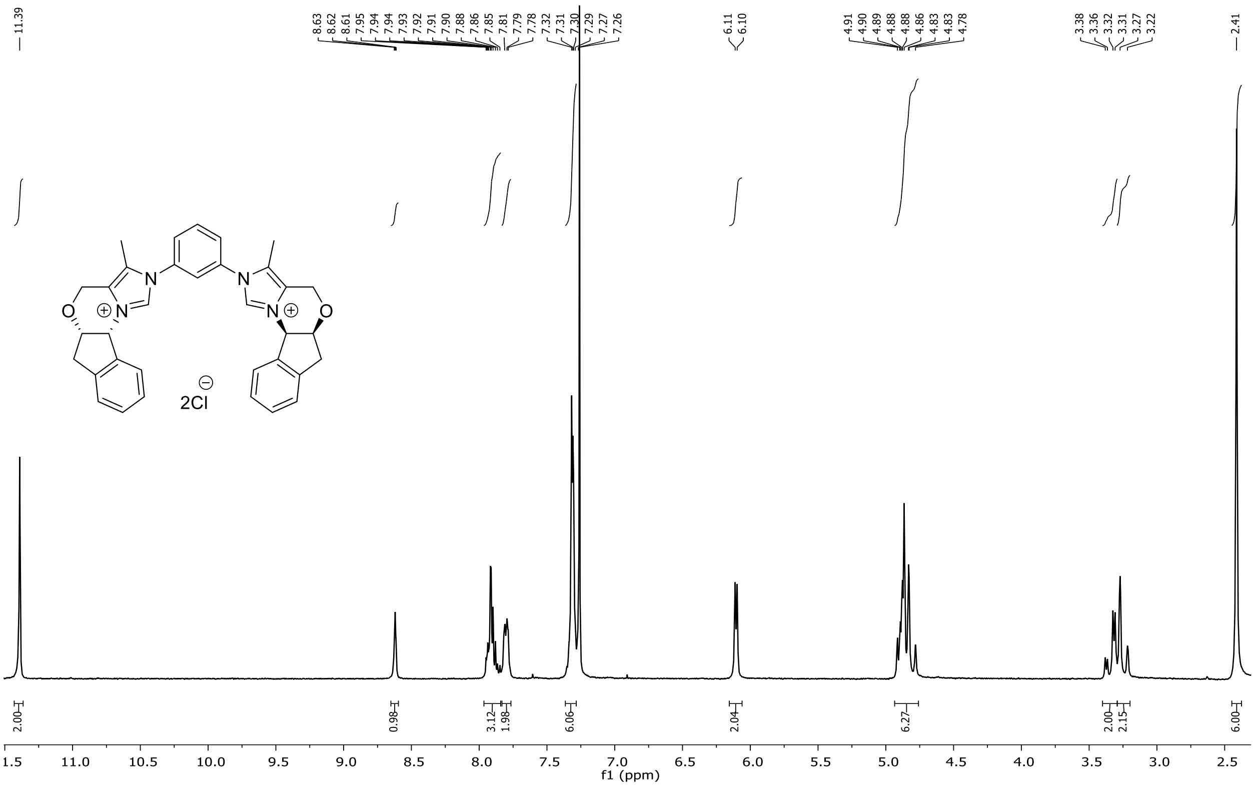

^{13}C NMR (63 MHz, $CDCl_3$) of **4b.Cl**

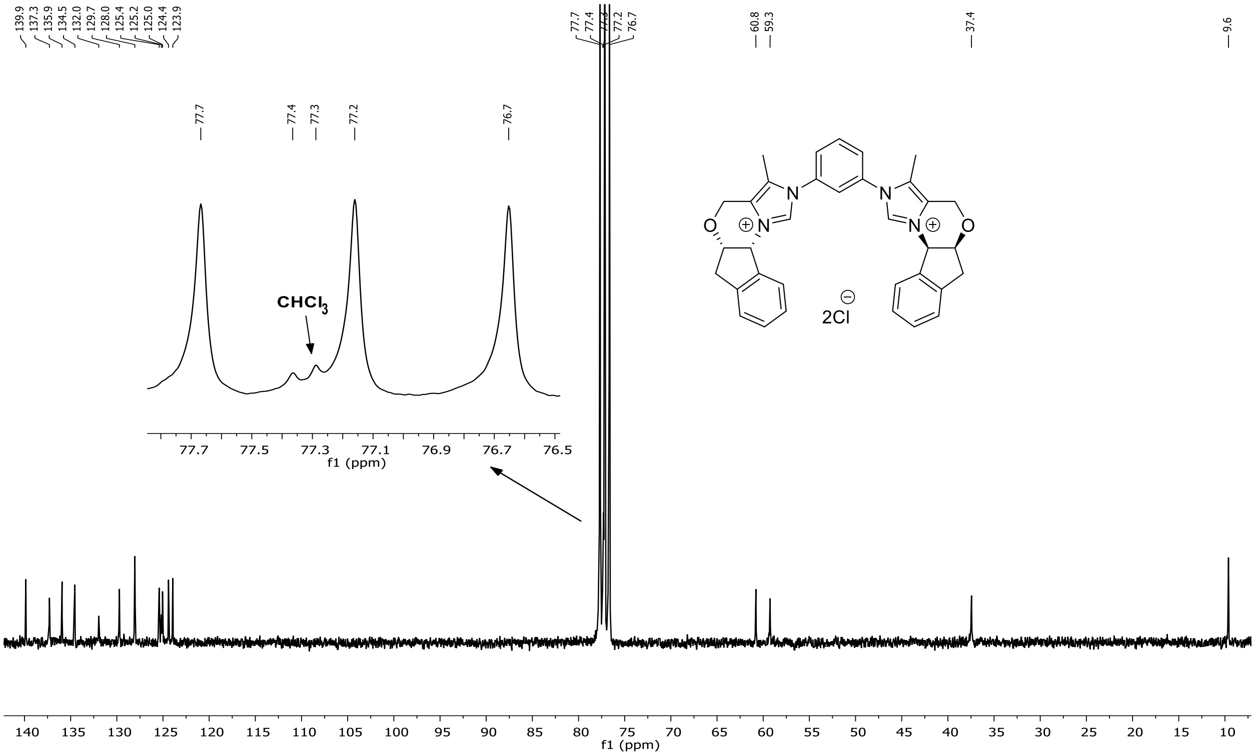

^{1}H NMR (250 MHz, DMSO-*d6*) of **4c.Cl**

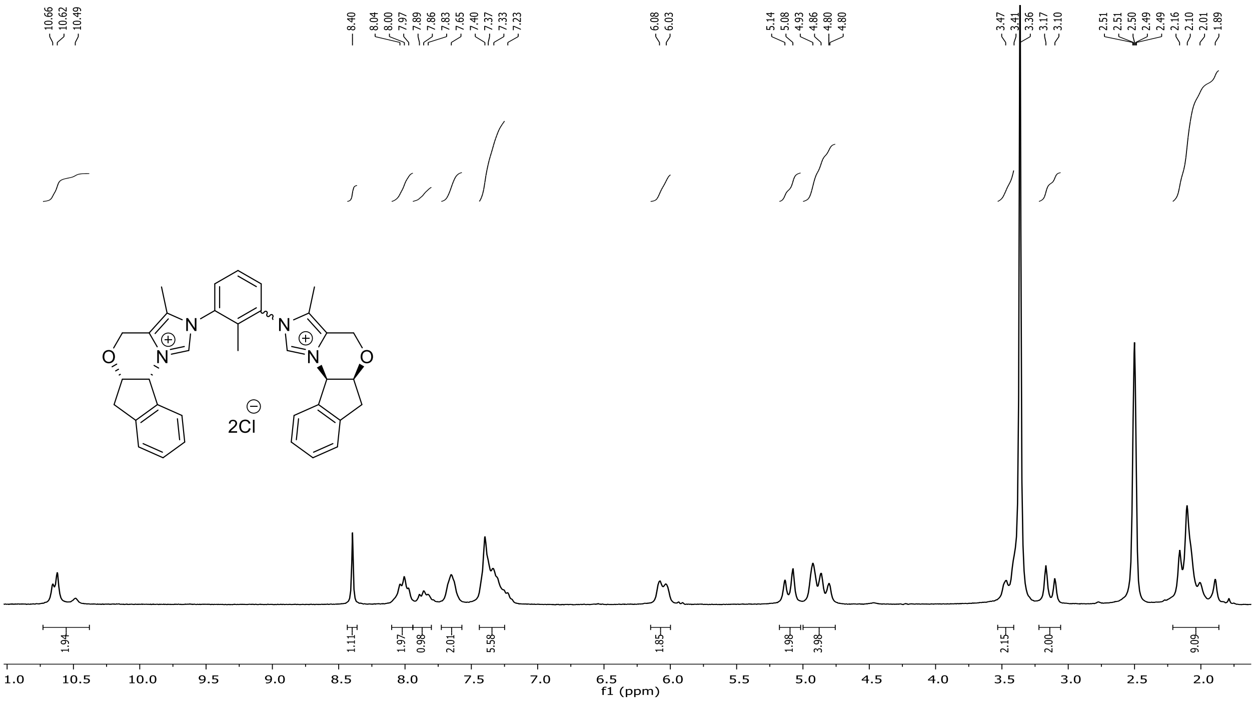

^{13}C NMR (63 MHz, DMSO-*d6*) of **4c.Cl**

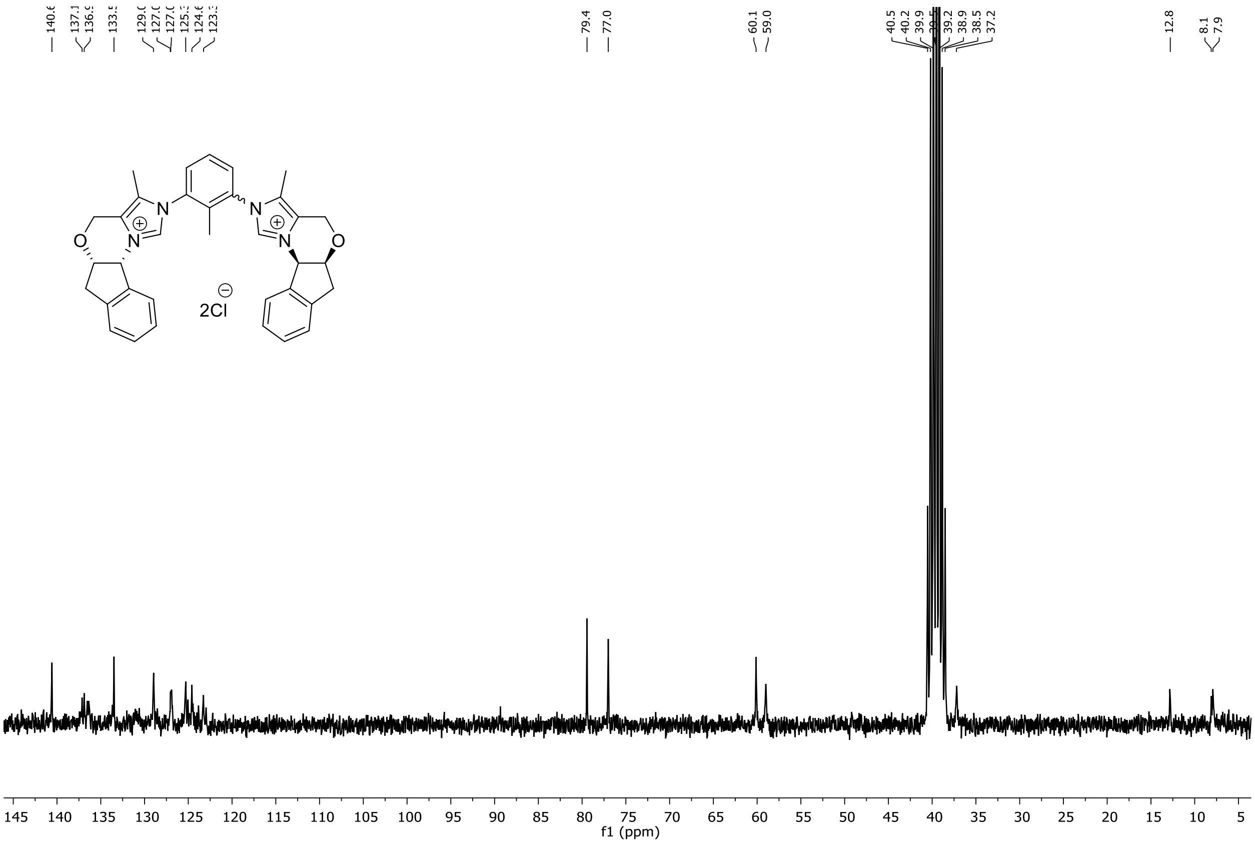

^{1}H NMR (300 MHz, CD_2Cl_2) of **4b.OTf**

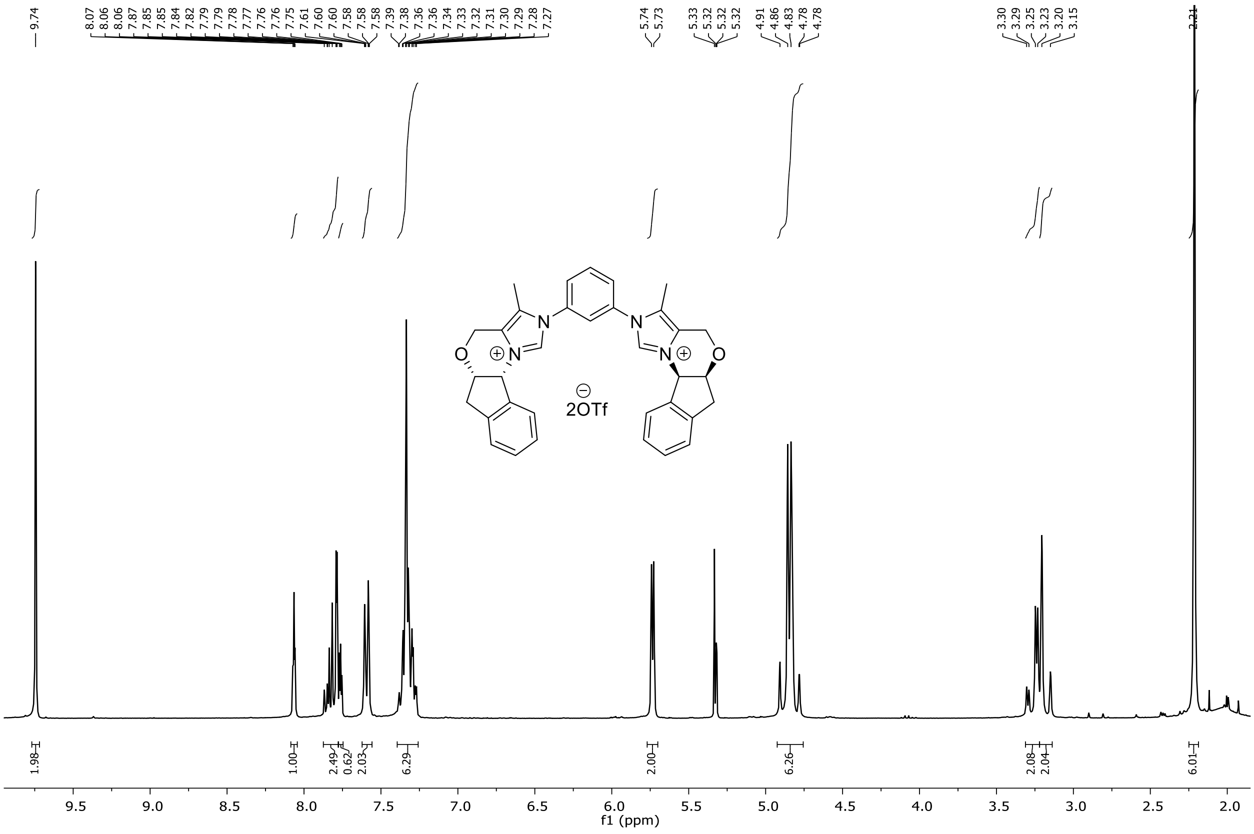

^{13}C NMR (75 MHz, CD_2Cl_2) of **4b.OTf**

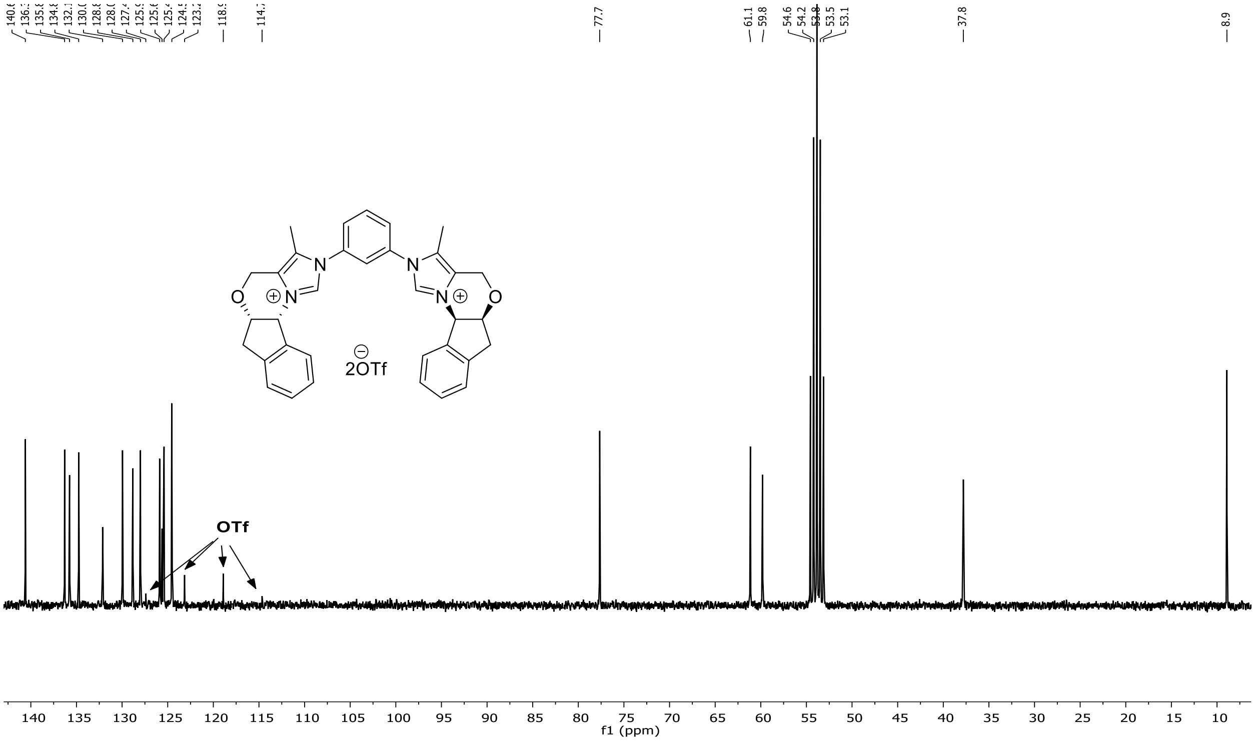

^{19}F NMR (235 MHz, $CDCl_3$) of **4b.OTf**

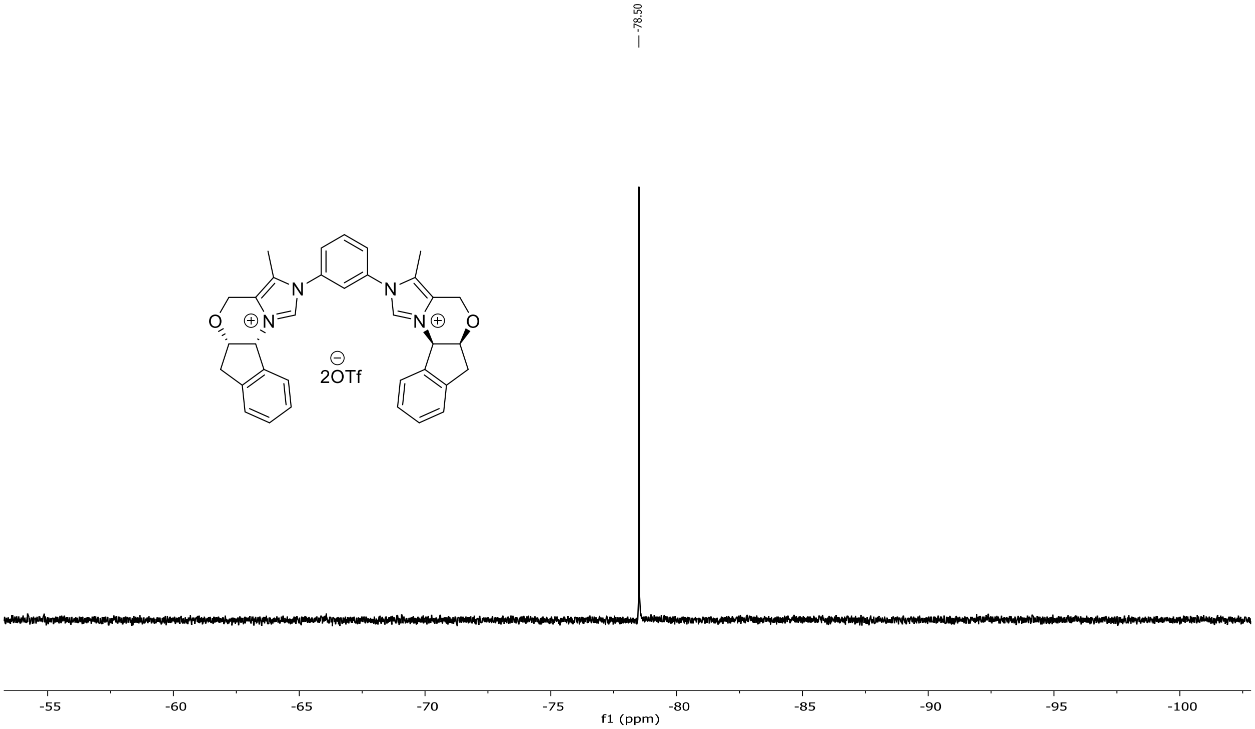

^{1}H NMR (300 MHz, CD_2Cl_2) of **4b.BAr^F$_4$**

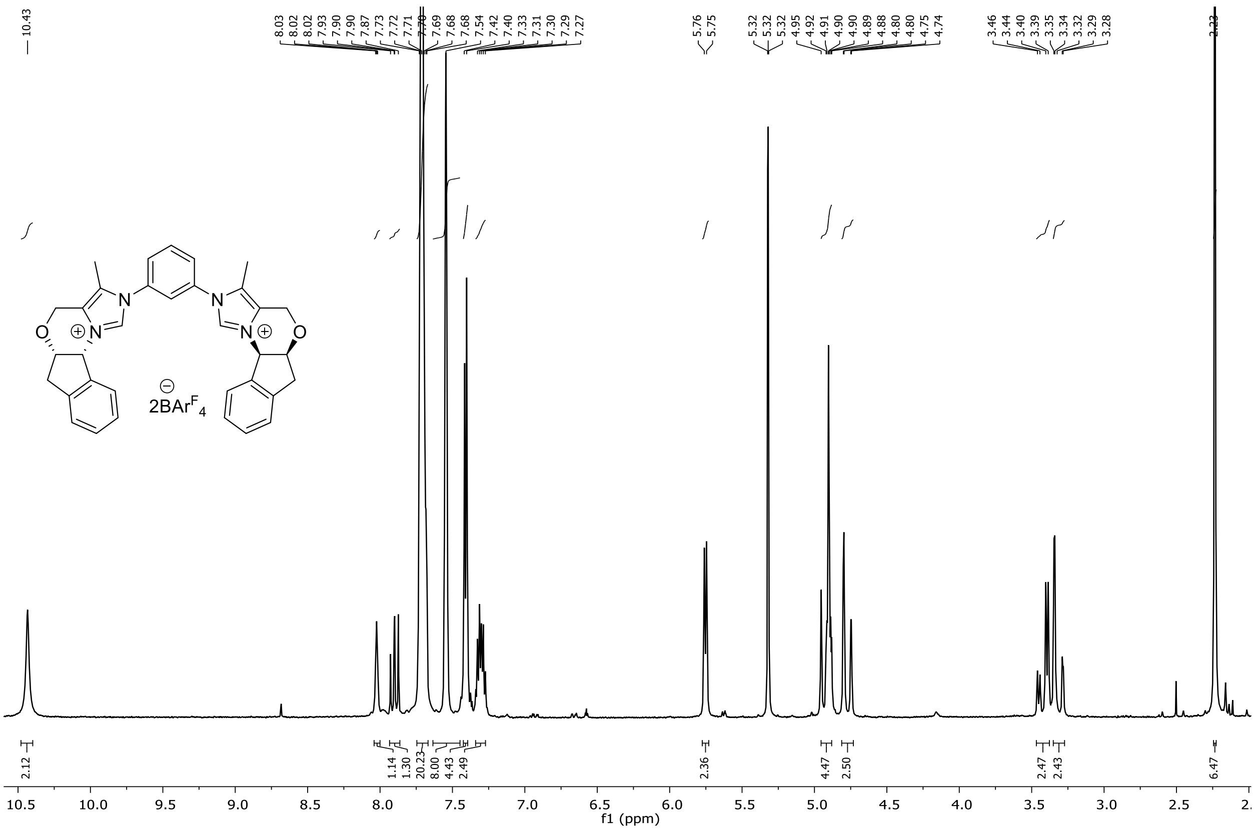

^{13}C NMR (75 MHz, CD_2Cl_2) of **4b.BAr$^F_4$**

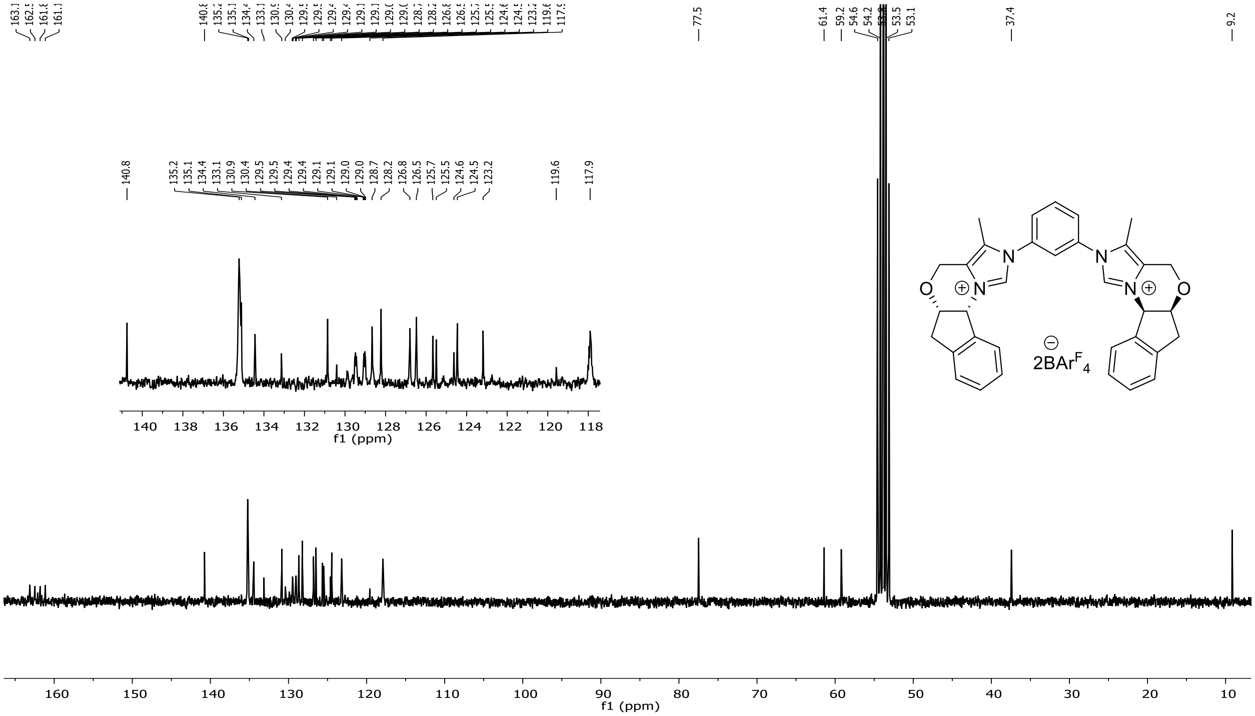

^{19}F NMR (235 MHz, $CDCl_3$) of **4b.BAr$^F_4$**

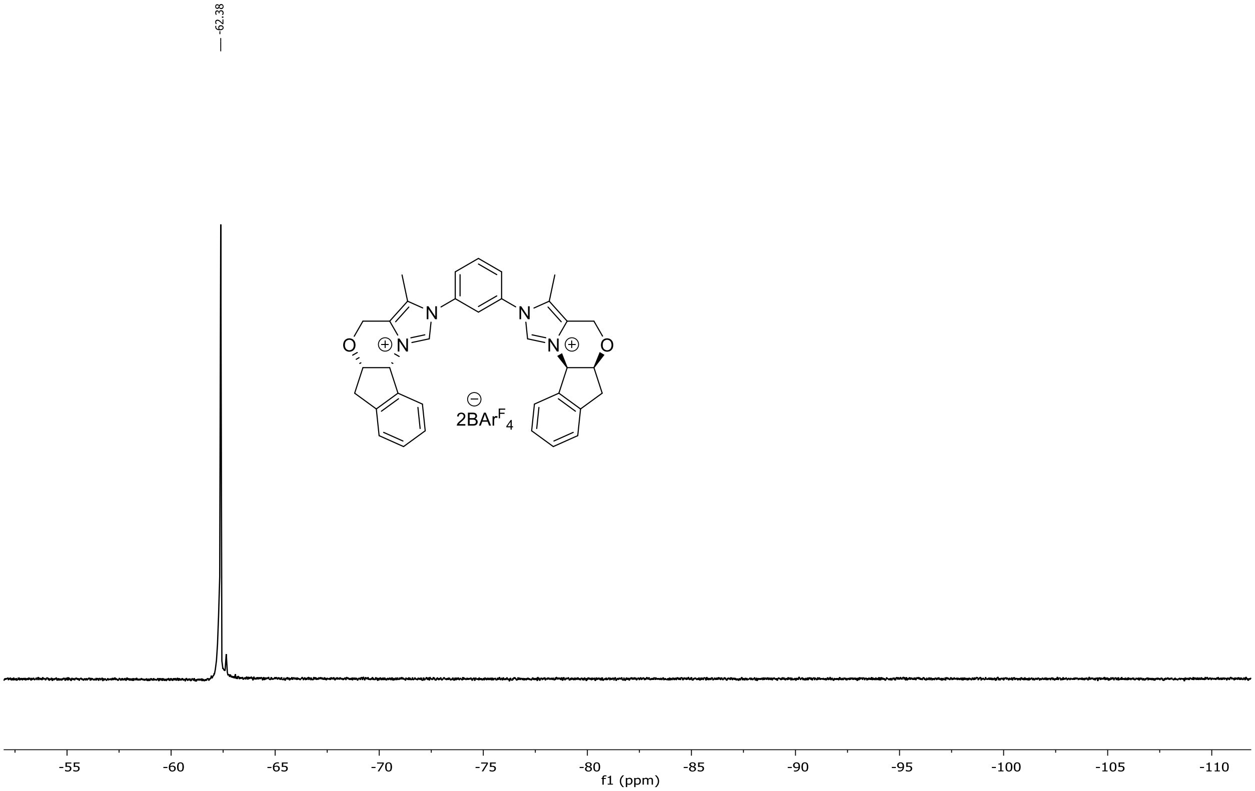

^{1}H NMR (300 MHz, CD_2Cl_2) of **1b.Cl**

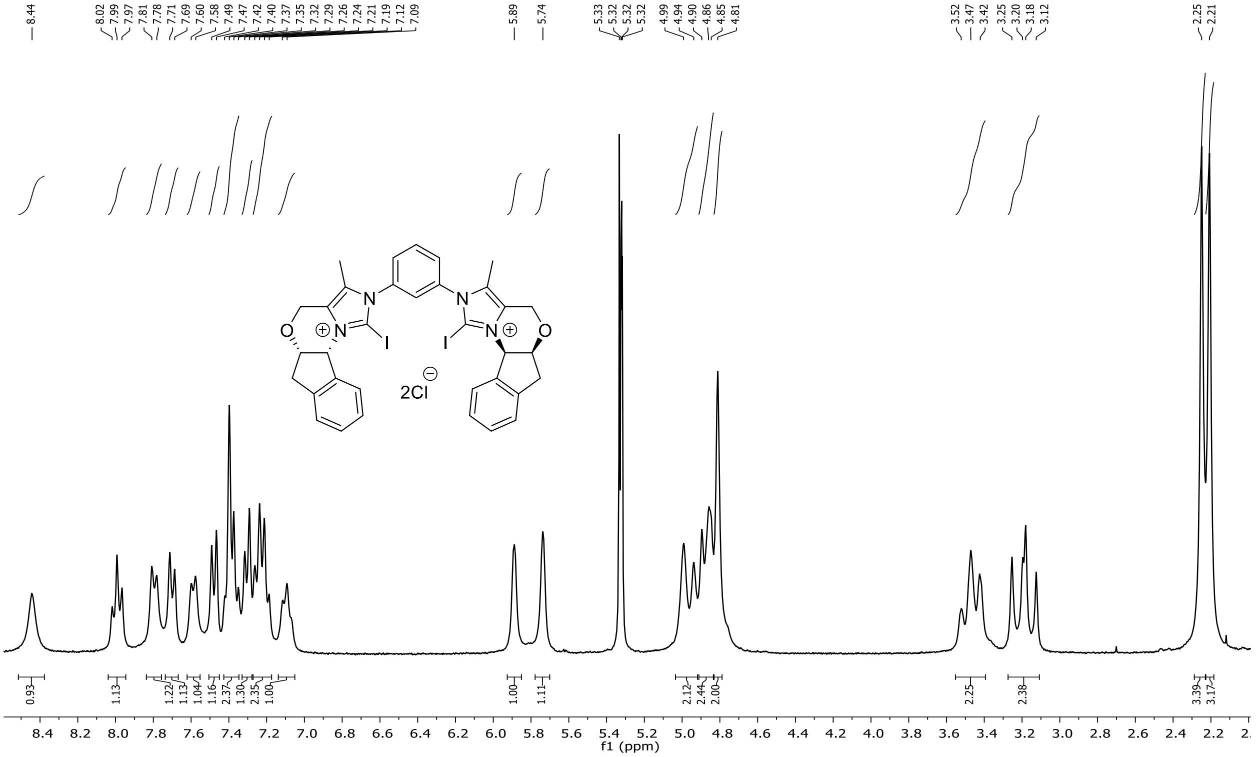

^{13}C NMR (75 MHz, DMSO-*d6*) of **1b.Cl**

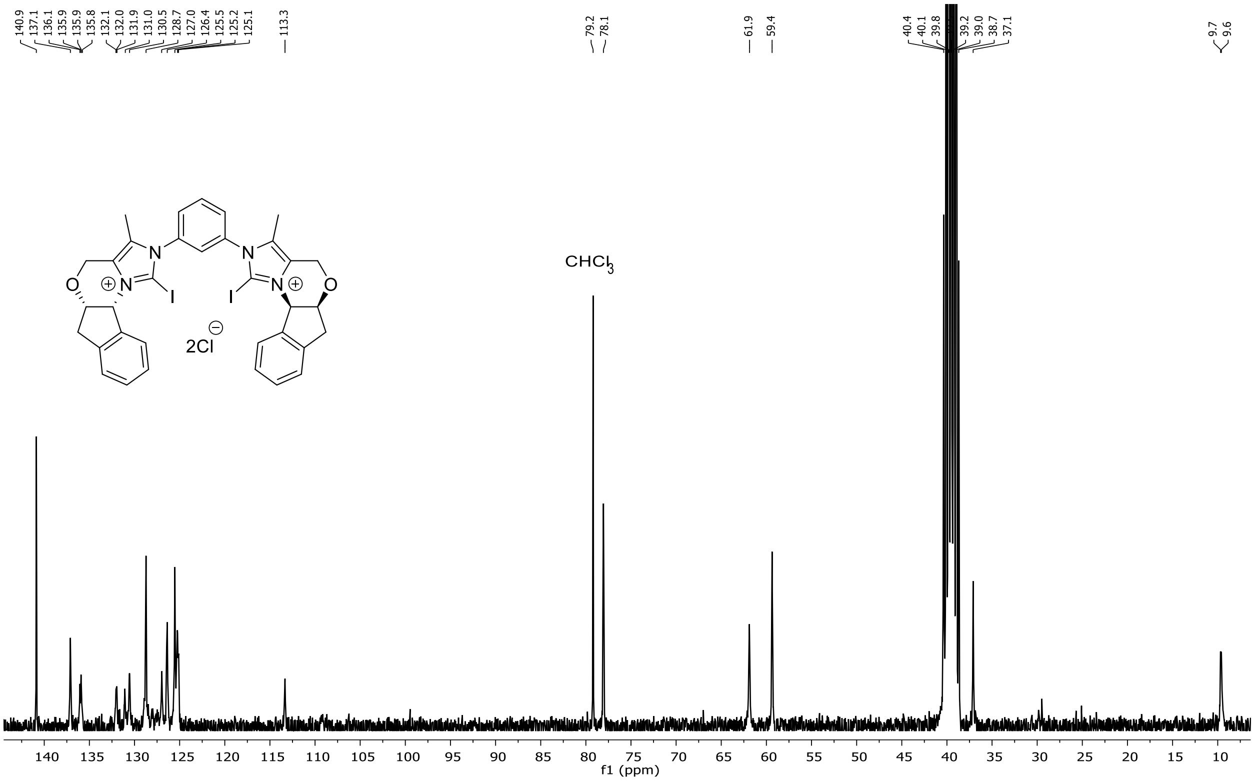

^{1}H NMR (250 MHz, DMSO-*d6*) of **1c.Cl**

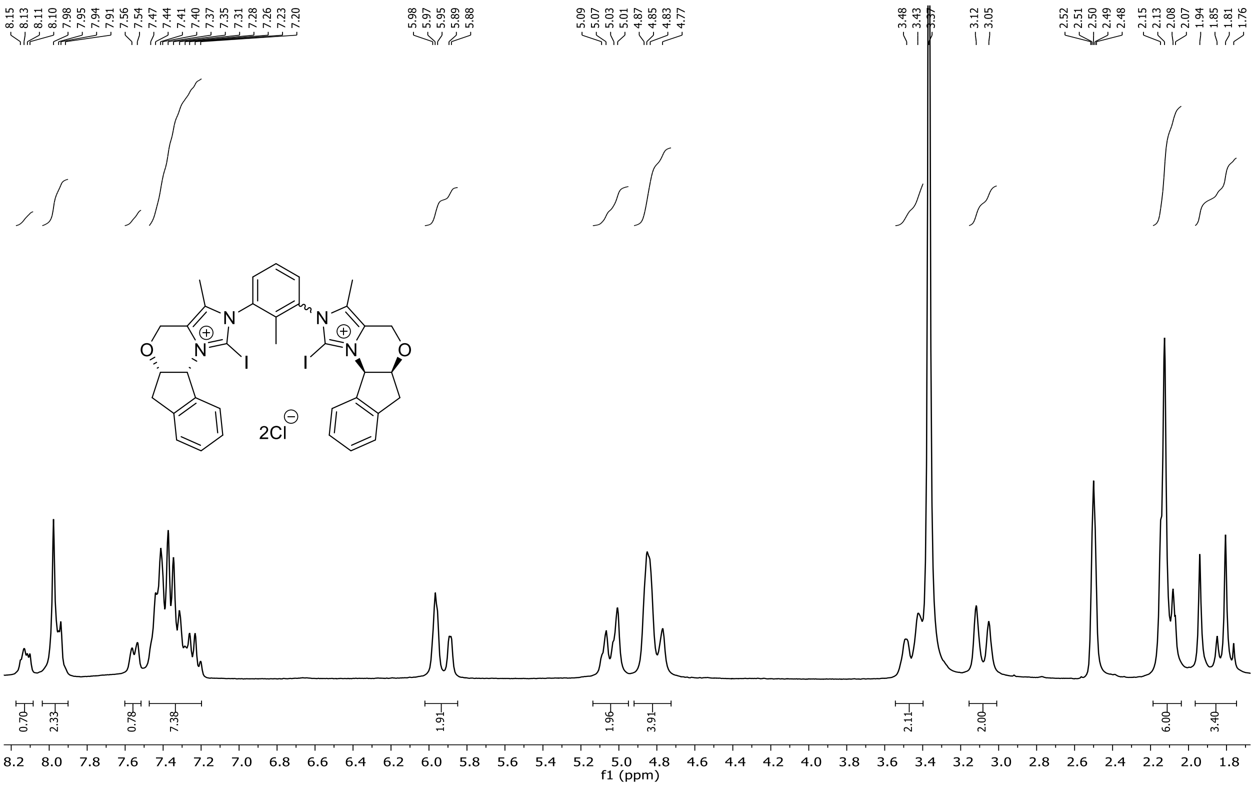

^{13}C NMR (63 MHz, DMSO-*d6*) of **1c.Cl**

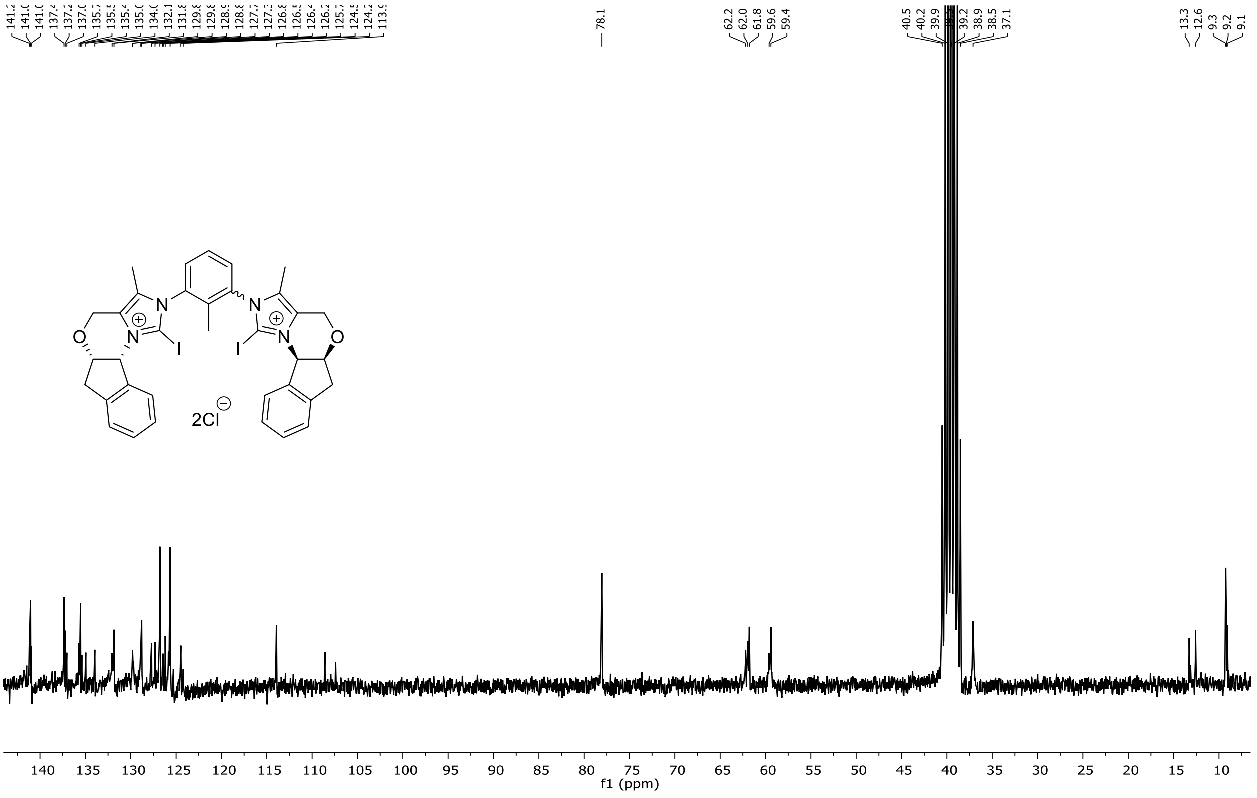

^{1}H NMR (300 MHz, CD_2Cl_2) of **1b.OTf**

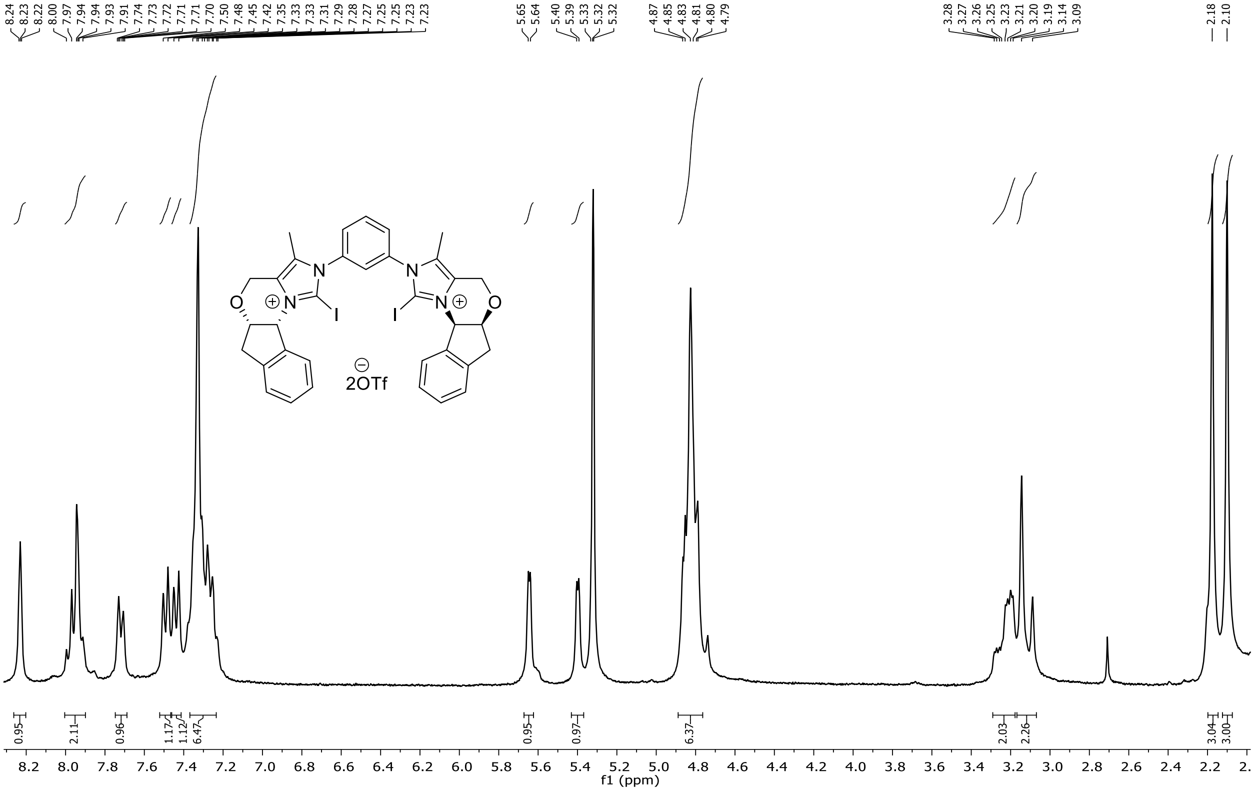

^{13}C NMR (75 MHz, CD_3CN) of **1b.OTf**

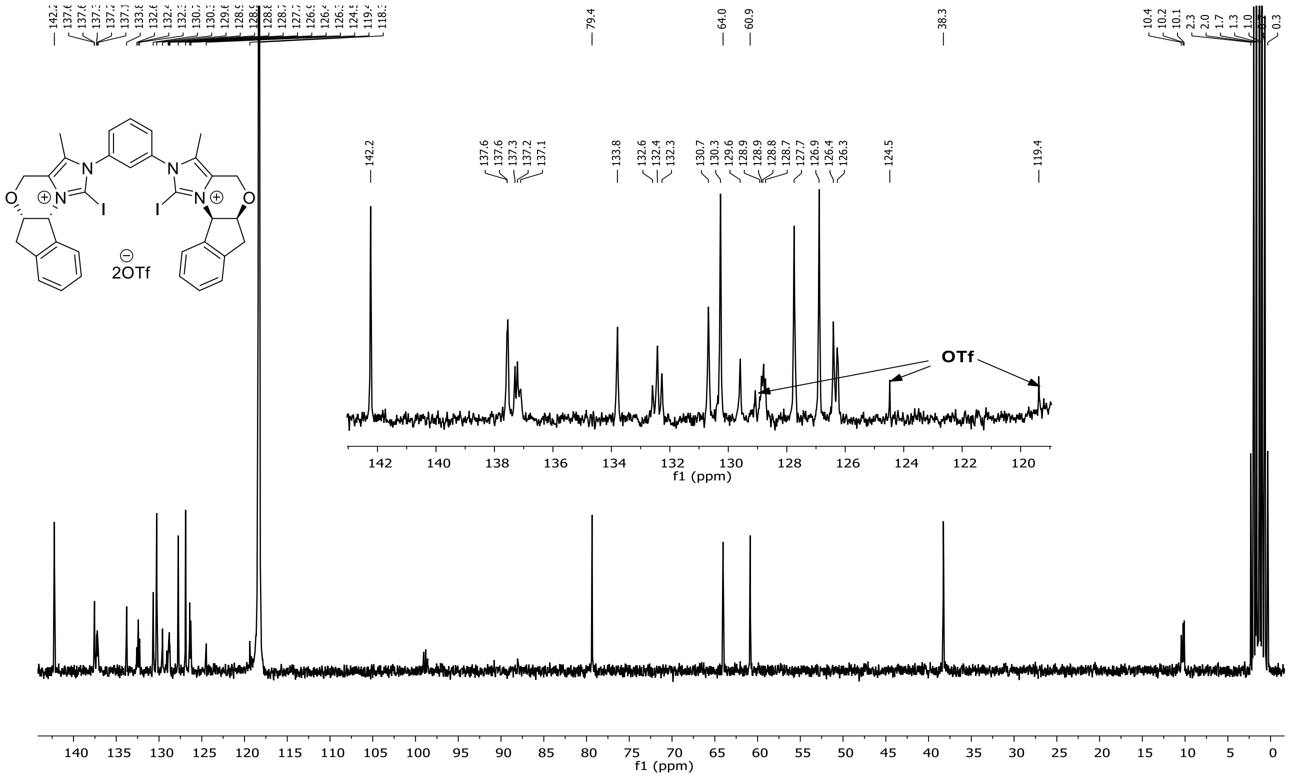

^{19}F NMR (235 MHz, CD_2Cl_2) of **1b.OTf**

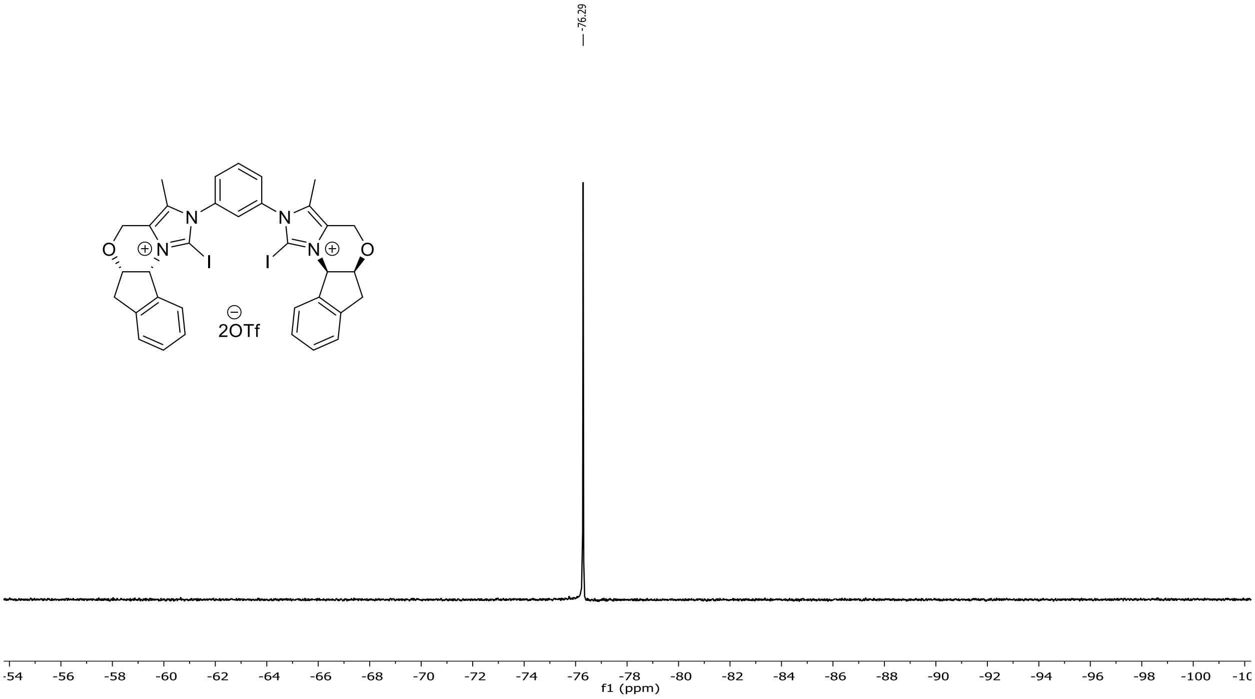

^{1}H NMR (250 MHz, CD_2Cl_2) of *syn*-**1c.OTf**

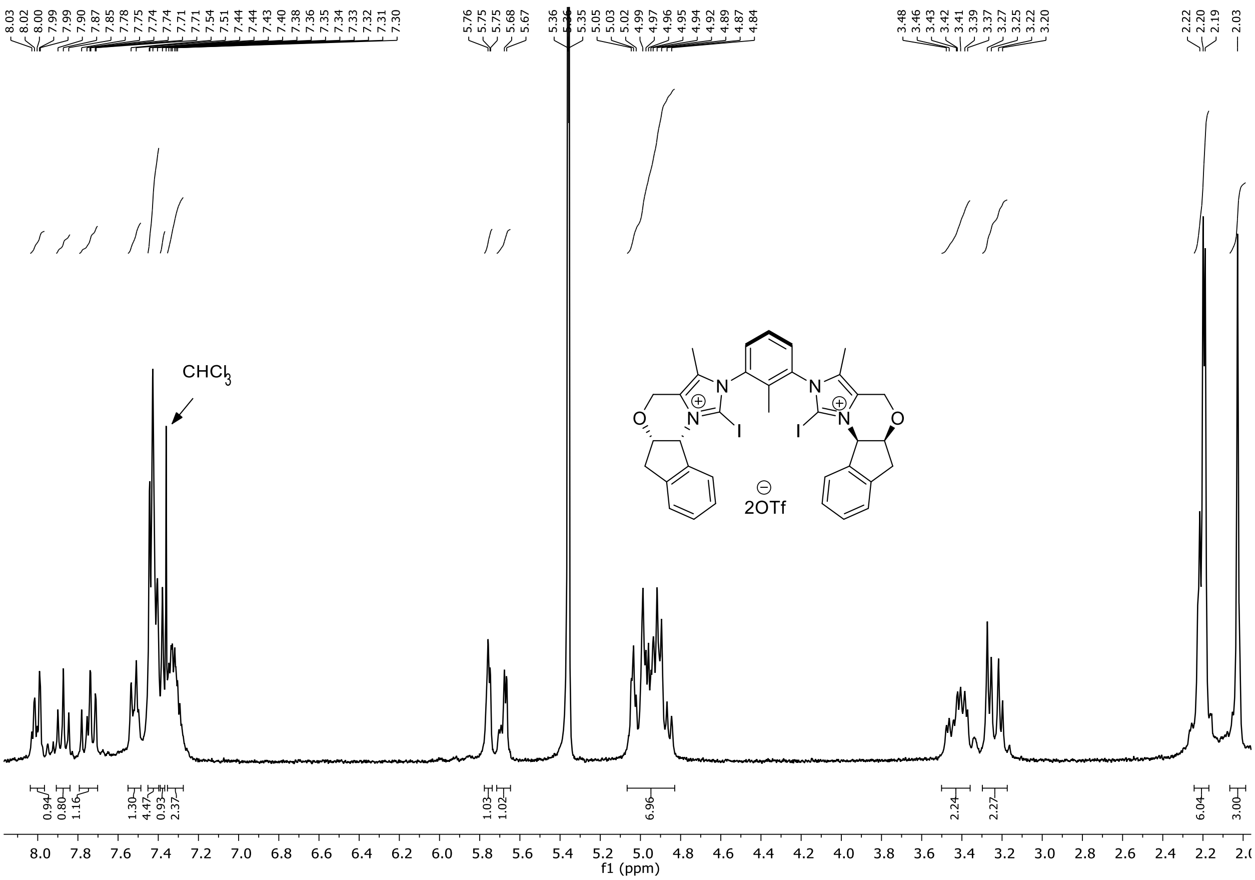

^{13}C NMR (63 MHz, CD_3CN) *syn*-**1c.OTf**

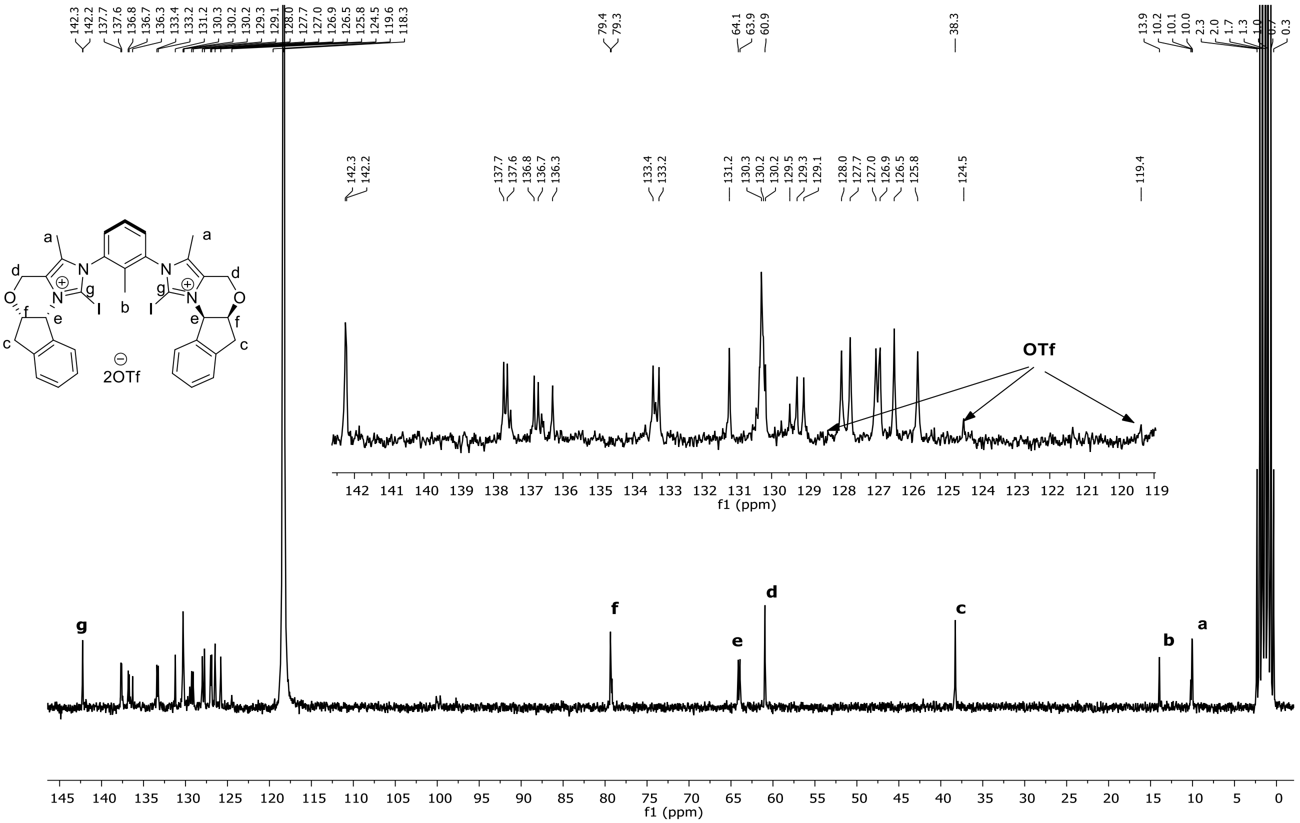

^{19}F NMR (235 MHz, CD_2Cl_2) of *syn*-**1c.OTf**

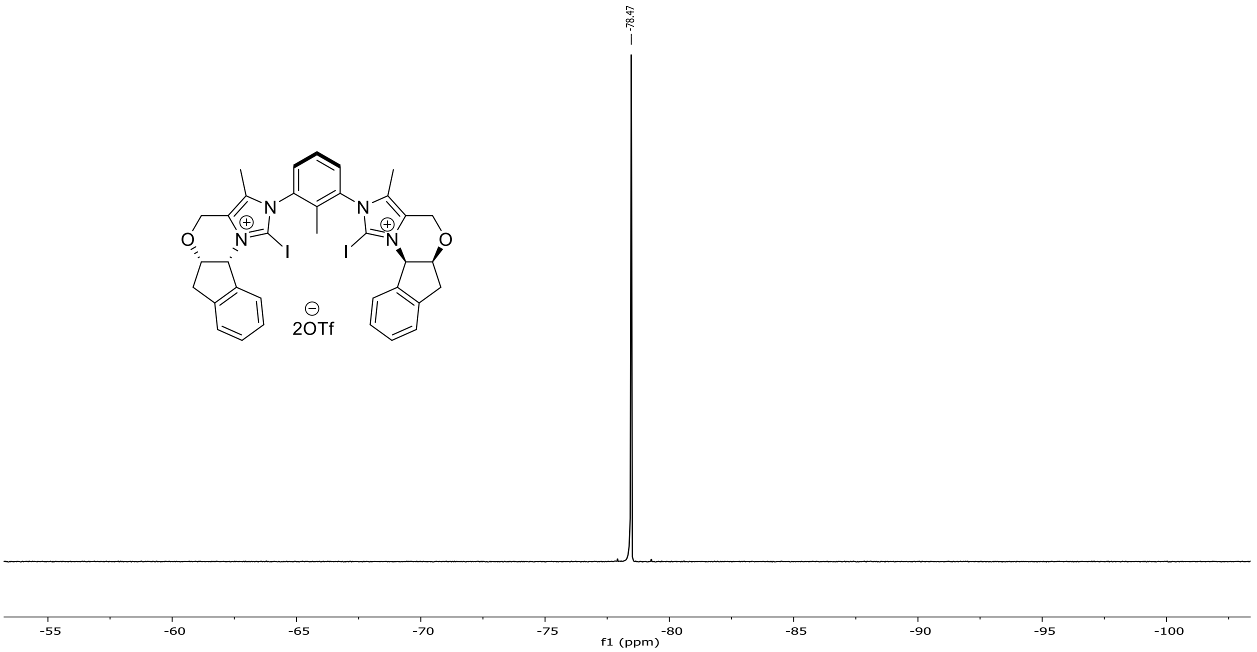

Variable temperature ^{1}H NMR (400 MHz, CD_3CN) of *syn*-**1c.OTf** (Heating experiment)

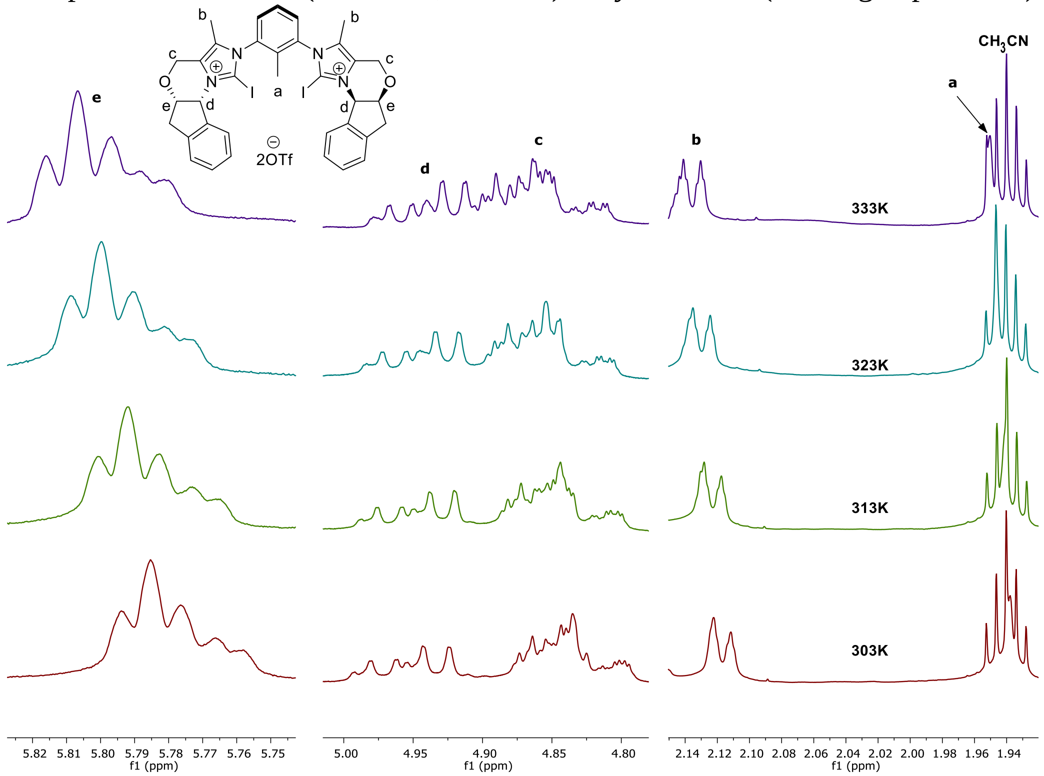

Variable temperature ^{1}H NMR (400 MHz, CD_3CN) of *syn*-**1c.OTf** (Cooling experiment)

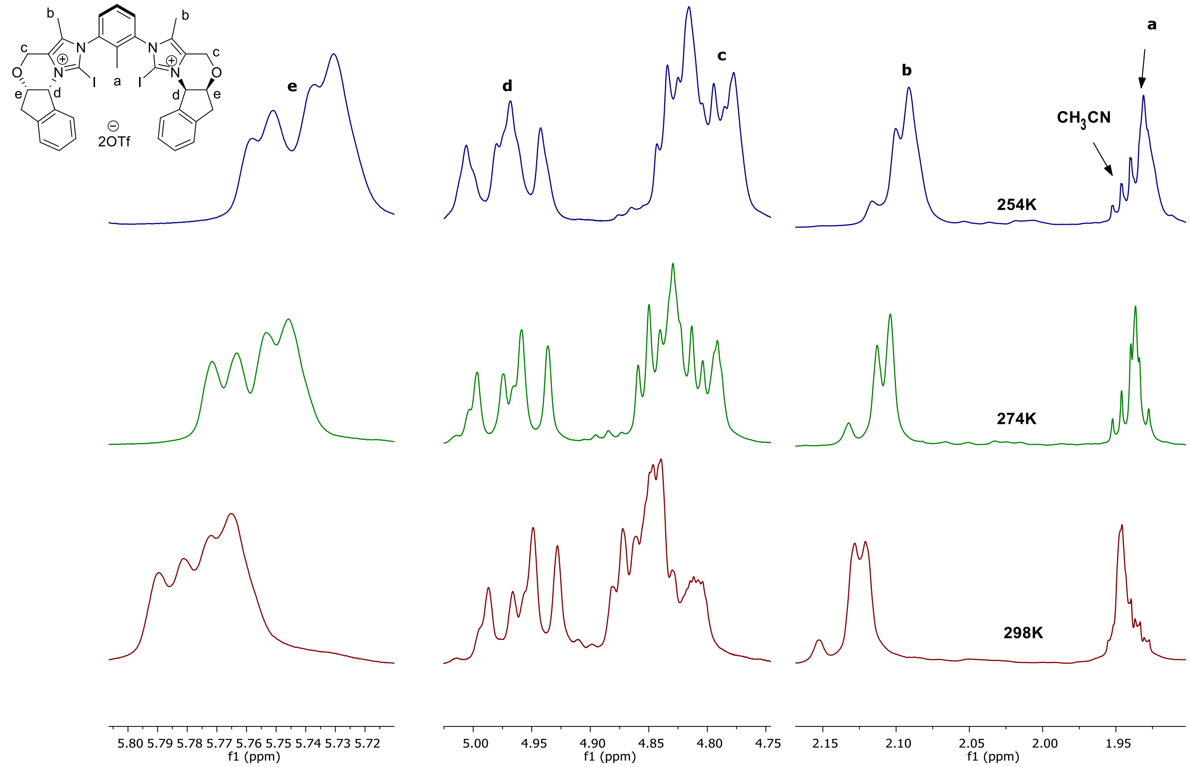

DOSY (300 MHz, CD_3CN) of *syn*-**1c.OTf**

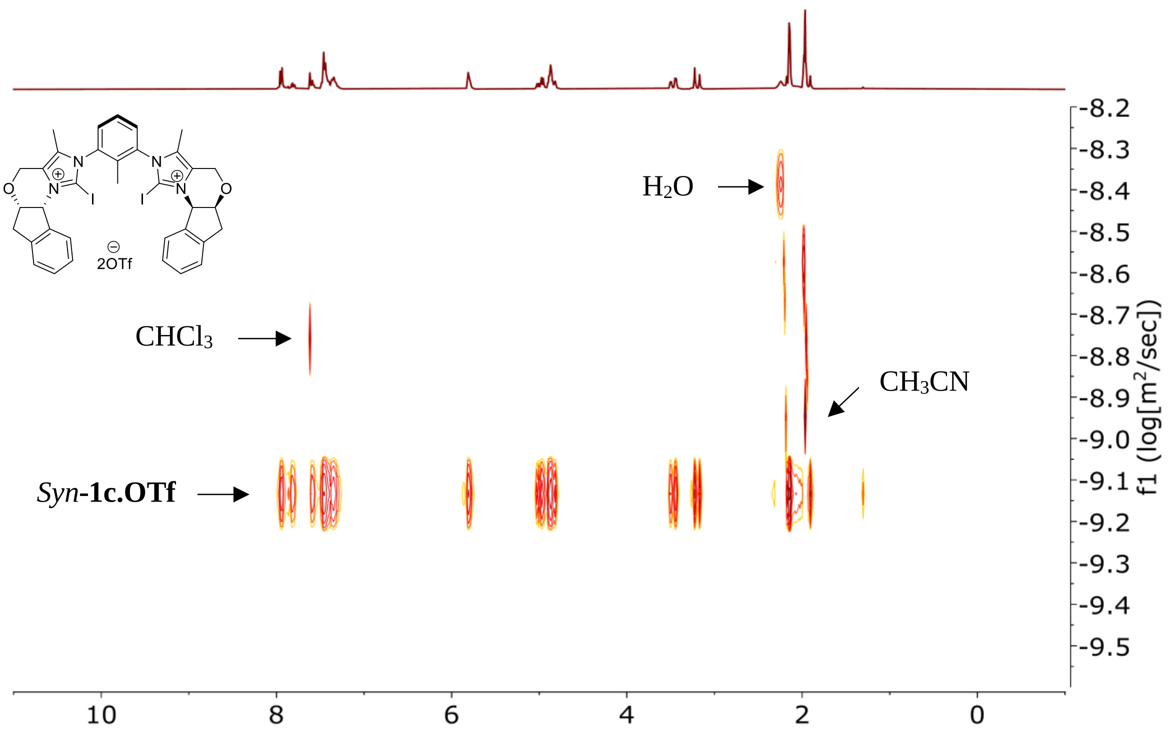

^{1}H NMR (250 MHz, CD_3CN) of *anti*-**1c.OTf**

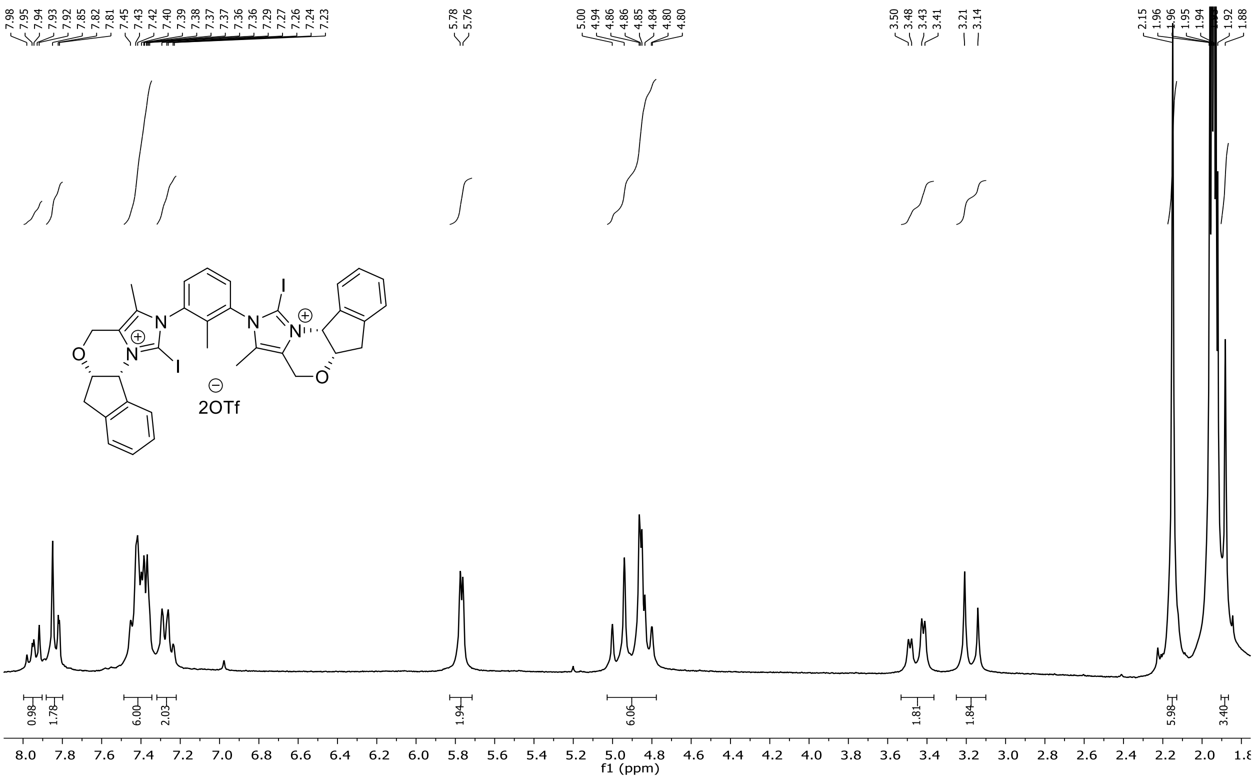

^{13}C NMR (63 MHz, CD_3CN) of *anti*-**1c.OTf**

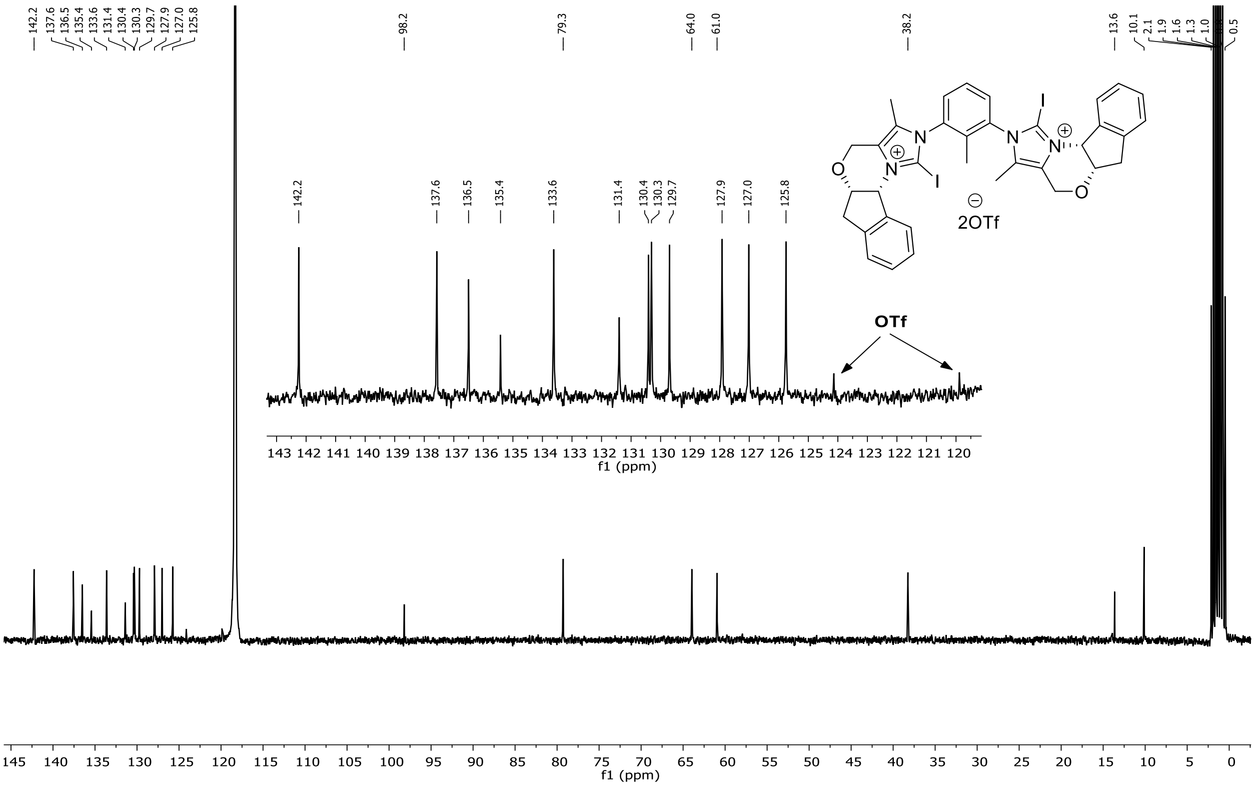

^{19}F NMR (235 MHz, CD_3CN) of *anti*-**1c.OTf**

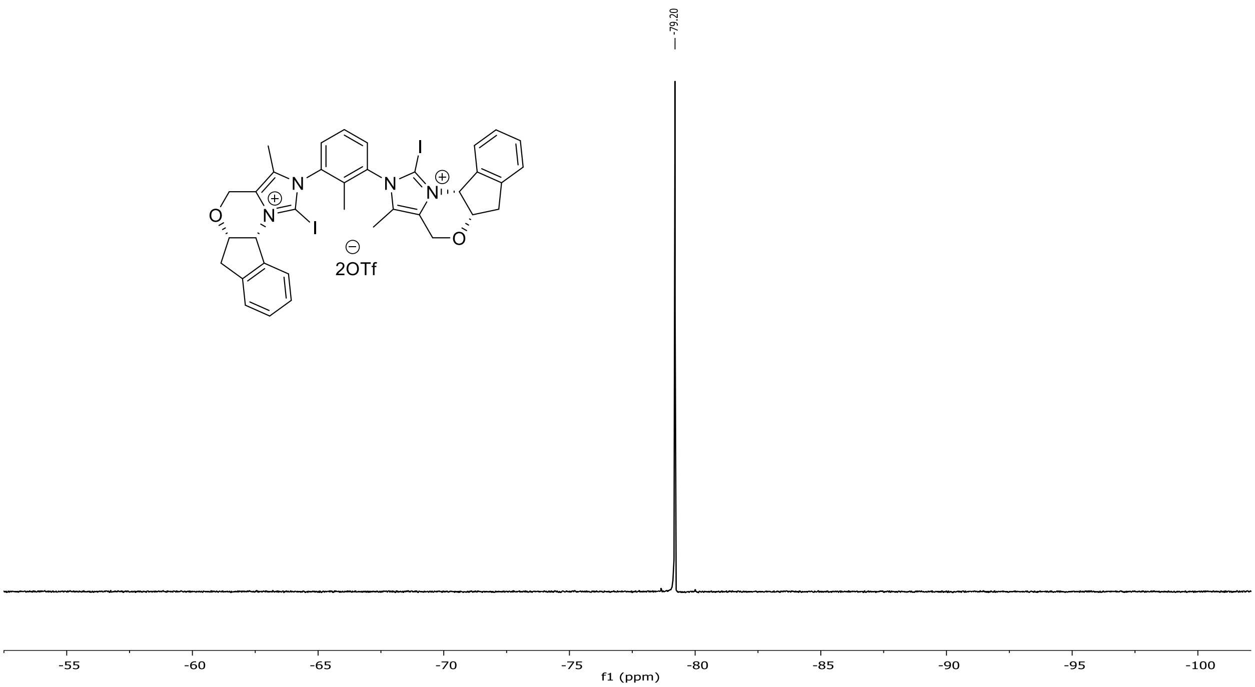

Stacked ^{1}H NMR (250 MHz, CD_3CN) of *syn*- and *anti*-**1c.OTf**

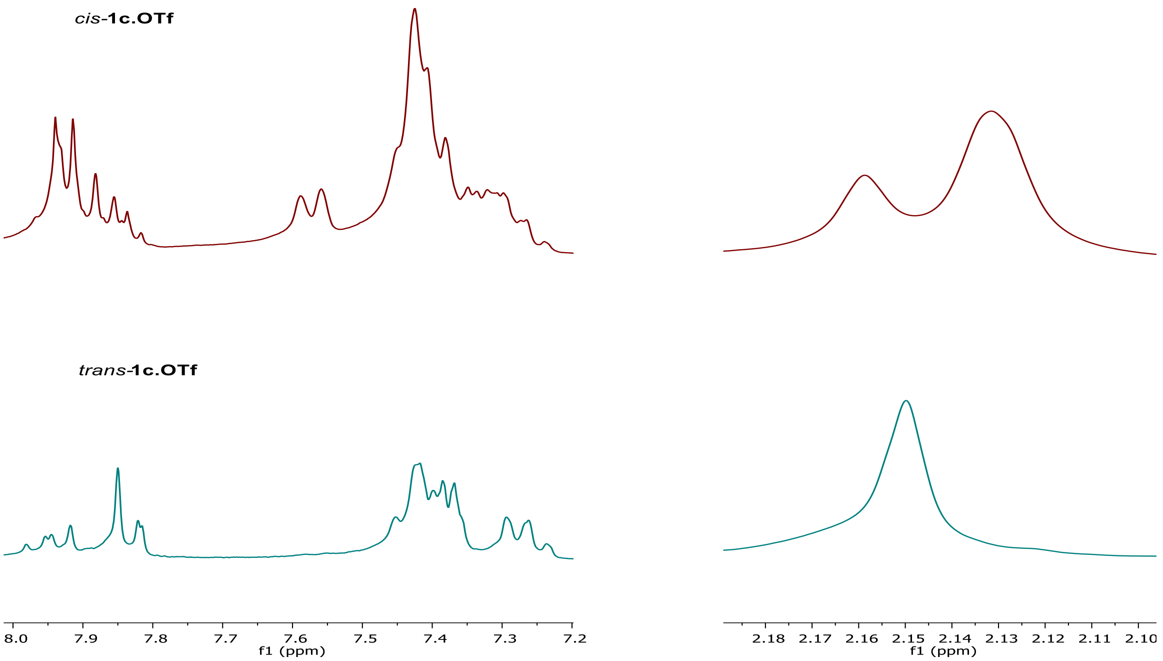

^{1}H NMR (300 MHz, CD_3CN) of **1b.BAr$^F_4$**

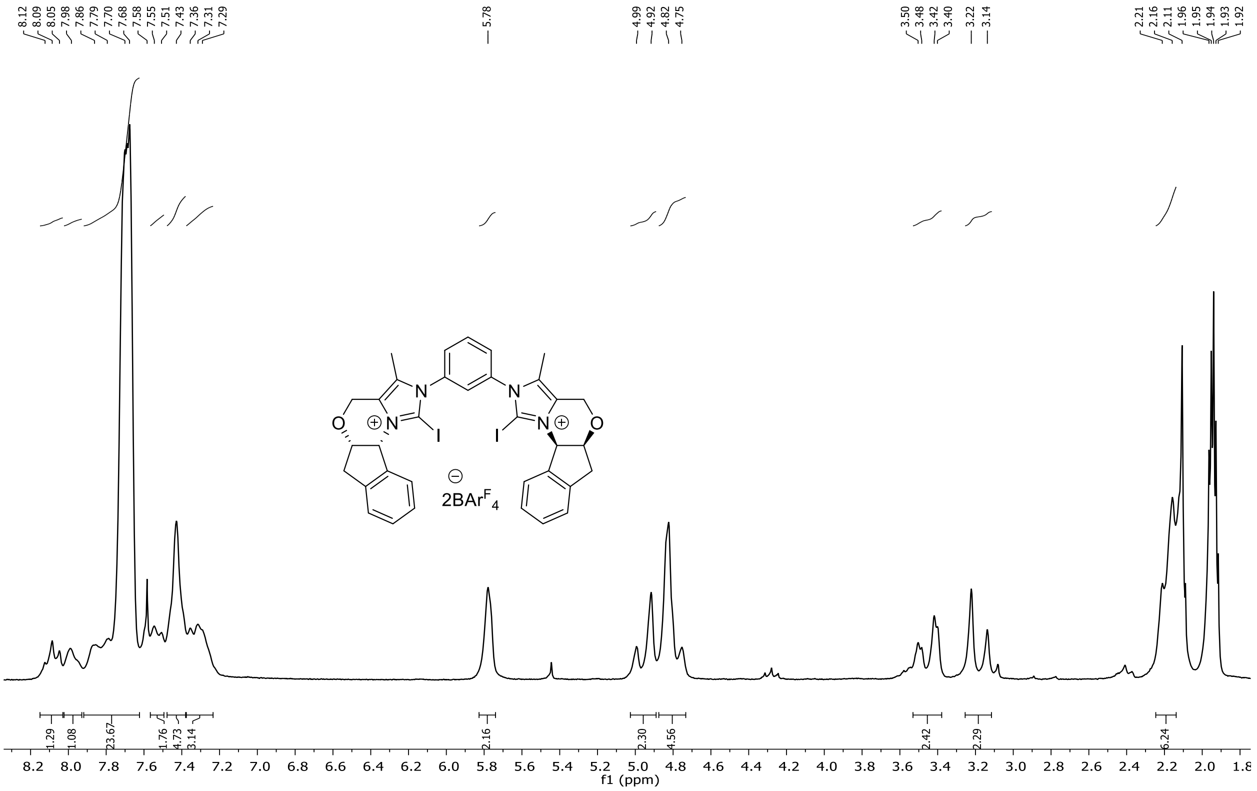

^{13}C NMR (75 MHz, CD_2Cl_2) of **1b.BArF4**

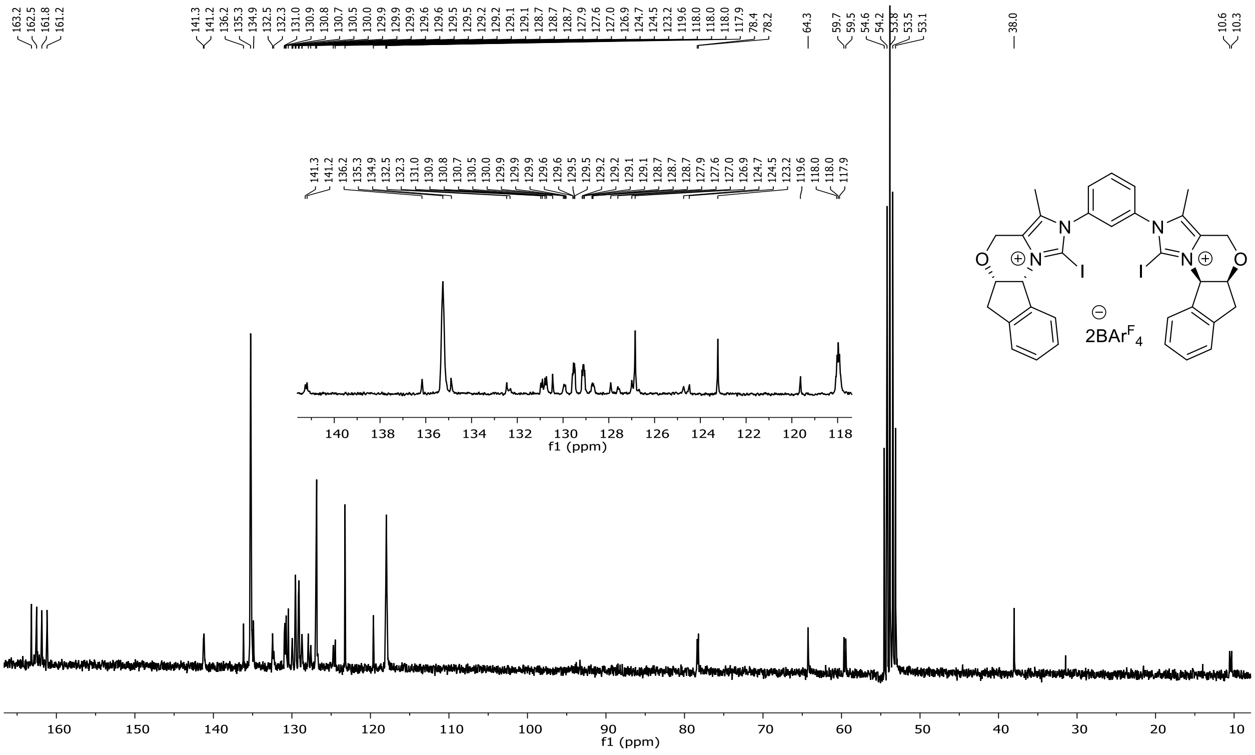

^{19}F NMR (235 MHz, $CDCl_3$) of **1b.BArF4**

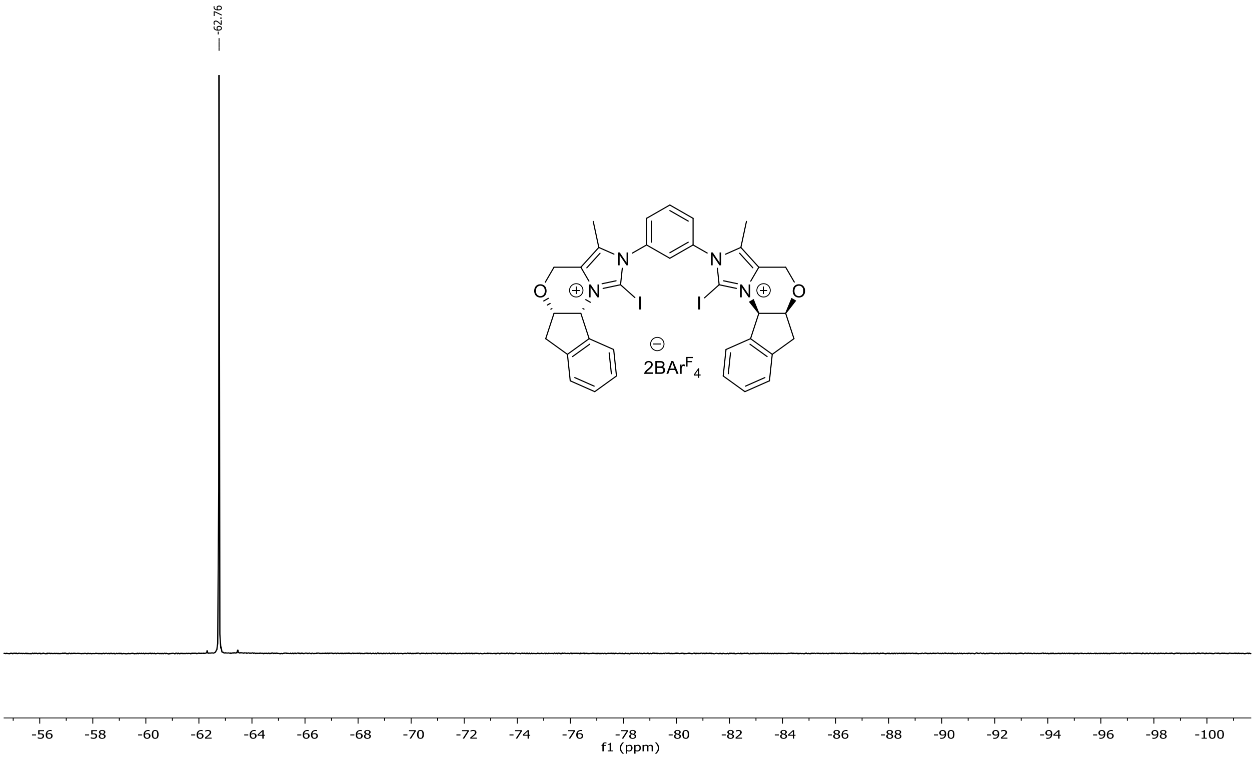

^{1}H NMR (300 MHz, CD_2Cl_2) of *syn*-**1c.BAr$^{F}_{4}$**

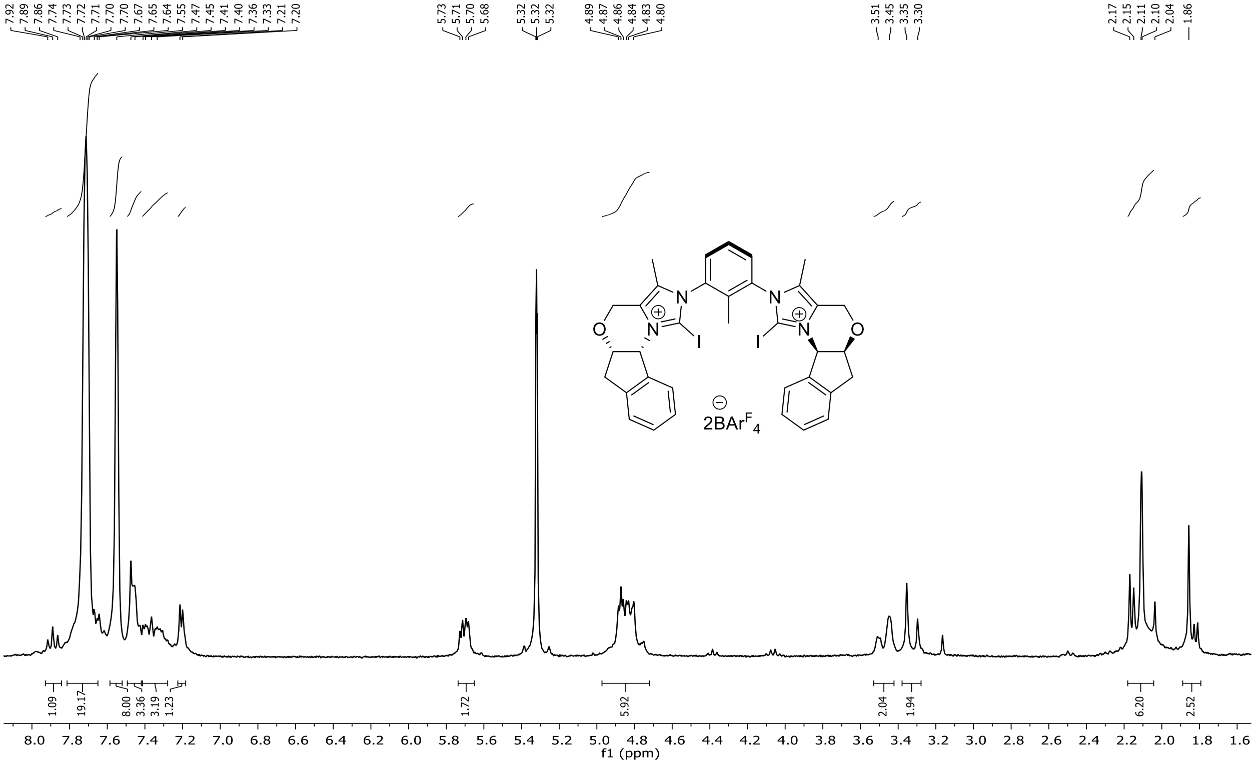

^{13}C NMR (125 MHz, CD_2Cl_2) of *syn*-**1c.BAr$^{F}_{4}$**

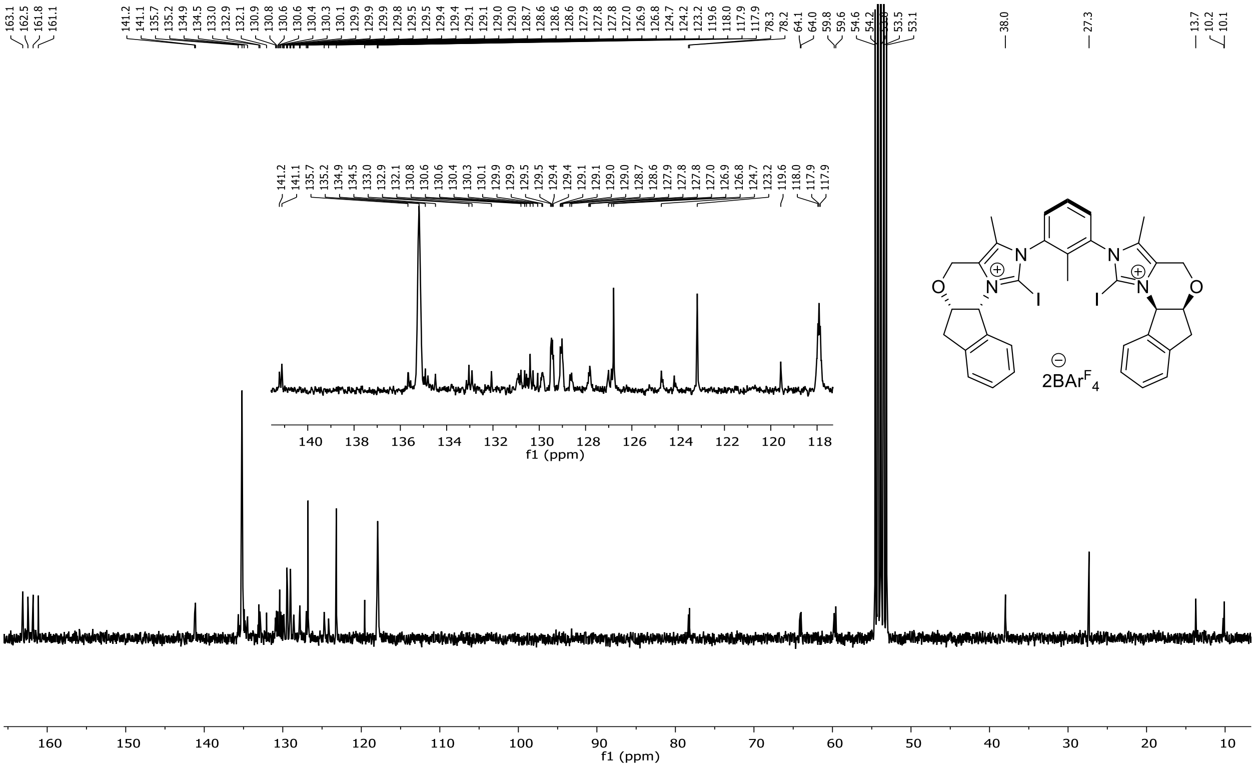

^{19}F NMR (235 MHz, CD_2Cl_2) of *syn*-**1c.BArF4**

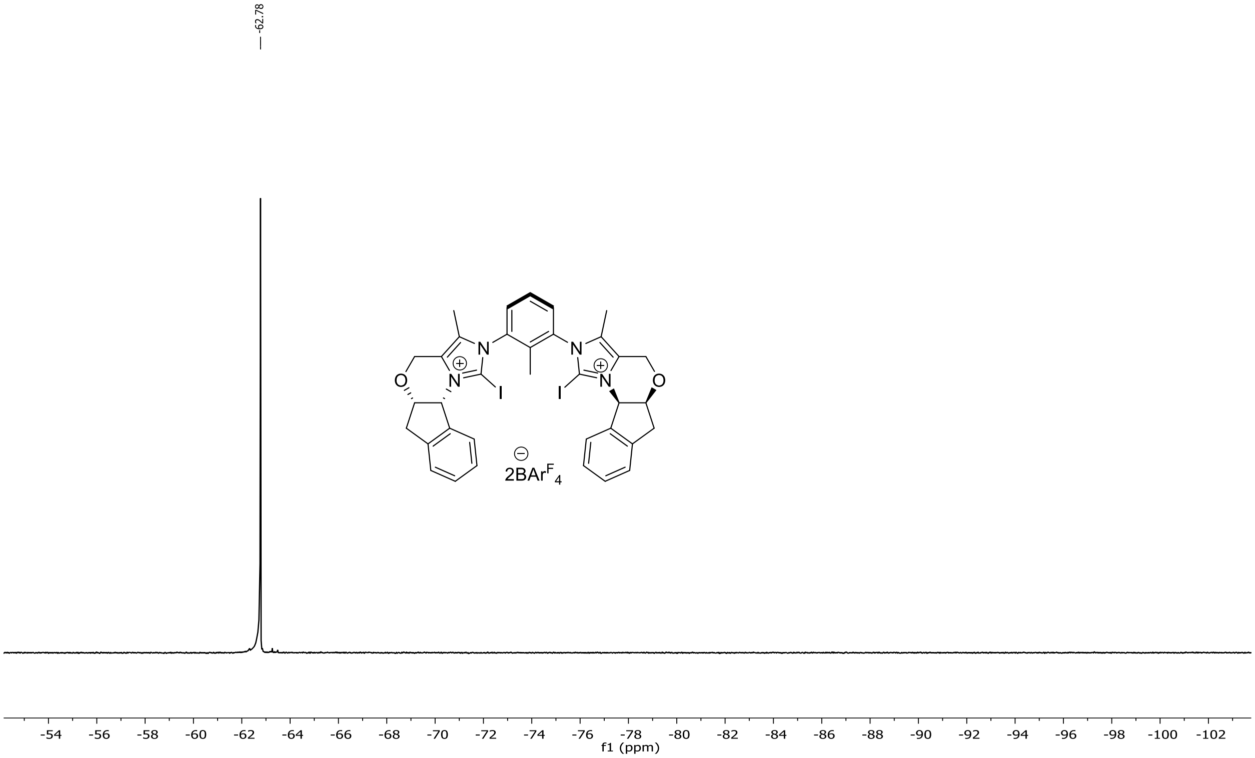

6. DFT Calculations

DFT calculations were performed on the adducts between halogen bond donor **1b.Bar$^{F}_{4}$** and both enantiomers of ***trans*-11**. For details on the methodology please see the corresponding paragraph in the paper. Both complexes very fully optimized and the identity of the minima was confirmed by frequency calculations, which yielded no imaginary frequencies. The coordinates of the structures are given below.

<u>**6.1.** Adduct of **1b.BAr$^{F}_{4}$** with **(1*R*,2*R*)-11**</u>

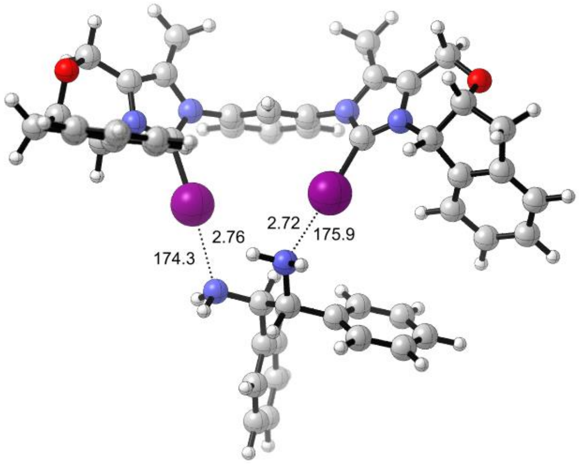

(picture above: I···N distances in Å, C-I···N angles in °)

C	0.94634400	-2.09303700	-1.48704700
C	0.59247300	-1.47442100	-4.18204500
C	1.99638900	-1.60872900	-2.24948800
C	-0.29033000	-2.24890400	-2.09388200
C	-0.47779000	-1.94351400	-3.43314200
C	1.83605300	-1.30167000	-3.59186500
H	-1.45621100	-2.06344400	-3.88023600
H	2.67420200	-0.92235100	-4.16224100
C	-1.98364100	-2.11646300	-0.30052900
N	-2.92541600	-2.93492800	0.16621400
H	0.45348600	-1.23235200	-5.22655200
C	-2.92113900	-4.11682200	-0.55092000

N	-1.38505300	-2.75519400	-1.32363200
C	-3.85602500	-2.67771100	1.27343500
C	-4.47473900	-4.00583900	1.76448500
H	-3.27399600	-2.21228500	2.07336600
C	-3.81155800	-5.24704100	-0.12666000
O	-4.87665600	-4.77408200	0.64756500
H	-3.78892100	-4.58251000	2.39395300
H	-3.22023900	-5.98184700	0.43599800
H	-4.23891600	-5.74446200	-0.99602900
C	-5.75085500	-3.55590700	2.46990900
H	-6.48884700	-4.35637000	2.47076200
H	-5.53708000	-3.28504000	3.50689400
C	-6.16104900	-2.35286500	1.65815800
C	-5.08090000	-1.85521500	0.93315500
C	-7.40672400	-1.76400500	1.54710100
C	-7.56161600	-0.68190300	0.68750300
C	-5.22531000	-0.77665100	0.07984600
C	-6.48467000	-0.19775800	-0.04279700
H	-8.25264700	-2.15114100	2.10061900
H	-8.53457100	-0.22226700	0.57507300
H	-6.62897800	0.63324300	-0.71942800
H	-4.39397600	-0.38454700	-0.49421400
N	3.27778300	-1.44239000	-1.63025400
C	3.61659200	-0.43820900	-0.80286100
N	4.87155300	-0.66171900	-0.41662500
C	5.35075000	-1.81139800	-1.01462100
C	5.65898500	0.13358100	0.53263600
C	6.77977300	-2.21219000	-0.79820100
C	7.15283100	-0.25178500	0.45018700
H	5.52370700	1.18365700	0.26016100
C	5.34003300	-0.10245800	1.99328700
O	7.26861400	-1.65982400	0.39223800
H	7.38406000	-1.88373700	-1.65391800

H	6.86493600	-3.29456800	-0.71426500
H	7.65715300	0.20428800	-0.40780900
C	7.70160700	0.18729300	1.80612300
C	6.52267000	-0.02555700	2.72268600
C	4.12937100	-0.35400200	2.61333200
H	8.57547800	-0.40490300	2.07291100
H	7.99790300	1.23864300	1.77347700
C	6.50230800	-0.17058700	4.09831400
C	4.11260600	-0.51028700	3.99541900
H	3.20989300	-0.44719600	2.04828000
C	5.28711900	-0.40924500	4.73056800
H	7.41821300	-0.11903600	4.67304300
H	3.17980900	-0.71840000	4.50226100
H	5.25842800	-0.53495300	5.80469200
C	-1.95918800	-4.01302300	-1.50004500
C	4.35151700	-2.31626400	-1.78101000
I	2.47220100	1.26668000	-0.39919100
I	-1.43880700	-0.24042800	0.46798000
C	4.29124700	-3.52742000	-2.63879200
H	3.42777100	-4.14364600	-2.38523200
H	4.22425400	-3.26776100	-3.69607500
H	5.19019700	-4.12273000	-2.49332600
C	-1.49664000	-4.98405800	-2.52538400
H	-1.72194700	-4.64295900	-3.53624200
H	-0.42074300	-5.14926500	-2.45454200
H	-1.99716400	-5.93801300	-2.37305400
H	1.08914900	-2.33964900	-0.44247300
C	-1.10034900	3.36600600	1.00721000
C	-0.39039500	3.61033200	-0.32575400
H	-0.58481400	2.75600800	-0.98212400
H	-0.83842000	4.19706500	1.67468000
N	1.07539500	3.62473200	-0.09425200
H	1.29304100	4.23635500	0.69105300

H	1.51285300	4.07167900	-0.89690400
N	-0.63053500	2.09235400	1.59902600
H	-1.05121600	1.98590800	2.51853000
H	0.37681900	2.10962100	1.73495400
C	-2.60949000	3.31908800	0.91476000
C	-3.35175900	3.65868800	2.04156100
C	-3.28071100	2.90102000	-0.23104100
C	-4.73705600	3.57263800	2.03419600
H	-2.84249600	4.01108900	2.93272700
C	-4.66519300	2.82799400	-0.24544900
H	-2.73313700	2.65041800	-1.13039400
C	-5.39670400	3.15578500	0.88780900
H	-5.29930300	3.84658100	2.91679200
H	-5.17367100	2.52987600	-1.15337300
H	-6.47716100	3.09993400	0.87348600
C	-0.86282800	4.87060400	-1.01802900
C	-1.24771400	4.82422400	-2.35314300
C	-0.87417000	6.09607600	-0.35502200
C	-1.64677800	5.97655900	-3.01669200
H	-1.24110600	3.87582000	-2.88001300
C	-1.27311700	7.24829800	-1.01464700
H	-0.57330500	6.16381700	0.68444300
C	-1.66186500	7.19047600	-2.34675600
H	-1.94685100	5.92551500	-4.05489700
H	-1.28290400	8.19330200	-0.48813200
H	-1.97455100	8.09015400	-2.85943400

6.2. Adduct of **1b.BAr$^F_4$** with **(1S,2S)-11**

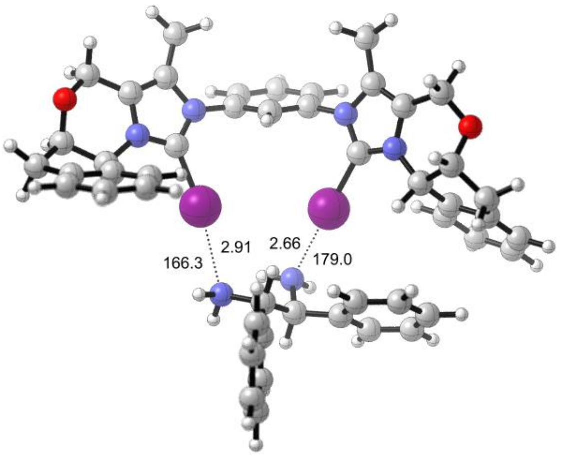

(picture above: I···N distances in Å, C-I···N angles in °)

C	0.91602000	-2.80520800	-0.20831800
C	0.70545800	-4.46463300	-2.43990500
C	2.00094500	-3.00958100	-1.04506900
C	-0.28168200	-3.43326200	-0.51941400
C	-0.39629400	-4.26789900	-1.61885700
C	1.90497200	-3.82762600	-2.16208200
H	-1.34576600	-4.73797300	-1.84081200
H	2.76216100	-3.95798400	-2.80971100
C	-2.11635400	-2.01351000	0.25606400
N	-3.09717100	-2.11528500	1.14890400
H	0.62342600	-5.10281900	-3.30871700
C	-3.03891200	-3.34774300	1.77409400
N	-1.43107600	-3.16912700	0.29299200
C	-4.08505100	-1.08297300	1.48548700
C	-4.75216900	-1.41838400	2.83582900
H	-3.54151600	-0.13457800	1.54974900
C	-3.98589500	-3.64647700	2.89994800
O	-5.09404300	-2.79052500	2.85389400

H	-4.11267200	-1.17857900	3.69203100
H	-3.45795900	-3.54267800	3.85710500
H	-4.36161900	-4.66584200	2.82469000
C	-6.05640000	-0.62607000	2.78944900
H	-6.81522100	-1.10204300	3.40871000
H	-5.89573400	0.38756600	3.16621400
C	-6.39006800	-0.62190100	1.31828900
C	-5.27050900	-0.94384200	0.55590000
C	-7.60397400	-0.37198900	0.70346200
C	-7.68510900	-0.46033700	-0.68248100
C	-5.34786500	-1.05017100	-0.82063700
C	-6.57083600	-0.80618900	-1.43678700
H	-8.48181100	-0.13156900	1.28999600
H	-8.63084200	-0.27984300	-1.17620100
H	-6.65656200	-0.89357500	-2.51166900
H	-4.48968600	-1.32575000	-1.41930500
N	3.26470000	-2.41283000	-0.72649400
C	3.55493300	-1.09854400	-0.70074300
N	4.83485600	-0.98796800	-0.34320900
C	5.37930600	-2.24156300	-0.15389300
C	5.60007700	0.24899200	-0.13857600
C	6.84021400	-2.37765000	0.15817500
C	7.11400100	-0.05524300	-0.10373500
H	5.36952300	0.90957600	-0.97831800
C	5.37736700	0.94528600	1.18747200
O	7.34117500	-1.19126500	0.70650200
H	7.38330800	-2.64048700	-0.75909700
H	7.00187000	-3.16859000	0.88934600
H	7.53409900	-0.21203300	-1.10251100
C	7.69186600	1.14748500	0.63886500
C	6.58487000	1.50095700	1.60056200
C	4.22982900	1.07845300	1.94861200
H	8.62673500	0.87971900	1.12859700

H	7.89279300	1.96413500	-0.05895100
C	6.64991800	2.23228900	2.77286300
C	4.29993400	1.80502600	3.13277100
H	3.29491500	0.61890600	1.65353400
C	5.49647200	2.38521500	3.53421300
H	7.58652500	2.66179000	3.10471100
H	3.41820400	1.91327000	3.75015200
H	5.53594900	2.94565100	4.45868700
C	-1.99420400	-4.02697900	1.23569000
C	4.39897500	-3.14949300	-0.38574400
I	2.34521700	0.51049600	-1.22833300
I	-1.66253100	-0.36294000	-0.95323900
C	4.42000100	-4.63302700	-0.29781800
H	3.55057000	-5.00542400	0.24442700
H	4.43278300	-5.10138700	-1.28238100
H	5.31525100	-4.94827300	0.23473500
C	-1.44913100	-5.37847500	1.52371200
H	-1.51277400	-6.02875300	0.65045200
H	-0.40403300	-5.32481700	1.83214000
H	-2.01776300	-5.83801900	2.32935200
H	0.99559600	-2.16579000	0.66201800
C	-1.37991400	3.01082400	-1.80522900
C	-0.24755200	3.24530900	-0.80503300
H	-0.27381600	2.43417900	-0.06929700
H	-1.36914000	3.83307100	-2.53107600
N	1.05018200	3.10629700	-1.50988300
H	1.06405200	3.72641100	-2.31877200
H	1.77521100	3.47302400	-0.89725200
N	-1.12999300	1.73218200	-2.51165700
H	-1.80977700	1.61557300	-3.25716300
H	-0.20755000	1.73427800	-2.93628400
C	-2.76908900	2.93312700	-1.19817000
C	-3.85438200	2.88791300	-2.07187200

C	-3.00748400	2.80387200	0.16627900
C	-5.14194400	2.67526700	-1.60379300
H	-3.69422100	3.02687700	-3.13670900
C	-4.29889700	2.60294200	0.63888000
H	-2.19464800	2.86740400	0.87749800
C	-5.36658900	2.52168500	-0.24251800
H	-5.97060100	2.63802000	-2.29840600
H	-4.47061900	2.52429000	1.70568700
H	-6.36974900	2.35311300	0.12685800
C	-0.34963900	4.56182700	-0.06310700
C	-0.03279100	4.59736100	1.29209900
C	-0.68136200	5.75149600	-0.70645500
C	-0.05138900	5.79253700	1.99639700
H	0.22602400	3.67568400	1.80420500
C	-0.70268700	6.94788500	-0.00425500
H	-0.92946000	5.76115400	-1.76084300
C	-0.38892400	6.97150000	1.34763400
H	0.19337200	5.80346700	3.05027600
H	-0.96801200	7.86424100	-0.51446700
H	-0.40912400	7.90551200	1.89280000

7. NCI plots

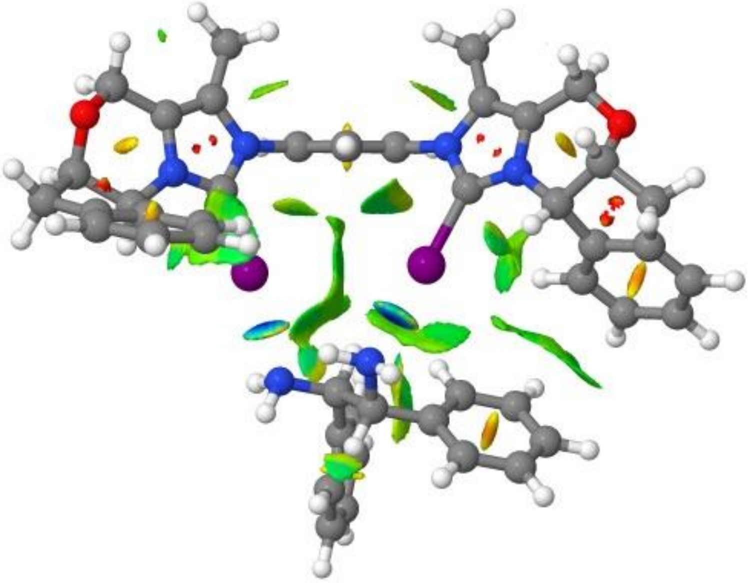

1b with **(1*R*,2*R*)-11**

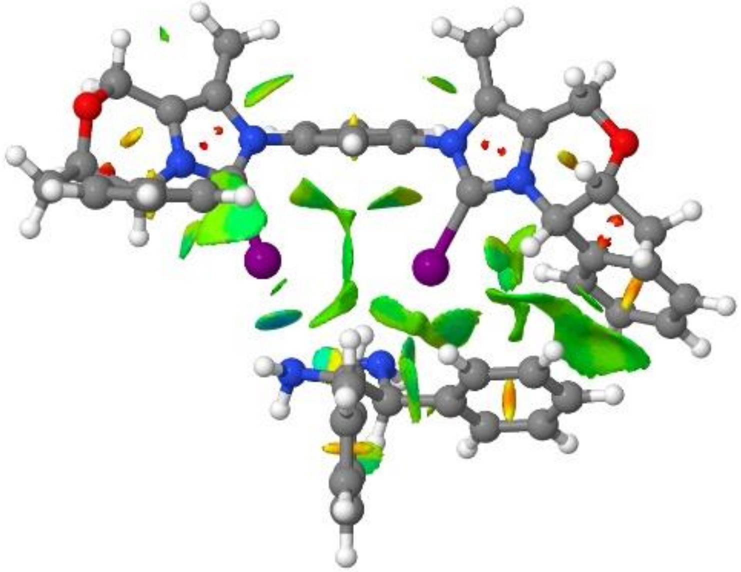

1b with **(1*S*,2*S*)-11**

8. Single crystal X-ray data

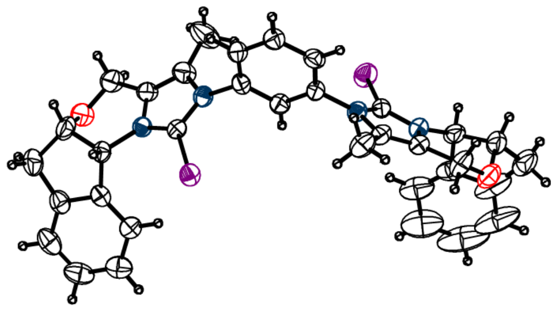

Figure S14. X-ray structure of **1b.OTf** (anions are omitted for clarity).

Table 1. Crystal data and structure refinement for **1b.OTf**

Compound	**1b.OTf**
CCDC-Number	1969509
Empirical formula	C36 H30 F6 I2 N4 O8 S2
Formula weight [g/mol]	1078.56
Crystal system	Triclinic
Space group	P1 (1)
Lattice parameters [Å]	
a	7.9975(7)
b	9.8582(7)
c	13.7497(13)
α	105.006(7)
β	102.361(8)
γ	92.069(7)
Density [g/cm3]	1.759
Crystal size [mm3]	0.273 x 0.186 x 0.172
Volume [Å3]	1017.98(16)
Z	1
Temperature [K]	300(2)
Diffraction Device	XtaLAB Mini (ROW)
Radiation Type	0.71073 Å (Mo K/ fine-focus sealed X-ray tube)
F(000)	530
Absorption coefficient [mm-1]	1.729

Absorption correction	Gaussian
Measurement range	3.0 - 26
Index range	-9 < h < 9
	-12 < k < 12
	-16 < l < 16
Measured reflexes	12883
Independent	7602
Observed	7174
R(int)	0.0183
Completeness (%) / theta (°)	99.6 / 25.242
Transmission (min / max)	0.747 / 0.896
R1 (observed/all)	0.0304 / 0.0337
wR2 (observed/all)	0.0763 / 0.0788
GooF = S	1.05
Rest electron density max./min. [e-/Å^3]	-0.327 / 0.717

Table 2. Atomic coordinates and equivalent isotropic displacement parameters [Å^2] for **1b.OTf**

	x	y	z	U(eq)		x	y	z	U(eq)
I(1)	-0.06516(3)	0.66258(3)	-0.25389(2)	0.04140(12)	F(2)	0.3507(15)	0.2848(8)	0.4960(7)	0.156(4)
I(2)	0.05982(5)	0.67868(5)	0.28789(3)	0.05702(16)	F(3)	0.1036(16)	0.2992(12)	0.5158(7)	0.200(6)
O(1)	-0.5709(6)	1.0420(5)	-0.1952(4)	0.0534(12)	O(3)	0.4643(11)	0.5691(10)	0.6110(9)	0.144(5)
O(2)	0.7019(6)	0.4577(7)	0.2803(4)	0.0664(15)	O(4)	0.1801(11)	0.6014(10)	0.6279(7)	0.110(3)
N(1)	-0.2842(7)	0.6962(5)	-0.0970(4)	0.0393(11)	O(5)	0.3411(14)	0.4463(11)	0.7091(7)	0.127(4)
N(2)	-0.3548(8)	0.8354(6)	-0.1945(5)	0.0357(13)	C(35)	0.2377(12)	0.3712(9)	0.5126(7)	0.065(2)
N(3)	0.1681(6)	0.4916(5)	0.1017(4)	0.0374(11)	S(2)	0.1430(5)	0.0130(2)	0.0995(2)	0.0779(7)
N(4)	0.3723(8)	0.5336(7)	0.2404(5)	0.0451(15)	F(4)	-0.095(3)	0.001(3)	0.1873(19)	0.302(13)
C(1)	-0.0581(8)	0.5978(6)	0.0051(5)	0.0396(13)	F(5)	0.114(3)	0.0847(14)	0.2881(8)	0.231(9)
C(2)	-0.2160(8)	0.5786(6)	-0.0636(5)	0.0384(12)	F(6)	0.019(3)	0.2207(14)	0.2029(10)	0.250(9)
C(3)	-0.3107(9)	0.4492(7)	-0.1030(5)	0.0404(13)	O(6)	0.280(3)	0.1122(15)	0.1160(17)	0.242(9)
C(4)	-0.2479(9)	0.3353(7)	-0.0725(5)	0.0421(14)	O(7)	0.025(2)	0.0097(16)	0.0103(9)	0.189(7)
C(5)	-0.0892(9)	0.3495(7)	-0.0029(6)	0.0430(15)	O(8)	0.1891(11)	-0.1172(7)	0.1167(6)	0.090(2)
C(6)	0.0007(8)	0.4804(7)	0.0334(5)	0.0374(13)	C(36)	0.053(5)	0.0929(19)	0.1988(16)	0.201(13)
C(7)	-0.2501(9)	0.7386(7)	-0.1762(6)	0.0368(15)	H(1)	0.005629	0.685531	0.031118	0.048
C(8)	-0.4166(9)	0.7696(7)	-0.0630(6)	0.0499(16)	H(3)	-0.416305	0.438959	-0.149777	0.049
C(9)	-0.4837(15)	0.7458(11)	0.0232(9)	0.082(3)	H(4)	-0.311802	0.247661	-0.098618	0.051
C(10)	-0.4575(10)	0.8540(8)	-0.1239(7)	0.0429(18)	H(5)	-0.045967	0.27277	0.018131	0.052
C(11)	-0.5949(12)	0.9552(10)	-0.1300(8)	0.056(2)	H(9A)	-0.551556	0.656295	0.000993	0.123
C(12)	-0.5567(9)	0.9600(7)	-0.2952(6)	0.0518(17)	H(9B)	-0.553914	0.81947	0.045081	0.123
C(13)	-0.5400(10)	1.0621(9)	-0.3589(7)	0.063(2)	H(9C)	-0.389543	0.746152	0.079861	0.123
C(14)	-0.3506(9)	1.1081(7)	-0.3294(5)	0.0477(15)	H(11A)	-0.707124	0.901924	-0.156767	0.068
C(15)	-0.2630(12)	1.2250(8)	-0.3441(6)	0.060(2)	H(11B)	-0.591591	1.014345	-0.061114	0.068
C(16)	-0.0875(12)	1.2463(8)	-0.3109(8)	0.062(2)	H(12)	-0.653784	0.88768	-0.328546	0.062
C(17)	0.0027(11)	1.1554(8)	-0.2635(6)	0.0556(18)	H(13A)	-0.57784	1.015801	-0.432379	0.076
C(18)	-0.0833(9)	1.0402(7)	-0.2462(5)	0.0440(14)	H(13B)	-0.606187	1.141691	-0.341288	0.076
C(19)	-0.2581(8)	1.0176(6)	-0.2806(5)	0.0388(13)	H(15)	-0.323358	1.286808	-0.375853	0.072
C(20)	-0.3823(8)	0.8967(6)	-0.2840(5)	0.0393(13)	H(16)	-0.028649	1.323126	-0.320446	0.075
C(21)	0.2136(9)	0.5635(8)	0.2042(6)	0.0393(15)	H(17)	0.121944	1.170457	-0.242653	0.067
C(22)	0.3043(8)	0.4167(7)	0.0749(5)	0.0432(14)	H(18)	-0.022593	0.980598	-0.212209	0.053
C(23)	0.2961(10)	0.3306(8)	-0.0311(6)	0.0572(18)	H(20)	-0.38582	0.820932	-0.346917	0.047
C(24)	0.4299(9)	0.4441(8)	0.1620(6)	0.0422(16)	H(23A)	0.2873	0.390434	-0.076709	0.086
C(25)	0.5967(12)	0.3797(11)	0.1876(8)	0.061(2)	H(23B)	0.197398	0.262332	-0.053381	0.086
C(26)	0.6184(10)	0.4865(13)	0.3639(7)	0.072(3)	H(23C)	0.398462	0.282791	-0.032453	0.086
C(27)	0.7509(11)	0.5759(17)	0.4550(7)	0.091(4)	H(25A)	0.57165	0.284509	0.191624	0.073
C(28)	0.7368(12)	0.7194(15)	0.4379(7)	0.092(5)	H(25B)	0.656584	0.373867	0.132541	0.073
C(29)	0.8515(16)	0.843(2)	0.4731(9)	0.128(7)	H(26)	0.574003	0.400509	0.376835	0.086
C(30)	0.818(2)	0.961(2)	0.4407(14)	0.147(9)	H(27A)	0.723658	0.573128	0.519925	0.109
C(31)	0.662(2)	0.9640(13)	0.3703(12)	0.113(5)	H(27B)	0.86505	0.546146	0.454134	0.109
C(32)	0.5414(13)	0.8424(11)	0.3364(8)	0.076(3)	H(29)	0.954306	0.844462	0.520459	0.154
C(33)	0.5792(10)	0.7272(10)	0.3696(6)	0.061(2)	H(30)	0.898039	1.039632	0.465006	0.176
C(34)	0.4782(8)	0.5866(8)	0.3456(5)	0.0511(16)	H(31)	0.638575	1.043337	0.346899	0.135
S(1)	0.3140(3)	0.5158(3)	0.62644(19)	0.0645(6)	H(32)	0.436523	0.842106	0.291242	0.091
F(1)	0.1960(10)	0.4119(7)	0.4283(4)	0.104(2)	H(34)	0.405703	0.590576	0.395234	0.061

Table 3. Anisotropic displacement parameters [Å²] for **1b.OTf**

	U11	U22	U33	U23	U13	U12
I(1)	0.0436(2)	0.0402(2)	0.0434(2)	0.01178(16)	0.01427(18)	0.01481(17)
I(2)	0.0469(3)	0.0774(4)	0.0495(3)	0.0157(3)	0.0175(2)	0.0152(3)
O(1)	0.050(3)	0.048(2)	0.078(3)	0.030(2)	0.026(2)	0.024(2)
O(2)	0.034(2)	0.111(5)	0.057(3)	0.028(3)	0.009(2)	0.011(3)
N(1)	0.036(3)	0.039(3)	0.046(3)	0.015(2)	0.011(2)	0.011(2)
N(2)	0.035(3)	0.035(3)	0.039(3)	0.012(2)	0.010(2)	0.008(2)
N(3)	0.034(3)	0.036(2)	0.041(3)	0.010(2)	0.005(2)	0.002(2)
N(4)	0.033(3)	0.058(4)	0.047(3)	0.022(3)	0.006(3)	0.005(3)
C(1)	0.040(3)	0.034(3)	0.043(3)	0.009(2)	0.008(3)	0.002(2)
C(2)	0.043(3)	0.040(3)	0.037(3)	0.016(2)	0.011(2)	0.010(3)
C(3)	0.040(3)	0.045(3)	0.035(3)	0.011(2)	0.006(3)	0.002(3)
C(4)	0.045(4)	0.034(3)	0.047(3)	0.010(3)	0.011(3)	0.004(3)
C(5)	0.043(4)	0.039(3)	0.055(4)	0.022(3)	0.018(3)	0.008(3)
C(6)	0.032(3)	0.043(3)	0.037(3)	0.013(2)	0.005(2)	0.005(2)
C(7)	0.032(3)	0.040(3)	0.040(4)	0.012(3)	0.009(3)	0.006(3)
C(8)	0.052(4)	0.041(3)	0.069(4)	0.024(3)	0.026(3)	0.016(3)
C(9)	0.104(7)	0.087(6)	0.097(7)	0.053(6)	0.071(6)	0.048(6)
C(10)	0.038(4)	0.038(4)	0.063(5)	0.023(3)	0.019(3)	0.012(3)
C(11)	0.048(4)	0.055(4)	0.080(6)	0.030(4)	0.027(4)	0.022(4)
C(12)	0.035(3)	0.046(4)	0.071(5)	0.023(3)	-0.003(3)	0.007(3)
C(13)	0.061(5)	0.067(5)	0.070(5)	0.037(4)	0.009(4)	0.023(4)
C(14)	0.056(4)	0.045(3)	0.047(4)	0.018(3)	0.014(3)	0.015(3)
C(15)	0.087(6)	0.045(4)	0.059(4)	0.024(3)	0.024(4)	0.020(4)
C(16)	0.080(6)	0.036(4)	0.080(6)	0.015(4)	0.038(5)	0.001(3)
C(17)	0.062(4)	0.051(4)	0.053(4)	0.006(3)	0.022(3)	-0.001(3)
C(18)	0.044(4)	0.041(3)	0.045(3)	0.007(3)	0.013(3)	0.008(3)
C(19)	0.049(3)	0.034(3)	0.036(3)	0.010(2)	0.014(3)	0.011(2)
C(20)	0.039(3)	0.037(3)	0.042(3)	0.013(2)	0.004(2)	0.008(2)
C(21)	0.034(3)	0.051(4)	0.038(3)	0.019(3)	0.011(3)	0.009(3)
C(22)	0.033(3)	0.043(3)	0.051(4)	0.011(3)	0.006(3)	0.001(3)
C(23)	0.046(4)	0.059(4)	0.058(4)	0.003(3)	0.011(3)	0.007(3)
C(24)	0.035(3)	0.049(4)	0.045(4)	0.016(3)	0.009(3)	0.004(3)
C(25)	0.037(4)	0.073(5)	0.070(5)	0.015(4)	0.008(4)	0.016(4)
C(26)	0.043(4)	0.130(9)	0.059(5)	0.054(5)	0.009(3)	0.014(5)
C(27)	0.044(5)	0.186(13)	0.048(5)	0.045(6)	0.006(4)	0.008(6)
C(28)	0.048(5)	0.160(12)	0.037(4)	-0.030(6)	0.018(4)	-0.025(6)
C(29)	0.068(7)	0.203(17)	0.061(6)	-0.054(9)	0.024(5)	-0.061(10)
C(30)	0.118(13)	0.152(15)	0.118(12)	-0.068(12)	0.060(10)	-0.083(13)
C(31)	0.116(11)	0.082(8)	0.127(11)	-0.013(7)	0.054(9)	-0.024(7)
C(32)	0.069(6)	0.077(6)	0.067(5)	-0.015(5)	0.029(5)	-0.021(5)
C(33)	0.054(4)	0.085(6)	0.039(4)	0.001(4)	0.021(3)	-0.015(4)
C(34)	0.035(3)	0.082(5)	0.037(3)	0.019(3)	0.009(3)	-0.002(3)
S(1)	0.0420(10)	0.0683(13)	0.0730(13)	0.0004(11)	0.0149(9)	0.0052(9)
F(1)	0.140(6)	0.108(4)	0.066(3)	0.039(3)	0.013(3)	0.013(4)
F(2)	0.220(10)	0.083(4)	0.120(6)	-0.009(4)	-0.018(6)	0.067(6)
F(3)	0.238(12)	0.215(10)	0.103(5)	-0.022(6)	0.060(6)	-0.169(10)
O(3)	0.091(6)	0.118(7)	0.184(10)	-0.051(7)	0.073(6)	-0.046(5)
O(4)	0.094(6)	0.130(7)	0.110(7)	0.018(6)	0.041(5)	0.058(5)
O(5)	0.149(9)	0.133(8)	0.071(5)	0.018(5)	-0.023(5)	0.029(7)
C(35)	0.077(5)	0.059(4)	0.062(5)	0.028(4)	0.011(4)	-0.006(4)
S(2)	0.119(2)	0.0529(12)	0.0732(16)	0.0174(11)	0.0459(17)	0.0101(13)
F(4)	0.28(2)	0.44(3)	0.35(3)	0.24(3)	0.25(2)	0.20(2)
F(5)	0.47(3)	0.159(10)	0.082(6)	0.038(6)	0.090(11)	0.118(13)
F(6)	0.49(3)	0.180(10)	0.146(9)	0.075(8)	0.153(13)	0.218(15)
O(6)	0.29(2)	0.126(10)	0.34(2)	0.054(12)	0.148(18)	-0.079(12)

O(7)	0.29(2)	0.186(13)	0.083(7)	0.026(7)	0.026(9)	0.090(13)
O(8)	0.090(5)	0.064(4)	0.117(6)	0.015(4)	0.039(5)	0.007(4)
C(36)	0.44(4)	0.130(13)	0.132(14)	0.082(11)	0.19(2)	0.17(2)

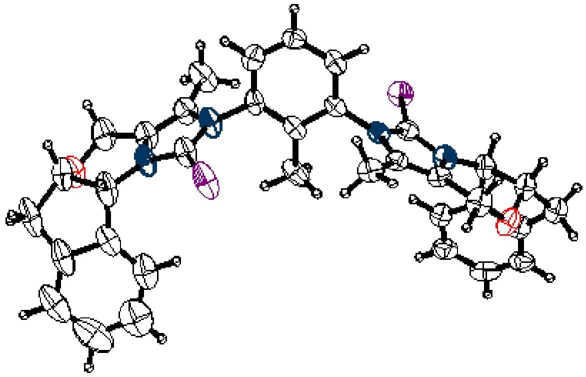

Figure S15. X-ray structure of *anti*-**1c.OTf** (anions are omitted for clarity).

Table 4. Crystal data and structure refinement for *anti*-**1c.OTf**

Compound	*anti*-**1c.OTf**
CCDC-Number	1970644
Empirical formula	C37 H32 F6 I2 N4 O8 S2
Formula weight [g/mol]	1092.58
Crystal system	Monoclinic
Space group	I2 (5)
Lattice parameters [Å]	
a	19.1690(4)
b	9.9770(2)
c	23.2626(4)
α	90
β	110.995(2)
γ	90
Density [g/cm^3]	1.747
Crystal size [mm^3]	0.262 x 0.041 x 0.039
Volume [Å^3]	4153.59(15)
Z	4
Temperature [K]	169.99(10)
Diffraction Device	XtaLAB Synergy, Dualflex, HyPix
Radiation Type	1.54184 Å (Cu K/ micro-focus sealed X-ray tube)
F(000)	2152
Absorption coefficient [mm^{-1}]	13.559
Absorption correction	Gaussian
Measurement range	2.6 - 66.5
Index range	-22 < h < 22
	-11 < k < 11
	-27 < l < 27
Measured reflexes	23732
Independent	7291
Observed	6956

R(int)	0.0439
Completeness (%) / theta (°)	99.9 / 66.492
Transmission (min / max)	0.258 / 0.673
R1 (observed/all)	0.0511 / 0.0530
wR2 (observed/all)	0.1390 / 0.1407
GooF = S	1.033
Rest electron density max./min. [e-/Å^3]	-1.219 / 0.853

Table 5. Atomic coordinates and equivalent isotropic displacement parameters [Å^2] for 107squeeze

	x	y	z	U(eq)		x	y	z	U(eq)	S.O.F
I(1)	0.44873(4)	0.62046(7)	0.07052(3)	0.0488(2)	C(37)	0.5033(9)	0.288(2)	0.3736(9)	0.0868(11)	1
I(2)	0.52758(4)	0.79885(13)	0.41029(3)	0.0725(3)	H(2A)	0.63643	0.570118	0.330987	0.071	1
O(1)	0.6647(4)	0.2223(8)	0.1818(4)	0.0506(18)	H(2AB)	0.67464	0.68839	0.306277	0.071	1
O(2)	0.2258(5)	0.9326(11)	0.3663(4)	0.064(2)	H(2AC)	0.71397	0.544566	0.321412	0.071	1
N(1)	0.3639(5)	0.8832(11)	0.3608(4)	0.049(2)	H(5A)	0.73554	0.374654	0.212333	0.068	1
N(2)	0.4093(5)	0.8783(11)	0.2883(4)	0.048(2)	H(5AB)	0.70962	0.308307	0.26421	0.068	1
N(3)	0.5638(4)	0.6184(10)	0.2019(3)	0.0331(14)	H(6)	0.67799	0.306179	0.107101	0.059	1
N(4)	0.5772(5)	0.4462(9)	0.1509(4)	0.0412(18)	H(7A)	0.6089	0.145861	0.037445	0.063	1
C(1)	0.5360(6)	0.5594(10)	0.1476(4)	0.037(2)	H(7AB)	0.64128	0.055404	0.098865	0.063	1
C(2)	0.6659(7)	0.5915(10)	0.3054(5)	0.047(3)	H(10)	0.55716	0.392249	0.062615	0.05	1
C(3)	0.6246(5)	0.5482(10)	0.2411(5)	0.037(2)	H(11)	0.49891	-0.07472	0.059482	0.059	1
C(4)	0.6323(6)	0.4393(10)	0.2084(4)	0.040(2)	H(12)	0.38535	-0.05675	0.069304	0.07	1
C(5)	0.6924(7)	0.3361(13)	0.2205(5)	0.056(3)	H(13)	0.34786	0.143996	0.099771	0.062	1
C(6)	0.6383(7)	0.2601(12)	0.1183(5)	0.050(3)	H(14)	0.42405	0.332678	0.115833	0.053	1
C(7)	0.6101(7)	0.1342(14)	0.0800(6)	0.053(3)	H(15A)	0.24655	0.96132	0.183486	0.104	1
C(8)	0.5318(6)	0.1204(15)	0.0814(4)	0.047(2)	H(15B)	0.31355	0.888217	0.170206	0.104	1
C(9)	0.5074(6)	0.2416(11)	0.0975(5)	0.044(2)	H(15C)	0.31991	1.043864	0.187974	0.104	1
C(10)	0.5678(6)	0.3467(11)	0.1032(4)	0.041(2)	H(20A)	0.23361	1.071352	0.306729	0.079	1
C(11)	0.4838(8)	0.0087(12)	0.0710(5)	0.049(3)	H(20B)	0.19261	0.936603	0.274312	0.079	1
C(12)	0.4174(8)	0.0191(13)	0.0771(6)	0.058(3)	H(21)	0.3011	1.06778	0.421771	0.079	1
C(13)	0.3947(7)	0.1385(13)	0.0945(5)	0.051(3)	H(22)	0.40354	0.923687	0.452073	0.072	1
C(14)	0.4400(6)	0.2500(11)	0.1043(5)	0.044(2)	H(24)	0.39278	0.604996	0.415166	0.084	1
C(15)	0.3009(7)	0.9566(19)	0.1951(6)	0.069(4)	H(25)	0.36002	0.434484	0.467032	0.112	1
C(16)	0.3356(6)	0.9209(13)	0.2615(6)	0.052(3)	H(26)	0.2914	0.468938	0.527591	0.125	1
C(17)	0.3074(6)	0.9206(14)	0.3075(5)	0.053(3)	H(27)	0.25221	0.691429	0.539824	0.099	1
C(19)	0.4258(6)	0.8596(14)	0.3486(5)	0.052(3)	H(29A)	0.30385	0.967393	0.513347	0.097	1
C(20)	0.2346(7)	0.9723(17)	0.3098(7)	0.066(3)	H(29B)	0.22074	0.9255	0.469425	0.097	1
C(21)	0.2903(8)	0.9695(18)	0.4195(7)	0.066(3)	H(30A)	0.41803	0.632978	0.276557	0.093	1
C(22)	0.3572(7)	0.8837(16)	0.4216(6)	0.060(3)	H(30B)	0.48056	0.548322	0.261851	0.093	1
C(23)	0.3420(6)	0.7523(14)	0.4462(5)	0.052(3)	H(30C)	0.40666	0.595166	0.206919	0.093	1
C(24)	0.3642(7)	0.624(2)	0.4402(6)	0.070(3)	H(33)	0.48357	1.072551	0.262382	0.056	1
C(25)	0.3443(11)	0.523(3)	0.4712(8)	0.093(6)	H(34)	0.5782	1.062807	0.22179	0.054	1
C(26)	0.3038(12)	0.542(3)	0.5070(9)	0.105(7)	H(35)	0.61149	0.860846	0.191727	0.049	1
C(27)	0.2796(8)	0.674(3)	0.5137(7)	0.083(5)	S(2A)	0.18783(19)	0.2057(3)	0.58772(13)	0.0567(7)	0.614(8)
C(28)	0.2969(7)	0.7755(19)	0.4819(6)	0.068(4)	F(1A)	0.3127(13)	0.0867(18)	0.6260(11)	0.129(4)	0.614(8)
C(29)	0.2745(8)	0.917(2)	0.4759(7)	0.081(5)	F(2A)	0.2972(13)	0.195(2)	0.5493(9)	0.129(4)	0.614(8)
C(30)	0.4434(7)	0.6199(18)	0.2471(6)	0.062(3)	F(3A)	0.3204(12)	0.296(2)	0.6323(10)	0.129(4)	0.614(8)
C(31)	0.4810(5)	0.7454(11)	0.2412(4)	0.039(2)	O(6A)	0.1819(12)	0.222(2)	0.6454(8)	0.105(4)	0.614(8)
C(32)	0.4647(6)	0.8699(11)	0.2591(5)	0.042(2)	O(7A)	0.1615(13)	0.0729(18)	0.5640(9)	0.105(4)	0.614(8)
C(33)	0.4985(6)	0.9889(11)	0.2510(5)	0.046(2)	O(8A)	0.1477(11)	0.299(2)	0.5414(8)	0.105(4)	0.614(8)
C(34)	0.5538(6)	0.9830(11)	0.2265(5)	0.045(2)	C(38A)	0.2828(11)	0.2004(18)	0.5998(7)	0.0567(7)	0.614(8)
C(35)	0.5736(6)	0.8643(11)	0.2091(5)	0.040(2)	S(2B)	0.18783(19)	0.2057(3)	0.58772(13)	0.0567(7)	0.386(8)
C(36)	0.5384(5)	0.7467(10)	0.2165(4)	0.0341(19)	F(1B)	0.2744(19)	0.088(3)	0.6839(13)	0.129(4)	0.386(8)
S(1)	0.4067(3)	0.2842(5)	0.3432(2)	0.0868(11)	F(2B)	0.266(2)	-0.012(3)	0.6039(16)	0.129(4)	0.386(8)
F(4)	0.5181(15)	0.360(2)	0.4325(16)	0.246(9)	F(3B)	0.3299(19)	0.158(3)	0.6294(18)	0.129(4)	0.386(8)
F(5)	0.5328(14)	0.354(2)	0.3446(15)	0.246(9)	O(6B)	0.2041(18)	0.327(3)	0.6219(13)	0.105(4)	0.386(8)
F(6)	0.5345(14)	0.175(2)	0.3918(15)	0.246(9)	O(7B)	0.1284(16)	0.135(4)	0.5934(13)	0.105(4)	0.386(8)
O(3)	0.3853(7)	0.4253(12)	0.3361(6)	0.086(3)	O(8B)	0.1850(19)	0.224(4)	0.5264(10)	0.105(4)	0.386(8)
O(4)	0.3982(13)	0.223(3)	0.2892(9)	0.203(13)	C(38B)	0.2660(14)	0.107(3)	0.6267(11)	0.0567(7)	0.386(8)
O(5)	0.3814(19)	0.219(2)	0.3841(16)	0.219(14)						

Table 6. Anisotropic displacement parameters [Å^2] for **107squeeze**

	U11	U22	U33	U23	U13	U12
I(1)	0.0456(4)	0.0606(4)	0.0302(3)	0.0038(3)	0.0014(3)	0.0070(3)
I(2)	0.0335(4)	0.1463(9)	0.0343(4)	0.0035(4)	0.0080(3)	0.0213(4)
O(1)	0.045(4)	0.050(4)	0.045(4)	-0.006(3)	0.002(3)	0.013(3)
O(2)	0.037(4)	0.102(7)	0.053(5)	0.003(5)	0.015(4)	0.016(4)
N(1)	0.025(4)	0.085(7)	0.041(5)	-0.003(4)	0.015(4)	0.009(4)
N(2)	0.030(4)	0.081(6)	0.032(4)	-0.003(4)	0.009(4)	0.007(4)
N(3)	0.029(4)	0.043(3)	0.026(3)	0.004(4)	0.007(3)	-0.001(4)
N(4)	0.038(4)	0.048(5)	0.032(4)	-0.002(4)	0.006(3)	0.003(4)
C(1)	0.037(5)	0.043(5)	0.030(5)	0.005(4)	0.008(4)	-0.001(4)
C(2)	0.049(6)	0.049(7)	0.034(5)	-0.003(4)	0.003(5)	-0.005(4)
C(3)	0.030(5)	0.043(5)	0.035(5)	0.008(4)	0.009(4)	0.003(4)
C(4)	0.037(5)	0.045(5)	0.028(5)	0.006(4)	-0.001(4)	0.003(4)
C(5)	0.041(6)	0.070(8)	0.042(6)	-0.004(5)	-0.003(5)	0.011(5)
C(6)	0.044(6)	0.059(6)	0.044(6)	-0.008(5)	0.013(5)	0.011(5)
C(7)	0.053(6)	0.052(6)	0.052(6)	-0.004(6)	0.017(5)	0.003(6)
C(8)	0.050(6)	0.051(5)	0.036(5)	0.002(6)	0.010(4)	0.008(6)
C(9)	0.044(6)	0.050(6)	0.031(5)	-0.007(4)	0.006(4)	0.000(5)
C(10)	0.037(5)	0.054(6)	0.029(5)	-0.004(4)	0.006(4)	0.004(4)
C(11)	0.064(7)	0.044(6)	0.036(6)	0.003(4)	0.015(5)	-0.004(5)
C(12)	0.068(8)	0.047(6)	0.046(6)	0.011(5)	0.004(6)	-0.012(6)
C(13)	0.053(6)	0.053(7)	0.047(6)	0.011(5)	0.016(5)	0.003(5)
C(14)	0.044(6)	0.049(6)	0.034(5)	0.001(4)	0.007(4)	0.007(4)
C(15)	0.036(6)	0.119(12)	0.051(7)	0.022(8)	0.014(5)	0.016(7)
C(16)	0.030(5)	0.073(8)	0.049(6)	0.007(5)	0.011(5)	0.012(5)
C(17)	0.036(6)	0.077(8)	0.043(6)	-0.003(5)	0.011(5)	0.006(5)
C(19)	0.030(5)	0.083(8)	0.039(6)	-0.002(5)	0.009(4)	0.009(5)
C(20)	0.036(6)	0.095(10)	0.067(8)	-0.004(7)	0.019(6)	0.010(6)
C(21)	0.052(7)	0.090(9)	0.063(7)	-0.023(7)	0.030(6)	0.004(6)
C(22)	0.033(6)	0.102(10)	0.043(6)	-0.018(6)	0.011(5)	0.004(6)
C(23)	0.032(5)	0.088(9)	0.033(5)	-0.010(5)	0.009(4)	-0.011(5)
C(24)	0.048(7)	0.111(10)	0.047(6)	-0.012(9)	0.012(5)	0.010(9)
C(25)	0.073(11)	0.131(16)	0.063(10)	-0.001(10)	0.009(8)	0.006(10)
C(26)	0.080(12)	0.16(2)	0.064(10)	0.017(12)	0.018(9)	-0.030(14)
C(27)	0.043(7)	0.162(18)	0.041(7)	0.001(8)	0.013(6)	-0.009(9)
C(28)	0.035(6)	0.128(13)	0.042(6)	-0.017(7)	0.015(5)	0.000(7)
C(29)	0.048(7)	0.153(16)	0.049(7)	-0.010(9)	0.027(6)	0.014(9)
C(30)	0.047(6)	0.085(8)	0.062(7)	-0.002(8)	0.030(6)	-0.025(7)
C(31)	0.027(4)	0.060(6)	0.029(4)	0.002(4)	0.007(4)	-0.004(4)
C(32)	0.038(5)	0.059(6)	0.030(5)	-0.005(4)	0.013(4)	0.006(4)
C(33)	0.043(6)	0.049(6)	0.041(5)	-0.008(4)	0.007(4)	0.003(4)
C(34)	0.048(6)	0.045(6)	0.040(5)	0.001(4)	0.014(5)	-0.005(5)
C(35)	0.035(5)	0.053(6)	0.034(5)	-0.008(4)	0.013(4)	-0.012(4)
C(36)	0.028(4)	0.047(5)	0.024(4)	0.000(4)	0.006(3)	-0.006(4)
S(2A)	0.0578(17)	0.0736(19)	0.0398(14)	-0.0035(13)	0.0189(13)	0.0087(14)
F(1A)	0.111(8)	0.130(9)	0.162(9)	-0.038(8)	0.069(7)	-0.023(7)
F(2A)	0.111(8)	0.130(9)	0.162(9)	-0.038(8)	0.069(7)	-0.023(7)
F(3A)	0.111(8)	0.130(9)	0.162(9)	-0.038(8)	0.069(7)	-0.023(7)
O(6A)	0.083(7)	0.152(11)	0.082(7)	-0.031(7)	0.032(5)	-0.012(7)
O(7A)	0.083(7)	0.152(11)	0.082(7)	-0.031(7)	0.032(5)	-0.012(7)
O(8A)	0.083(7)	0.152(11)	0.082(7)	-0.031(7)	0.032(5)	-0.012(7)
C(38A)	0.0578(17)	0.0736(19)	0.0398(14)	-0.0035(13)	0.0189(13)	0.0087(14)
S(2B)	0.0578(17)	0.0736(19)	0.0398(14)	-0.0035(13)	0.0189(13)	0.0087(14)
F(1B)	0.111(8)	0.130(9)	0.162(9)	-0.038(8)	0.069(7)	-0.023(7)
F(2B)	0.111(8)	0.130(9)	0.162(9)	-0.038(8)	0.069(7)	-0.023(7)
F(3B)	0.111(8)	0.130(9)	0.162(9)	-0.038(8)	0.069(7)	-0.023(7)
O(6B)	0.083(7)	0.152(11)	0.082(7)	-0.031(7)	0.032(5)	-0.012(7)
O(7B)	0.083(7)	0.152(11)	0.082(7)	-0.031(7)	0.032(5)	-0.012(7)
O(8B)	0.083(7)	0.152(11)	0.082(7)	-0.031(7)	0.032(5)	-0.012(7)
C(38B)	0.0578(17)	0.0736(19)	0.0398(14)	-0.0035(13)	0.0189(13)	0.0087(14)
S(1)	0.088(3)	0.079(2)	0.088(3)	-0.001(2)	0.026(2)	0.000(2)
F(4)	0.173(11)	0.166(10)	0.33(2)	0.107(13)	0.004(13)	0.026(8)
F(5)	0.173(11)	0.166(10)	0.33(2)	0.107(13)	0.004(13)	0.026(8)
F(6)	0.173(11)	0.166(10)	0.33(2)	0.107(13)	0.004(13)	0.026(8)
O(3)	0.088(8)	0.082(7)	0.095(8)	-0.003(6)	0.041(7)	0.012(6)
O(4)	0.19(2)	0.23(2)	0.123(14)	-0.084(15)	-0.028(13)	0.134(18)
O(5)	0.28(4)	0.131(16)	0.33(4)	0.01(2)	0.21(3)	-0.049(19)
C(37)	0.088(3)	0.079(2)	0.088(3)	-0.001(2)	0.026(2)	0.000(2)

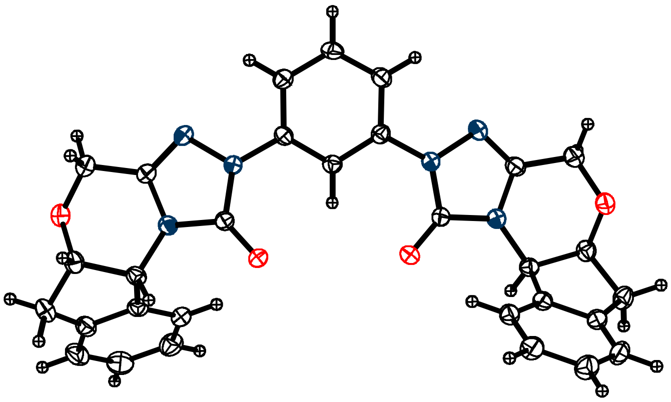

Figure S16. X-ray structure of **5**.

Table 8. Crystal data and structure refinement for **5**

Compound	**5**
CCDC-Number	1969510
Empirical formula	C30 H24 N6 O4
Formula weight [g/mol]	532.55
Crystal system	Monoclinic
Space group	I2 (5)
Lattice parameters [Å]	
a	14.4836(4)
b	7.5608(2)
c	23.2791(6)
α	90
β	94.443(2)
γ	90
Density [g/cm^3]	1.392
Crystal size [mm^3]	0.243 x 0.198 x 0.092
Volume [Å^3]	2541.57(11)
Z	4
Temperature [K]	170.00(10)
Diffraction Device	SuperNova, Single source at offset/far, Atlas
Radiation Type	1.54184 Å (Cu K/ micro-focus sealed X-ray tube)
F(000)	1112
Absorption coefficient [mm^{-1}]	0.782
Absorption correction	Gaussian
Measurement range	3.5 - 66.5
Index range	-17 < h < 17
	-6 < k < 8
	-27 < l < 27
Measured reflexes	8144
Independent	3715

Observed	3612
R(int)	0.0212
Completeness (%) / theta (°)	100.0 / 66.496
Transmission (min / max)	0.665 / 1.000
R1 (observed/all)	0.0268 / 0.0279
wR2 (observed/all)	0.0669 / 0.0681
GooF = S	1.073
Rest electron density max./min. [e-/Å³]	-0.172 / 0.19

Table 9. Atomic coordinates and equivalent isotropic displacement parameters [Å^2] for **5**

	x	y	z	U(eq)		x	y	z	U(eq)
N(1)	0.61168(10)	0.3510(2)	0.48110(6)	0.0214(3)	C(23)	0.64132(12)	0.7026(3)	0.17048(8)	0.0218(4)
O(1)	0.59695(10)	0.6530(2)	0.45676(6)	0.0275(3)	C(24)	0.55249(13)	0.8072(3)	0.15984(8)	0.0220(4)
O(3)	0.67148(9)	0.5037(2)	0.66368(5)	0.0262(3)	C(25)	0.48469(13)	0.8442(3)	0.19714(8)	0.0248(4)
N(3)	0.62030(10)	0.5413(2)	0.55046(7)	0.0220(3)	C(26)	0.40944(14)	0.9464(3)	0.17683(9)	0.0308(5)
N(5)	0.63108(12)	0.2537(2)	0.22237(7)	0.0276(4)	C(27)	0.40254(14)	1.0106(3)	0.12093(9)	0.0334(5)
C(1)	0.60493(12)	0.2605(3)	0.42757(8)	0.0201(4)	C(28)	0.47064(14)	0.9734(3)	0.08382(9)	0.0306(5)
C(2)	0.59079(13)	0.0789(3)	0.42720(9)	0.0239(4)	C(29)	0.54560(12)	0.8704(3)	0.10344(8)	0.0243(4)
N(2)	0.62415(11)	0.2536(2)	0.53229(7)	0.0249(4)	C(30)	0.62702(14)	0.8079(3)	0.07175(9)	0.0290(5)
O(2)	0.62415(10)	0.6544(2)	0.29705(6)	0.0307(3)	H(2)	0.582807	0.01673	0.461934	0.029
C(3)	0.58860(13)	-0.0097(3)	0.37494(8)	0.0256(4)	H(3)	0.579478	-0.134129	0.374234	0.031
C(4)	0.59940(13)	0.0788(3)	0.32360(9)	0.0250(4)	H(4)	0.597641	0.016477	0.288102	0.03
O(4)	0.61532(9)	0.4990(2)	0.08963(6)	0.0280(3)	H(6)	0.626297	0.479276	0.377805	0.026
N(4)	0.62512(11)	0.3518(2)	0.27301(7)	0.0232(3)	H(9A)	0.578407	0.312016	0.649592	0.032
C(5)	0.61285(12)	0.2608(3)	0.32540(8)	0.0216(4)	H(9AB)	0.683236	0.248244	0.645163	0.032
C(6)	0.61641(12)	0.3551(3)	0.37702(7)	0.0216(4)	H(10)	0.566394	0.778816	0.571094	0.027
N(6)	0.63483(10)	0.5413(2)	0.20442(7)	0.0225(3)	H(11)	0.549408	0.638058	0.658827	0.03
C(7)	0.60838(11)	0.5305(3)	0.49118(8)	0.0209(4)	H(12A)	0.616704	0.906099	0.687194	0.035
C(8)	0.62875(12)	0.3738(3)	0.57202(8)	0.0217(4)	H(12B)	0.691397	0.772927	0.718797	0.035
C(9)	0.63892(14)	0.3456(3)	0.63577(8)	0.0268(4)	H(14)	0.829911	1.007776	0.69096	0.04
C(10)	0.62046(12)	0.7033(3)	0.58461(8)	0.0223(4)	H(15)	0.925026	1.067645	0.616307	0.045
C(11)	0.61475(12)	0.6549(3)	0.64884(8)	0.0251(4)	H(16)	0.883266	0.969795	0.522891	0.042
C(12)	0.66162(14)	0.8105(3)	0.68105(9)	0.0289(5)	H(17)	0.746494	0.808062	0.502453	0.032
C(13)	0.73296(13)	0.8696(3)	0.64093(8)	0.0262(4)	H(21A)	0.714071	0.325193	0.114395	0.038
C(14)	0.81338(15)	0.9666(3)	0.65299(10)	0.0337(5)	H(21B)	0.612206	0.240708	0.10673	0.038
C(15)	0.86936(15)	1.0025(3)	0.60859(11)	0.0376(5)	H(22)	0.733524	0.639679	0.106337	0.03
C(16)	0.84448(15)	0.9437(3)	0.55288(10)	0.0349(5)	H(23)	0.690827	0.780654	0.188971	0.026
C(17)	0.76372(14)	0.8474(3)	0.54054(8)	0.0269(4)	H(25)	0.489657	0.800695	0.23554	0.03
C(18)	0.70884(13)	0.8102(3)	0.58531(8)	0.0237(4)	H(26)	0.362105	0.972531	0.201585	0.037
C(19)	0.62774(12)	0.5318(3)	0.26334(8)	0.0218(4)	H(27)	0.350723	1.080835	0.107843	0.04
C(20)	0.63738(13)	0.3734(3)	0.18326(8)	0.0243(4)	H(28)	0.465838	1.018009	0.045556	0.037
C(21)	0.64809(15)	0.3458(3)	0.12065(8)	0.0314(5)	H(30A)	0.673766	0.902643	0.069532	0.035
C(22)	0.66530(12)	0.6543(3)	0.10870(8)	0.0247(4)	H(30B)	0.606722	0.767974	0.032275	0.035

Table 10. Anisotropic displacement parameters [Å^2] for **5**

	U11	U22	U33	U23	U13	U12
N(1)	0.0279(8)	0.0188(9)	0.0175(7)	0.0004(7)	0.0019(6)	0.0006(7)
O(1)	0.0409(8)	0.0200(7)	0.0210(7)	0.0022(6)	-0.0016(5)	0.0048(6)
O(3)	0.0277(6)	0.0294(8)	0.0211(6)	0.0001(6)	0.0001(5)	0.0001(6)
N(3)	0.0250(7)	0.0205(9)	0.0201(8)	-0.0012(7)	-0.0008(6)	0.0016(7)
N(5)	0.0402(9)	0.0231(10)	0.0196(8)	-0.0034(7)	0.0041(6)	0.0001(8)
C(1)	0.0188(8)	0.0212(10)	0.0206(9)	-0.0022(8)	0.0034(6)	0.0013(7)
C(2)	0.0256(9)	0.0226(11)	0.0237(9)	0.0016(8)	0.0041(7)	-0.0019(7)
N(2)	0.0325(8)	0.0218(9)	0.0207(8)	0.0040(7)	0.0027(6)	0.0007(7)
O(2)	0.0493(8)	0.0207(8)	0.0222(7)	-0.0033(6)	0.0035(6)	0.0024(7)
C(3)	0.0279(9)	0.0175(10)	0.0315(10)	-0.0022(8)	0.0034(7)	-0.0045(8)
C(4)	0.0279(9)	0.0233(11)	0.0238(10)	-0.0045(8)	0.0021(7)	-0.0023(8)
O(4)	0.0354(7)	0.0274(8)	0.0214(6)	-0.0017(6)	0.0039(5)	-0.0032(6)
N(4)	0.0315(8)	0.0196(9)	0.0184(7)	-0.0009(7)	0.0021(6)	0.0010(7)
C(5)	0.0214(8)	0.0217(10)	0.0217(9)	0.0016(8)	0.0016(7)	0.0015(8)
C(6)	0.0224(8)	0.0192(10)	0.0234(10)	-0.0004(8)	0.0023(7)	0.0015(7)
N(6)	0.0272(7)	0.0209(9)	0.0194(8)	-0.0005(7)	0.0023(6)	0.0010(7)
C(7)	0.0208(8)	0.0211(10)	0.0208(9)	-0.0006(8)	0.0005(6)	0.0018(8)
C(8)	0.0215(8)	0.0214(11)	0.0224(9)	0.0025(8)	0.0025(7)	0.0003(8)
C(9)	0.0316(9)	0.0266(11)	0.0221(9)	0.0030(9)	0.0026(7)	-0.0011(8)
C(10)	0.0215(8)	0.0214(11)	0.0237(9)	-0.0030(8)	0.0009(7)	0.0034(7)
C(11)	0.0225(8)	0.0298(11)	0.0233(9)	-0.0020(9)	0.0038(7)	0.0015(8)
C(12)	0.0308(10)	0.0320(13)	0.0240(9)	-0.0083(9)	0.0034(8)	0.0013(8)
C(13)	0.0281(9)	0.0250(11)	0.0256(9)	-0.0038(9)	0.0020(7)	0.0025(9)
C(14)	0.0360(10)	0.0304(13)	0.0338(11)	-0.0083(9)	-0.0029(9)	-0.0038(9)
C(15)	0.0342(10)	0.0327(13)	0.0455(12)	-0.0021(11)	0.0017(9)	-0.0107(10)
C(16)	0.0378(11)	0.0320(13)	0.0357(11)	0.0058(10)	0.0084(9)	-0.0051(10)
C(17)	0.0339(10)	0.0225(11)	0.0241(9)	0.0027(9)	0.0018(7)	0.0006(9)
C(18)	0.0247(9)	0.0197(10)	0.0266(9)	-0.0006(8)	0.0005(7)	0.0037(8)
C(19)	0.0255(8)	0.0210(10)	0.0187(8)	0.0001(8)	0.0002(6)	0.0017(8)
C(20)	0.0274(9)	0.0219(11)	0.0239(10)	-0.0035(8)	0.0037(7)	-0.0011(8)
C(21)	0.0457(11)	0.0254(12)	0.0240(10)	-0.0029(9)	0.0074(8)	-0.0012(10)
C(22)	0.0241(8)	0.0275(11)	0.0229(9)	0.0000(9)	0.0053(7)	-0.0021(8)
C(23)	0.0224(8)	0.0205(11)	0.0224(9)	-0.0006(8)	0.0019(7)	-0.0030(7)
C(24)	0.0227(8)	0.0205(10)	0.0225(9)	-0.0006(7)	0.0001(7)	-0.0024(7)
C(25)	0.0273(9)	0.0249(11)	0.0224(9)	-0.0016(8)	0.0037(7)	-0.0010(8)
C(26)	0.0299(9)	0.0318(12)	0.0313(11)	0.0018(9)	0.0067(8)	0.0066(9)
C(27)	0.0309(10)	0.0349(13)	0.0339(11)	0.0034(10)	-0.0005(8)	0.0081(9)
C(28)	0.0357(10)	0.0321(13)	0.0236(10)	0.0077(9)	-0.0005(8)	0.0026(9)
C(29)	0.0274(9)	0.0230(11)	0.0228(9)	0.0001(8)	0.0035(7)	-0.0040(8)
C(30)	0.0305(10)	0.0330(13)	0.0244(9)	0.0043(9)	0.0076(8)	-0.0006(8)

References:

1. M. Meyer, W. Paciorek, A. Kowalski, A. Muszynski, A. Wisniewski, M. Pol, M. Przewozniczek, P. Stec, D. Bujnik, H. Kulza, *et al. CrysAlisPro*; Rigaku Oxford Diffraction (1995-2018), **2018**.
2. L. J. Farrugia, *J. Appl. Crystallogr.* **1999**, *32*, 837 – 838.
3. G. M. Sheldrick, *Acta Crystallogr A Found Crystallogr* **2008**, *64*, 112 – 122.
4. C. B. Hübschle, G. M. Sheldrick, B. Dittrich, *J. Appl. Crystallogr.* **2011**, *44*, 1281 – 1284.
5. H. Putz, K. Brandenburg, *Diamond*; Crystal Impact: Bonn, Deutschland, **2014**.
6. (a) P. K. Eckert, V. Schill, C. Strohmann, *Inorg. Chim. Acta* **2011**, *376*, 634 – 637; (b) L. Alakonda, M. Periasamy, *J. Organomet. Chem.* **2009**, *694*, 3859 – 3863; (c) C. Metallinos, X. Du, *Organometallics* **2009**, *28*, 1233 – 1242; (d) S. G. Davies, A. A. Mortlock, *Tetrahedron*, **1993**, *49*, 4419 – 4438.
7. A. Rajca, A. Olankitwanit, S. Rajca, *J. Am. Chem. Soc.* **2011**, *133*, 4750 – 4753.
8. J. R. Struble, J. W. Bode, *Org. Synth.* **2010**, *87*, 362 – 376.
9. J. R. Struble, J. W. Bode, *Tetrahedron* **2008**, *64*, 6961 – 6972.
10. (a) P. Thordarson, *Chem. Soc. Rev.* **2011**, *40*, 1305–1323. (b) http://supramolecular.org
